# Microalbuminuria and mortality in individuals with coronary heart disease: A meta-analysis of a prospective study

**DOI:** 10.1016/j.ihj.2023.05.006

**Published:** 2023-05-18

**Authors:** Ericko Govardi, Dicky Yulianda, Faisal Habib, Cennikon Pakpahan

**Affiliations:** a Faculty of Medicine, University of Sumatera Utara, Medan, Indonesia; b Department of Cardiology and Vascular Medicine, Faculty of Medicine, University of Sumatera Utara, Medan, Indonesia; c Department of Biomedicine, Faculty of Medicine, Universitas Airlangga, Surabaya, Indonesia

**Keywords:** Coronary heart disease, Microalbuminuria, All-cause mortality, Cardiovascular mortality, Meta-analysis

## Abstract

**Aim:**

Microalbuminuria has been elevated as an outcome predictor in cardiovascular medicine. However, due to the small number of studies investigating the association of microalbuminuria and mortality in the coronary heart disease (CHD) population, the prognosis value of microalbuminuria in CHD remains under debate. The objective of this meta-analysis was to investigate the relationship between microalbuminuria and mortality in individuals with CHD.

**Method:**

A comprehensive literature search was performed using Pubmed, EuroPMC, Science Direct, and Google Scholar from 2000 to September 2022. Only prospective studies investigating microalbuminuria and mortality in CHD patients were selected. The pooled effect estimate was reported as risk ratio (RR).

**Results:**

5176 patients from eight prospective observational studies were included in this meta-analysis. Individuals with CHD have a greater overall risk of all-cause mortality (ACM) [*r*R = 2.07 (95% CI = 1.70–2.44); *p* = 0.0003; *I*^2^ = 0.0%] as well as cardiovascular mortality (CVM) [*r*R = 3.23 (95% CI = 2.06–4.39), *p* < 0.0001; *I*^*2*^ = 0.0%]. Subgroup analysis based on follow-up duration and a subset of CHD patients were similarly associated with an increased risk of ACM.

**Conclusion:**

This meta-analysis indicates that microalbuminuria is associated with a higher risk of mortality in individuals with CHD. Microalbuminuria can serve as a predictor of poor outcomes in CHD patients.

## 1 Introduction

Coronary heart disease (CHD), including acute coronary syndrome (ACS) and chronic coronary syndrome (CCS), has become a crucial public health problem in recent decades.^1^ Fortunately, over the past few decades, there has been a continuous decline in the mortality of CHD.^2^, ^3^, ^4^ The decrease in mortality has been ascribed to the expanded use of evidence-based medical therapies as well as population-level improvements in risk factors and lifestyle changes. However, the burden of CHD remains unacceptably high, particularly in developing countries.^5^, ^6^, ^7^

Traditional risk factors do not entirely explain the heterogeneity in cardiovascular disease (CVD) incidence and mortality between individuals and populations, prompting researchers to look at nontraditional cardiovascular risk factors.^8^ These circumstances led to various predictors being created to evaluate the risk of cardiovascular events (including death) in the general population during a specific period of time.^9^, ^10^, ^11^, ^12^

Albumin, the most abundant protein in the body, maintains plasma oncotic pressure, provides sustenance for renal tubular cells, and acts as an antioxidant.^13^ Albumin can be released into the urine due to dysfunction of the glomerular basement membrane (GBM) filtration barrier, and the amount present is important.^14^ The raised concentration of urinary albumin in low concentration, microalbuminuria, is one such factor that has been described independently, which is associated with all-cause mortality (ACM) and cardiovascular mortality (CVM) risk in different populations.^15^, ^16^, ^17^, ^18^, ^19^, ^20^ A 24-h urine sample is the gold standard diagnostic test for the identification of microalbuminuria since it has the lowest variability, is generally available, straightforward, and affordable.^21^

However, due to the small number of studies investigating the association of microalbuminuria and ACM in the CHD population, no meta-analysis was performed before. As a result, the prognostic value of microalbuminuria in CHD remains under debate. Therefore, a meta-analysis was performed to better understand the effect of microalbuminuria on mortality in the CHD population.

## 2 Methods

### 2.1 Search strategy

A systematic literature search was conducted on Pubmed, EuroPMC, Science Direct, and Google scholar databases from 2000 to September 2022 b y two independent investigators (EG and DY) using MESH of the following keywords in combination: “coronary heart disease” AND “microalbuminuria” AND “mortality” OR “death” AND “follow-up”. Each search word was altered to adhere to the limitations of each database. In addition, hand-examined references from relevant papers and reviews were carefully evaluated. This meta-analysis was carried out according to the guideline of the Preferred Reporting Items for Systematic Reviews and Meta-analyses Statement (PRISMA).^22^

### 2.2 Study selection

The eligible prospective observational studies that meet the following criteria were included: (1) population: coronary heart disease without known renal disease adult patients; (2) exposure: microalbuminuria (Microalbuminuria is defined as an albumin excretion rate of 20–200 g/min or an albumin-to-creatinine ratio of 30–300 mg albumin/g creatinine from a spot urine sample or a 24-h sample, or the next comparable period.); (3) comparison: without albuminuria; (4) description risk estimates for the association between microalbuminuria and ACM or CVM, providing at least age and/or gender-adjusted risk ratio (RR) with 95% confidence interval (CI) or providing events of outcome in observation. Any cause of death, regardless of etiology, was defined as ACM. CVM was characterized as mortality caused by myocardial ischemia and infarction, heart failure, cardiac arrest due to another or unknown cause, or cerebrovascular accident.

Exclusion criteria were: (1) case–control, cross-sectional, retrospective cohort designs, review articles, editorials, commentaries, case reports/series, meta-analyses, and conference abstracts; (2) studies in languages other than English; (3) participants from the general population or other particular patients.

### 2.3 Data extraction and quality assessment

The first author's last name, publication year, location, sample size, gender distribution, baseline age, study population, the definition of microalbuminuria, outcome evaluation, adjusted risk ratio (RR) with 95% confidence intervals (CIs), follow-up duration, and adjusted confounders were all taken from each study independently by two authors (EG and CP). The quality of the study and the risk of bias were assessed using a meta-analysis of Observational Studies in Epidemiology (MOOSE) reporting guidelines.^23^^,^^24^ When a criterion was fulfilled, a score of 1 was given. However, 0 was given if the criterion was unclear or not achieved as listed in Table 2. A study with a total score of 5 or above was considered a high-quality study. On the other hand, a study with a total score below 5 was found to be medium or low-quality studies.^25^ Data extraction and quality evaluation disagreements were resolved by discussion with a third reviewer.

### 2.4 Statistical analysis

All statistical analysis were performed using Software for Statistics and Data Science (STATA) version 16.1. The pooled effect size was calculated using multi-adjusted RR with 95% CIs of each study with statistical significance indicated by a *p*-value <0.05. A random effect Der-Simonian Laird model was used to calculate the pooled effect size despite their heterogeneity.

The Cochran's *Q* test's *P* value of less than 0.10 and I^2^ value above 50% were used to determine statistical heterogeneity. To identify the source of bias and provide an ideal, more robust summary across the relevant studies, sub-group analysis was done depending on sample size, population, and follow-up time. A leave-one-out sensitivity analysis was conducted by removing any single study that would produce exaggerated effect sizes, which may distort the overall results. Publication bias was assessed by Begg's test and Egger's test quantitatively and qualitatively.

## 3 Result

### 3.1 Search result and baseline characteristic

**Fig. 1:**
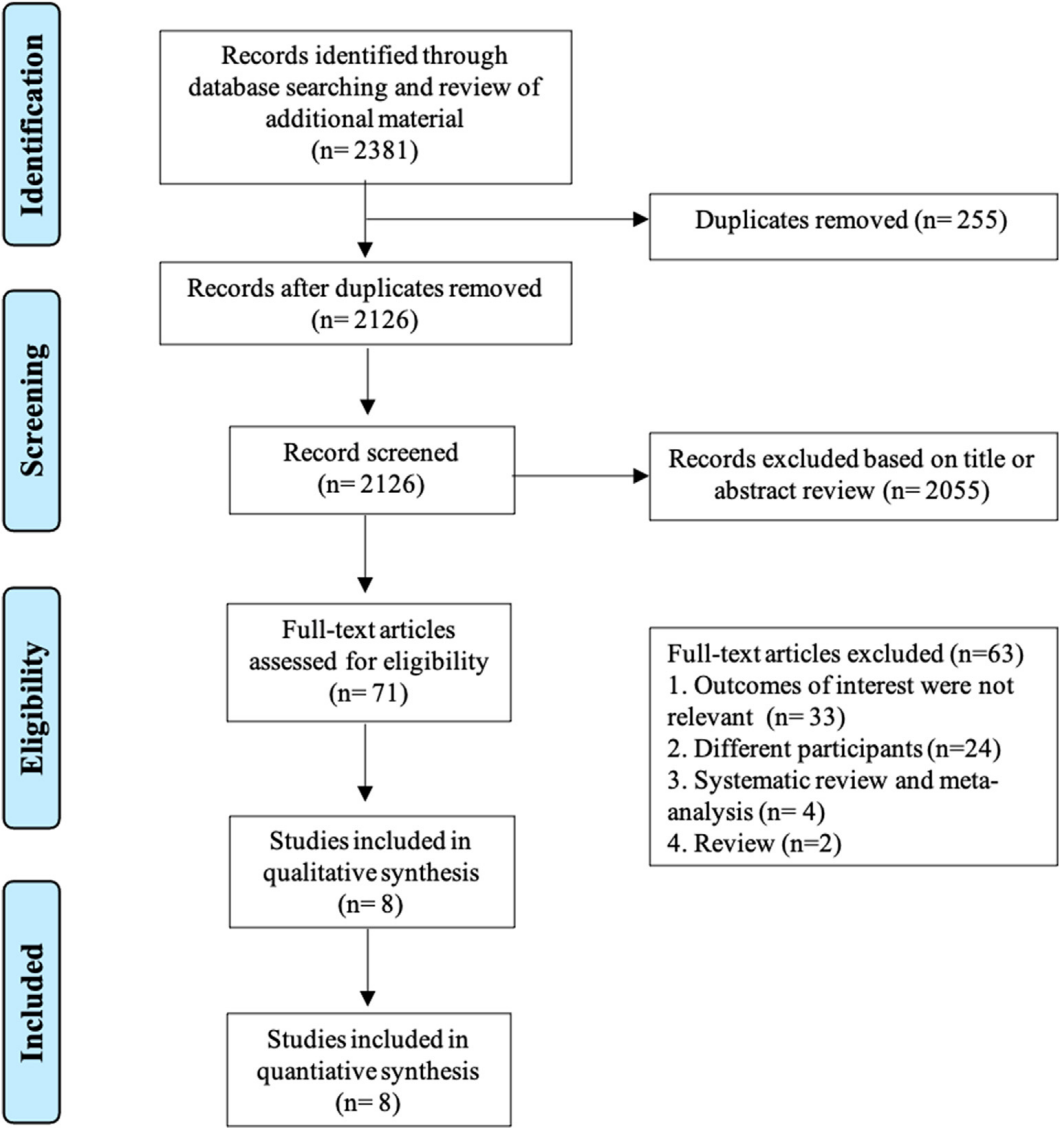
Flow chart of studies selection process.

There were 5176 patients from eight prospective observational studies included in our meta-analysis (Fig. 1).^26^, ^27^, ^28^, ^29^, ^30^, ^31^, ^32^, ^33^ Five studies were conducted on myocardial infarction.^26^, ^27^, ^28^^,^^30^^,^^31^ While one was on ACS,^32^ the other was on patients who received elective percutaneous coronary intervention (PCI).^33^ The characteristics of the individual studies are summarized in Table 1. The qualities of the individual studies are further listed in Table 2.

**Table 1:** Main characteristic of included studies.

Author/year	Country	Sample size (%men)	Median/mean age (years)	Study population	Definition of microalbuminuria	Outcome assessment	Outcome measures RR/HR (95% CI)	Follow-up (days)	Adjustments of covariates
Berton et al 2001^28^	Italy	432 (70%)	64.02 ± 4.21	MI	UACR ≥30 mg/g	All-cause mortality CV mortality	3.7 (2–7)	365	Age, DM, history of angina, CK-MB, killip class, heart rate, thrombolysis, LVEF
Berton et al 2004^29^	Italy	121 (56%)	68.9 ± 10.9	MI and DM	UACR ≥30 ug/mg	All-cause mortality	2.2 (1.7–2.9)	1095	Sex, BMI, current smoking, pre-existing hypertension, prior MI, anterior site of MI
Lekatsas et al 2005^30^	Greece	223 (77%)	61.3 ± 11.5	MI	AER 20–200 ug/min	All-cause mortality	10.5 (1.25–88.19)	26	Sex, hypertension, smoking, previous CAD, use of thrombolysis
Solomon et al 2007^31^	United States, Canada, Italy	2977 (72%)	66.2 ± 8.1	Chronic stable coronary disease, LVEF >40%, age ≥50	UACR 25–354 ug/mg in women and 17–250 ug/mg in men	All-cause mortality CV mortality	All-cause mortality: 2.05 (1.46–2.87) CV mortality: 3.71 (2.18–6.3)	1752	Age, sex, history of MI, DM, LVEF <50%, current smoking, eGFR, BMI
Berton et al 2010^32^	Italy	220 (64%)	64.02 ± 4.21	MI	UACR >20.5 mg/g	All-cause mortality	Short term: 3.9 (2–9) Longterm: 1.8 (1.1–3.1)	Short term: 515 days Long term: 3650 days	Age, HT, DM, pre-hospital time delay, CK-MB, heart failure, creatinine clearance, thrombolysis, LVEF
Taskiran et al 2010^33^	Denmark	151 (86%)	65 (63–68)	MI	UACR >0.65 mg/mmol	All-cause mortality	1.71 (1.03–2.83)	3650	Age, gender
Karki et al 2014^34^	Nepal	134 (66.4%)	63.9	ACS	UACR: 30–300 mg/g	All-cause mortality	1.34 (0.07–24.85)	365	Age, gender
Kunimura et al 2015^35^	Japan	698 (80%)	72 ± 9	Underwent elective PCI	UACR: 30–300 mg/g	CV mortality	2.56 (1.23–5.32)	1564	Age, sex, BMI, LVEF, current smoker, DM, HT, dyslipidemia, eGFR <60 ml/min/1.73 m^2^

*RR* risk ratio; *HR* hazard ratio; *CI* confidence intervals; *UACR* urinary albumin-to-creatinine ratio; *CV* cardiovascular; *DM* diabetes mellitus; *CK*-*MB* creatinine kinase-MB; *LVEF* left ventricular ejection fraction; *BMI* body mass index; *MI* myocardial infarction; *AER* albumin excretion rate; *CAD* coronary artery disease; *eGFR* estimated glomerular filtration rate; *HT* hypertension; *PCI* percutaneous coronary intervention.

**Table 2:** Quality assessment of included studies.

	Berton et al (2001)	Berton et al (2004)	Lekatsas et al (2005)	Solomon et al (2007)	Berton et al (2010)	Taskiran et al (2010)	Karki et al (2014)	Kunimura et al.
Clear inclusion and exclusion of population characteristics	Yes	Yes	Yes	Yes	Yes	Yes	Yes	Yes
Documents loss to follow-up	Yes	Yes	No	Unclear	Yes	Unclear	No	Yes
Prognostic factor of interest is adequately measured	Yes	Yes	Yes	Yes	Yes	Yes	Yes	Yes
Clear definition of outcome	Yes	Yes	Yes	Yes	Yes	Yes	Yes	Yes
Control of confounding	Yes	Yes	Yes	Yes	Yes	Yes	Unclear	Yes
Appropriate statistics	Yes	Yes	Yes	Yes	Yes	Yes	Yes	Yes
Total Points	6	6	5	5	6	5	3	6

### 3.2 All-cause mortality

**Fig. 2:**
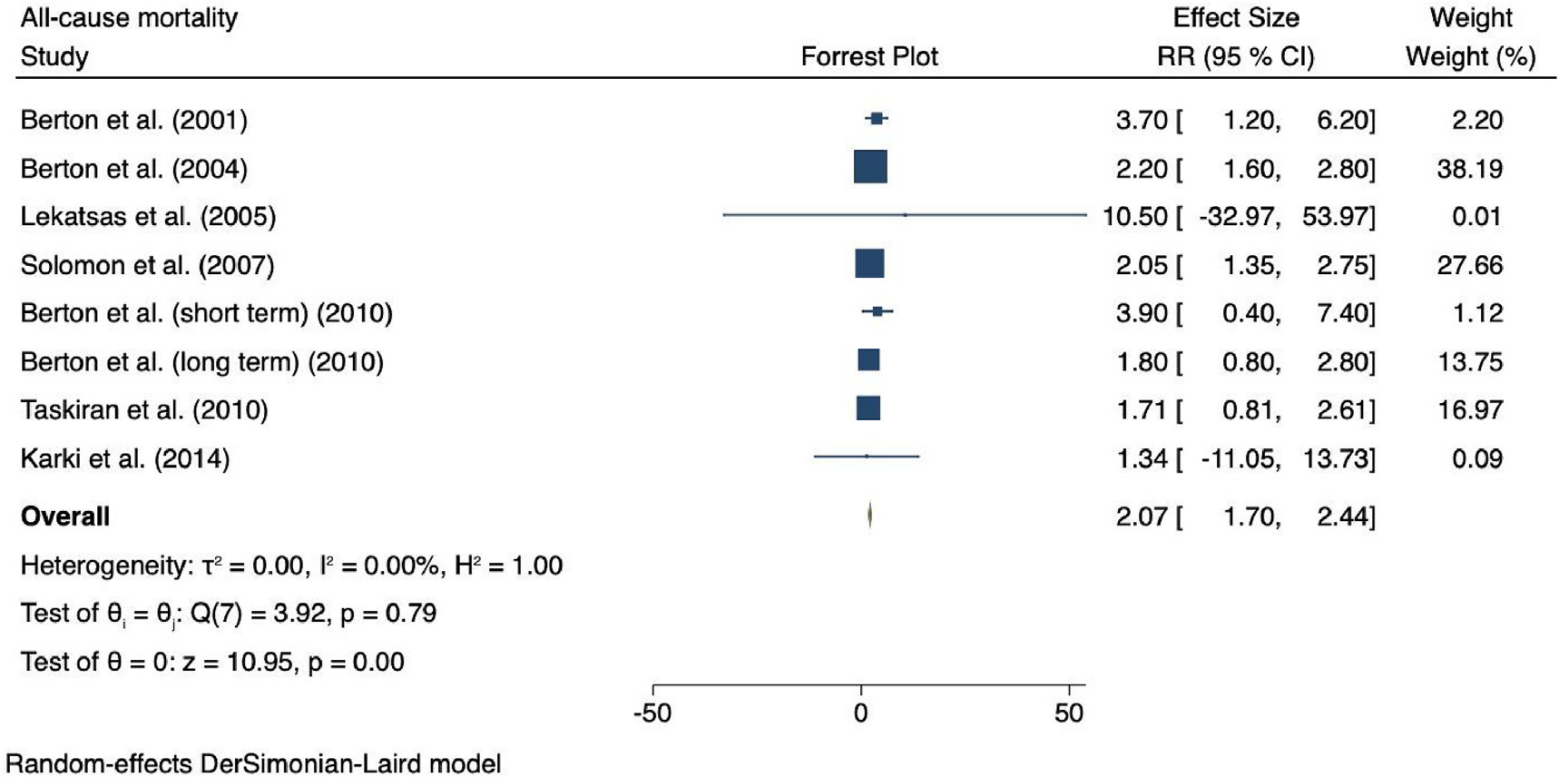
Forest plots showing pooled RR with 95% CI of all-cause mortality. The squares indicate the estimated effect size and the weight of individual studies, respectively. Diamonds indicate the total effect sizes from all studies.

Eight studies were included in the pooled RRs for all-cause mortality, as shown in Fig. 2. Microalbuminuria was associated with a greater risk of ACM (*r*R = 2.07 (95% CI = 1.70–2.44); *p* = 0.0003; *I*^2^ = 0.0%, *p*-heterogeneity = 0.79). Subsequently, regardless of the heterogeneity status, a subgroup analysis was performed based on follow-up duration which showed a considerable increase in those with short follow-up duration (*r*R = 3.72 (95% CI = 1.71–5.72); *I*^*2*^ = 0.0%, *p*-heterogeneity = 0.97). Nonetheless, this remained unchanged in a long follow-up duration (*r*R = 2.01 (95% CI = 1.64–2.39 (; *I*^*2*^ 0.0%, *p*-heterogeneity = 0.8). The statistical significance of pooled risk ratio of ACM was based on sample size subgroup analysis, which revealed that pooled RR were (*r*R = 2.05 (95% CI = 1.55–2.55); *I*^*2*^ = 0.0%, *p*-heterogeneity = 0.67) for smaller sample size, and (*r*R = 2.10 (95% CI = 1.55–2.66); *I*^*2*^ = 0.0%, *p*-heterogeneity = 0.54) for larger sample size. Due to large spectrum of CHD, further analysis was performed based on occurrence of myocardial infarction (MI), which showed pooled RRs of MI and other than MI were (*r*R = 2.08 (95% CI = 1.65–2.52); *I*^*2*^ = 0.0%, *p*-heterogeneity = 0.56) and (*r*R = 2.05 (95% CI = 1.34–2.75); *I*^*2*^ = 0.0%, *p*-heterogeneity = 0.92), respectively **(**Table 3**)**.

**Table 3:** Subgroup analysis on all-cause mortality.

Subgroup	Number of studies	Pooled RR	95% CI	Heterogeneity between studies
Sample size				
<220	3	2.05	1.55–2.55	*p* = 0.67 *I*^*2*^ = 0.0%
≥220	5	2.10	1.55–2.66	*p* = 0.54 *I*^*2*^ = 0.0%
Follow-up duration				
Median <1095 days	4	3.72	1.71–5.72	*p* = 0.97 *I*^*2*^ = 0.0%
Median ≥1095 days	4	2.01	1.64–2.30	*p* = 0.80 *I*^*2*^ = 0.0%
MI				
MI	2	2.05	1.34–2.75	*p* = 0.91 *I*^*2*^ = 0.0%
Other than MI	6	2.08	1.65–2.52	*p* = 0.56 *I*^*2*^ = 0.0%

*RR* risk ratio; *CI* confidence interval; *MI* myocardial infarction.

### 3.3 Cardiovascular mortality

**Fig. 3:**
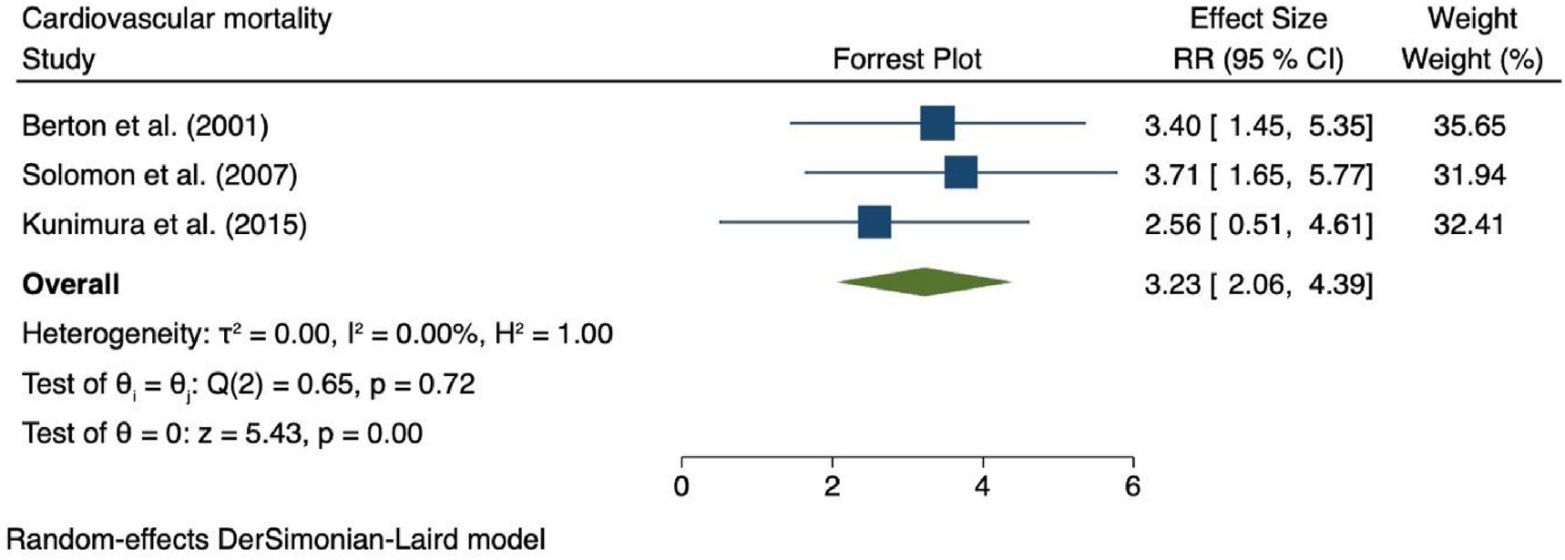
Forest plots showing pooled RR with 95% CI of cardiovascular mortality. Squares indicate the estimated effect size and percentage of the weight of individual studies, respectively. Diamonds indicate the pooled effect sizes from all the studies.

Only three studies reported cardiovascular mortality as an outcome.^26^^,^^29^^,^^33^ There was a substantial increase in risk of CVM in patients with microalbuminuria as shown in Fig. 3 [*r*R = 3.23 (95% CI = 2.06–4.39), *p* < 0.0001; *I*^*2*^ = 0.0%, *p-*heterogeneity = 0.72]. Unfortunately, due to lack of studies, a subgroup analysis was not performed for cardiovascular mortality.

### 3.4 Publication bias and sensitivity analysis

The sensitivity analysis of the leave-one-out study had no effect on the initial statistical significance (data is not shown). There was no evidence of publication bias among studies that reported ACM by Begg's test (*p* = 0.386) and Egger's test (*p* = 0.144), as well as among studies that reported CVM [Begg's test (*p* > 1.00) and Egger's test (*p* = 0.934)].

## 4 Discussion

Regardless of the small number of studies, this is the first meta-analysis to analyze the association between microalbuminuria and mortality in coronary heart disease. The meta-analysis indicates that microalbuminuria confers an increased risk of ACM and CVM. Previous meta-analyses have suggested a link between albuminuria and an increased risk of mortality and poor cardiovascular outcomes in various populations.^25^^,^^34^, ^35^, ^36^ Accordingly, this meta-analysis further added to the value of microalbuminuria as a predictor of ACM and CVM, especially in CHD patients.

The association between microalbuminuria and poor outcomes in CHD may not be surprising. The subgroup study based on the myocardial infarction population and other CHD populations revealed that the statistical significance remained the same in both categories. This demonstrates that microalbuminuria has the same amount of prognostic importance in both populations. Nonetheless, the mechanisms underlying the relationship between microalbuminuria and poor prognosis still need to be understood entirely.

The findings in this study might be explained by a number of different mechanisms. The initial clinical manifestation of glomerular vascular damage, which is a reflection of underlying macrovascular and microvascular disease, is microalbuminuria.^37^ Microalbuminuria was shown to be substantially and independently linked with markers of a prothrombotic condition.^38^^,^^39^ Studies have also suggested an association between microalbuminuria and higher levels of coagulation factors such as tissue factor and factor VII.^40^, ^41^, ^42^ C-reactive protein and fibrinogen may reflect the presence of cytokines which can cause endothelial dysfunction either directly or indirectly.^38^^,^^43^ An increase in these cytokines causes low-grade inflammation that has been shown to cause endothelial dysfunction and microvascular damage.^44^^,^^45^ Endothelial dysfunction and inflammation have been identified as being directly responsible for the atherosclerotic process.^46^ Heparan sulfate proteoglycans, which controls the glomerular basement membrane's permeability and prevents the proliferation of smooth muscle cells, a crucial step in the development of atherosclerosis. This was found to be decreased in microalbuminuria, which has been associated with an increase in capillary permeability.^47^^,^^48^ The rise in inflammatory activity, possibly through the elaboration of proinflammatory cytokines, potentially creates a vicious cycle between inflammatory activity and endothelial dysfunction. The results obtained also correspond with studies that investigated the relationship between microalbuminuria and angiographic severity of coronary artery disease.^49^^,^^50^ The severity of coronary artery lesions or stenosis was considerably greater in individuals with microalbuminuria.^51^ Hence, the interplay of these mechanisms leads to greater risks of adverse outcomes and mortality.

Several studies have provided clinical benefits of albuminuria measurements in predicting disease risk and outcome.^52^, ^53^, ^54^, ^55^, ^56^ Studies that used urine samples to measure microalbuminuria rather than the serum albumin-to-creatinine ratio were included due to their simple method of collection, inexpensive advantage, and widespread availability. However, the serum albumin-to-creatinine ratio was independently associated with adverse outcomes in patients with MI.^57^, ^58^, ^59^, ^60^ The diagnostic performance of measuring UAC in a spot morning urine sample, 24-h, and AUCR in predicting microalbuminuria is also satisfactory.^61^^,^^62^ Hence, we are convinced that, in addition to any other risk stratification employed in evaluation, albuminuria measurement should be taken into account in the assessment of CHD patient's risk for adverse outcomes that might be the focus of preventative interventions.

In addition, this study offers some advantages. All of the included studies have prospective designs, diminishing recall risk and selection bias. Most of the studies in the meta-analysis reported multivariable-adjusted models that increased the study's pooled risk estimate value.

Nonetheless, this meta-analysis has some limitations. First, it is crucial to emphasize that although this association was detected independent of renal function or the presence or absence of a diabetic or hypertensive history, and some of the studies have been adjusted with various covariates, the possibility of other uncontrolled confounding variables could not be ruled out. Individual patient characteristics may impact the pooled risk summary because this is a study-level meta-analysis. Second, the definition of microalbuminuria and cutoff values for UACR were not consistent among studies, thereby affecting their predictive values and the likelihood of misclassification. Third, a meta-regression could not be conducted to associate variables and outcomes of interest in this study, as well as subgroup analysis on the CVM due to the small number of studies. Fourth, the adjusted confounding factors of several included studies limited to only age and/or sex may have provided an overestimation of the risk estimate. Therefore, future prospective studies are needed to address the issues mentioned above.

## 5 Conclusion

According to this meta-analysis, microalbuminuria is associated with an elevated risk of all-cause and cardiovascular mortality in the coronary heart disease population. Thus, close monitoring for microalbuminuria may play an additional role in the prognostic stratification of coronary heart disease patients.

## Author contributors

EG, DY, FH: conception and design of study, data analysis, interpretation. EG, DY, FH CP: literature search, study selection, data extraction, drafting, and revision of the manuscript. EG, DY, FH, CP: read and approved the final version submitted for publication.

## Ethical approval

This page contains no research involving human or animal subjects conducted by any of the authors.

## Declaration of competing interest

The authors declare that they have no known competing financial interests or personal relationships that could have appeared to influence the work reported in this paper.
