# Supplementary material for: Microalbuminuria and mortality in individuals with coronary heart disease: A meta-analysis of a prospective study
Source: Indian Heart J. 2023 May 18;75(4):229–35. doi: 10.1016/j.ihj.2023.05.006 (PMC10421992; doi:10.1016/j.ihj.2023.05.006)
Supplement: Multimedia component 1 [file mmc1.docx]

**Supplemental Material**

The impact of SSRIs on mortality and cardiovascular events in patients with coronary artery disease and depression: systematic review and meta-analysis

Nuno Fernandes, Luísa Prada, Mário Miguel Rosa, Joaquim J Ferreira, João Costa, Fausto J Pinto, Daniel Caldeira

Clinical Research in Cardiology

https://doi.org/10.1007/s00392-020-01697-8

**Corresponding author:** Daniel Caldeira, MD, PhD

Centro Cardiovascular da Universidade de Lisboa - CCUL, Faculdade de Medicina, Universidade de Lisboa, Portugal. Av. Prof. Egas Moniz, Lisboa 1649-028, Portugal

**E-mail:** [dgcaldeira@hotmail.com](mailto:dgcaldeira@hotmail.com).

**Phone number:** (+351) 21 797 34 53; **Fax number:** (+351) 21 781 96 88

**Table of Contents**

[Supplemental Material 1 – Search Strategy 3](#_Toc44366738)

[Supplemental Material 2 – Definition of SSRIs 5](#_Toc44366739)

[Supplemental Material 3 – PRISMA Flow Charts of the Selection of Relevant Studies 11](#_Toc44366740)

[Supplemental Material 4 – Reasons for Exclusion of Articles in the Full-Text Phase 14](#_Toc44366741)

[Supplemental Material 5 – Main Characteristics of Included Studies 23](#_Toc44366742)

[Supplemental Material 6 – Full Characteristics of the Studies Included 24](#_Toc44366743)

[Supplemental Material 7 – Baseline Characteristics of Patients in the Studies Included 27](#_Toc44366744)

[Supplemental Material 8 – Assumptions and Data Treatment for the Meta-analysis 32](#_Toc44366745)

[Supplemental Material 9 – Relevant Outcomes Extracted from the Studies Included 36](#_Toc44366746)

[Supplemental Material 10 – RoB 2 Table of Risk of Bias for the Studies Included 37](#_Toc44366747)

[Supplemental Material 11 – Rob2 Assessment of the RCTs Included 38](#_Toc44366748)

[Supplemental Material 12 – Rob2 Assessment of the FUs Included 47](#_Toc44366749)

[Supplemental Material 13 – List of the Quantitative Analyses Carried Out 51](#_Toc44366750)

[Supplemental Material 14 – Metanalyses Results and Forest Plots 52](#_Toc44366751)

[Supplemental Material 15 – GRADE Evidence Profile 67](#_Toc44366752)

[Supplemental Material 16 – GRADE Summary of Findings 68](#_Toc44366753)

[Supplemental Material 17 – Further Discussion of Limitations 69](#_Toc44366754)

[Supplemental Material 18 – PRISMA Checklist 76](#_Toc44366755)

# Supplemental Material 1 – Search Strategy

We conducted an online electronic search of Cochrane Central Register of Controlled Trials (CENTRAL), Medline (PubMed), and PsycINFO in June 2019 with the Ovid search engine, using the free words and MeSH terms, which returned 562 articles. At that time, we used a narrow definition of SSRIs (see supplemental material 2).

At the end of April 2020, we decided to update our search with the same formula (only articles from 2018 onwards) and conduct a separate search (no publication date limit), including only the SSRIs added to the wider definition (see supplemental material 2). The combination of those two April 2020 searches netted 127 papers.

Below is the resulting net search formula:

Database(s):

- **EBM Reviews - Cochrane Central Register of Controlled Trials** March 2020;
- **Ovid MEDLINE(R) and Epub Ahead of Print, In-Process & Other Non-Indexed Citations, Daily and Versions(R)** 1946 to April 29, 2020;
- **APA PsycInfo** 1967 to April Week 4 2020.

Search Strategy:

| **#** | **Searches** |
| --- | --- |
| 1 | ssri.af |
| 2 | (selective adj2 serotonin adj2 (re-uptake or reuptake) adj2 inhibitor*).af. |
| 3 | exp Serotonin Uptake Inhibitors/ |
| 4 | (citalopram or paroxetine or sertraline or escitalopram or Fluoxetine or Fluvoxamine or Vilazodone or Vortioxetine or Zimelidine or Alaproclate or Etoperidone or Venlafaxine or Desvenlafaxine or Duloxetine or Milnacipran).af. |
| 5 | exp Myocardial Infarction/ |
| 6 | myocardial infarct$.ti,ab. |
| 7 | heart attack$.ti,ab. |
| 8 | heart infarct$.ti,ab. |
| 9 | exp Acute Coronary Syndrome/ |
| 10 | (coronary adj3 syndrome$).ti,ab. |
| 11 | acute coronary.ti,ab. |
| 12 | exp Coronary Thrombosis/ |
| 13 | coronary thrombosis.ti,ab. |
| 14 | ami.ti,ab. |
| 15 | STEMI.ti,ab. |
| 16 | NSTEMI.ti,ab. |
| 17 | exp Angina Pectoris/ |
| 18 | exp Angina, Stable/ |
| 19 | angina.ti,ab. |
| 20 | coronary.ti,ab. |
| 21 | exp Myocardial Ischemia/ |
| 22 | exp Coronary Artery Disease/ |
| 23 | scad.ti,ab. |
| 24 | (stable adj2 coronary adj2 disease).ti,ab. |
| 25 | percutaneous coronary intervention.ti,ab. |
| 26 | 1 or 2 or 3 or 4 |
| 27 | 5 or 6 or 7 or 8 or 9 or 10 or 11 or 12 or 13 or 14 or 15 or 16 or 17 or 18 or 19 or 20 or 21 or 22 or 23 or 24 or 25 |
| 28 | randomized controlled trial.pt. |
| 29 | controlled clinical trial.pt. |
| 30 | randomized.ab. |
| 31 | randomly.ab. |
| 32 | drug therapy.fs. |
| 33 | trial.ti,ab. |
| 34 | groups.ab. |
| 35 | placebo.ab. |
| 36 | clinical trials as topic.sh. |
| 37 | 28 or 29 or 30 or 31 or 32 or 33 or 34 or 35 or 36 |
| 38 | exp animals/ not humans.sh. |
| 39 | 37 not 38 |
| 40 | 26 and 27 and 39 |

# Supplemental Material 2 – Definition of SSRIs

Different sources have different definitions and lists of SSRIs.

For the purposes of your systematic review, we defined SSRIs as: Citalopram, Paroxetine, Sertraline, Escitalopram, Fluoxetine, Fluvoxamine, Vilazodone, Vortioxetine, Zimelidine, Alaproclate, Etoperidone, Venlafaxine, Desvenlafaxine, Duloxetine, or Milnacipran.

At first, we had a narrower definition of SSRIs, but we then decided to expand our definition.

June 2019 search: we defined SSRIs as: Citalopram, Paroxetine, Sertraline, Escitalopram, Fluoxetine, Fluvoxamine, Vilazodone, or Vortioxetine.

We arrived at that list by combining the FDA list of approved SSRIs (available at https://www.fda.gov/drugs/information-drug-class/selective-serotonin-reuptake-inhibitors-ssris-information) with the appropriate SSRIs found on the Mesh Serotonin Uptake Inhibitors [Pharmacological Action] list (Tree Number(s): D017367; MeSH Unique ID: D017367).

| FDA list | |
| --- | --- |
| Citalopram | Included |
| Escitalopram | Included |
| Fluoxetine | Included |
| Fluvoxamine | Included |
| Paroxetine | Included |
| Sertraline | Included |
| Vilazodone | Included |

| Mesh Serotonin Uptake Inhibitors [Pharmacological Action] list (Tree Number(s): D017367; MeSH Unique ID: D017367) | |
| --- | --- |
| alaproclate | Excluded (not currently marketed/development halted) |
| Amoxapine | Excluded (tetracyclic antidepressant; not just serotonin reuptake inhibitor) |
| Citalopram | Already Included |
| Clomipramine | Excluded (tricyclic antidepressant; not just serotonin reuptake inhibitor) |
| femoxetine | Excluded (not currently marketed/development halted) |
| Fenfluramine | Excluded (selective serotonin releasing agent) |
| Fluoxetine | Already Included |
| Fluvoxamine | Already Included |
| indalpine | Excluded (not currently marketed/withdrawn from market) |
| Norfenfluramine | Excluded (serotonin releasing agent, not marketed) |
| Olanzapine | Excluded (atypical antipsychotic) |
| Paroxetine | Already Included |
| Sertraline | Already Included |
| Trazodone | Excluded (not just serotonin reuptake inhibitor) |
| Vilazodone | Already Included |
| Vortioxetine | Included |
| Zimeldine | Excluded (not currently marketed/withdrawn from market) |

April 2020 search: Given that some SNRIs at low dosages have a predominantly serotonin reuptake inhibitor action, we decided to review our definition of SSRIs.

We decided to use the Anatomical Therapeutic Chemical Classification System developed by the World Health Organization and search the ATC code N06A Antidepressants (https://www.whocc.no/atc_ddd_index/?code=N06A) for suitable drugs. As a result, we decided to widen our definition to Citalopram, Paroxetine, Sertraline, Escitalopram, Fluoxetine, Fluvoxamine, Vilazodone, Vortioxetine, Zimelidine, Alaproclate, Etoperidone, Venlafaxine, Desvenlafaxine, Duloxetine, or Milnacipran.

N06AA Non-selective monoamine reuptake inhibitors

| ATC Code | Drug Name | Decision |
| --- | --- | --- |
| N06AA01 | desipramine | Excluded (not selective serotonin reuptake inhibitor) |
| N06AA02 | imipramine | Excluded (not selective serotonin reuptake inhibitor) |
| N06AA03 | imipramine oxide | Excluded (not selective serotonin reuptake inhibitor) |
| N06AA04 | clomipramine | Excluded (not selective serotonin reuptake inhibitor) |
| N06AA05 | opipramol | Excluded (not selective serotonin reuptake inhibitor) |
| N06AA06 | trimipramine | Excluded (not selective serotonin reuptake inhibitor) |
| N06AA07 | lofepramine | Excluded (not selective serotonin reuptake inhibitor) |
| N06AA08 | dibenzepin | Excluded (not selective serotonin reuptake inhibitor) |
| N06AA09 | amitriptyline | Excluded (not selective serotonin reuptake inhibitor) |
| N06AA10 | nortriptyline | Excluded (not selective serotonin reuptake inhibitor) |
| N06AA11 | protriptyline | Excluded (not selective serotonin reuptake inhibitor) |
| N06AA12 | doxepin | Excluded (not selective serotonin reuptake inhibitor) |
| N06AA13 | iprindole | Excluded (not selective serotonin reuptake inhibitor) |
| N06AA14 | melitracen | Excluded (not selective serotonin reuptake inhibitor) |
| N06AA15 | butriptyline | Excluded (not selective serotonin reuptake inhibitor) |
| N06AA16 | dosulepin | Excluded (not selective serotonin reuptake inhibitor) |
| N06AA17 | amoxapine | Excluded (not selective serotonin reuptake inhibitor) |
| N06AA18 | dimetacrine | Excluded (not selective serotonin reuptake inhibitor) |
| N06AA19 | amineptine | Excluded (not selective serotonin reuptake inhibitor) |
| N06AA21 | maprotiline | Excluded (not selective serotonin reuptake inhibitor) |
| N06AA23 | quinupramine | Excluded (not selective serotonin reuptake inhibitor) |

N06AB Selective serotonin reuptake inhibitors

| ATC Code | Drug Name | Decision |
| --- | --- | --- |
| N06AB02 | Zimelidine | Included |
| N06AB03 | Fluoxetine | Already Included |
| N06AB04 | Citalopram | Already Included |
| N06AB05 | Paroxetine | Already Included |
| N06AB06 | Sertraline | Already Included |
| N06AB07 | Alaproclate | Included |
| N06AB08 | Fluvoxamine | Already Included |
| N06AB09 | Etoperidone | Included |
| N06AB10 | Escitalopram | Already Included |

N06AF Monoamine oxidase inhibitors, non-selective

| ATC Code | Drug Name | Decision |
| --- | --- | --- |
| N06AF01 | isocarboxazid | Excluded (not selective serotonin reuptake inhibitor) |
| N06AF02 | nialamide | Excluded (not selective serotonin reuptake inhibitor) |
| N06AF03 | phenelzine | Excluded (not selective serotonin reuptake inhibitor) |
| N06AF04 | tranylcypromine | Excluded (not selective serotonin reuptake inhibitor) |
| N06AF05 | iproniazide | Excluded (not selective serotonin reuptake inhibitor) |
| N06AF06 | iproclozide | Excluded (not selective serotonin reuptake inhibitor) |

N06AG Monoamine oxidase A inhibitors

| ATC Code | Drug Name | Decision |
| --- | --- | --- |
| N06AG02 | moclobemide | Excluded (not selective serotonin reuptake inhibitor) |
| N06AG03 | toloxatone | Excluded (not selective serotonin reuptake inhibitor) |

N06AX Other antidepressants

| ATC Code | Drug Name | Decision |
| --- | --- | --- |
| N06AX01 | Oxitriptan | Excluded (not selective serotonin reuptake inhibitor) |
| N06AX02 | Tryptophan | Excluded (not selective serotonin reuptake inhibitor) |
| N06AX03 | Mianserin | Excluded (not selective serotonin reuptake inhibitor) |
| N06AX04 | Nomifensine | Excluded (not selective serotonin reuptake inhibitor) |
| N06AX05 | Trazodone | Excluded (not selective serotonin reuptake inhibitor) |
| N06AX06 | Nefazodone | Excluded (not selective serotonin reuptake inhibitor) |
| N06AX07 | Minaprine | Excluded (not selective serotonin reuptake inhibitor) |
| N06AX08 | Bifemelane | Excluded (not selective serotonin reuptake inhibitor) |
| N06AX09 | Viloxazine | Excluded (not selective serotonin reuptake inhibitor) |
| N06AX10 | Oxaflozane | Excluded (not selective serotonin reuptake inhibitor) |
| N06AX11 | Mirtazapine | Excluded (not selective serotonin reuptake inhibitor) |
| N06AX12 | Bupropion | Excluded (not selective serotonin reuptake inhibitor) |
| N06AX13 | Medifoxamine | Excluded (not selective serotonin reuptake inhibitor) |
| N06AX14 | Tianeptine | Excluded (not selective serotonin reuptake inhibitor) |
| N06AX15 | Pivagabine | Excluded (not selective serotonin reuptake inhibitor) |
| N06AX16 | Venlafaxine | Included (selective serotonin reuptake inhibitor at low dosages) |
| N06AX17 | Milnacipran | Included (selective serotonin reuptake inhibitor at low dosages) |
| N06AX18 | Reboxetine | Excluded (not selective serotonin reuptake inhibitor) |
| N06AX19 | Gepirone | Excluded (not selective serotonin reuptake inhibitor) |
| N06AX21 | Duloxetine | Included (selective serotonin reuptake inhibitor at low dosages) |
| N06AX22 | Agomelatine | Excluded (not selective serotonin reuptake inhibitor) |
| N06AX23 | Desvenlafaxine | Included (selective serotonin reuptake inhibitor at low dosages) |
| N06AX24 | Vilazodone | Already Included |
| N06AX25 | Hyperici herba | Excluded (not selective serotonin reuptake inhibitor) |
| N06AX26 | Vortioxetine | Already Included |
| N06AX27 | Esketamine | Excluded (not selective serotonin reuptake inhibitor) |

The search was then re-carried out in order to include the added SSRIs. Of note, the updated search did not result in any new studies being included, so the decision to be more inclusive than the FDA definition of SSRI did not alter our results, given that we only found relevant studies with SSRIs that are present in both the FDA and WHO lists.

# Supplemental Material 3 – PRISMA Flow Charts of the Selection of Relevant Studies

June 2019 Search:

Records identified through CENTRAL, Medline, and PsycINFO database search
(n = 562)

Records after duplicates removed
(n = 550)

Records screened
(n = 550)

Records excluded
(n = 404)

Full-text articles assessed for eligibility
(n = 146)

Full-text articles excluded (n=136):

- Wrong publication type (n=43)
- Lack of outcomes (n=30)
- Wrong population (n=24)
- Wrong control (n=15)
- Wrong study design (n=13)
- Wrong intervention (n=8)
- Repeated (n=3)

Studies included in the qualitative syntheses
(n = 8+2)*

Studies included in the primary quantitative syntheses (meta-analyses)
(n =6+2)**

PRISMA Flow Chart of the identification and selection of relevant studies (June 2019 search). Based on^22^. *8 RCTs and 2 FUs of RCTs already included. **6 RCTs and 2 FUs

April 2020 Search:

Records identified through CENTRAL, Medline, and PsycINFO database search
(n = 127)

Records after duplicates removed
(n = 125)

Records screened
(n = 125)

Records excluded
(n = 57)

Full-text articles assessed for eligibility
(n = 68)

Full-text articles excluded (n=68):

- Wrong publication type (n=23)
- Wrong study design (n=19)
- Wrong intervention (n=8)
- Repeated (n=8)
- Wrong population (n=6)
- Lack of outcomes (n=2)
- Wrong control (n=2)

Studies included in the qualitative syntheses
(n = 0)

Studies included in the primary quantitative syntheses (meta-analyses)
(n = 0)

PRISMA Flow Chart of the identification and selection of relevant studies (April 2020 search). Based on^22^.

Net Search:

Records identified through CENTRAL, Medline, and PsycINFO database search
(n = 689)

Records after duplicates removed
(n = 675)

Records screened
(n = 675)

Records excluded
(n = 461)

Full-text articles assessed for eligibility
(n = 214)

Full-text articles excluded (n=204):

- Wrong publication type (n=66)
- Lack of outcomes (n=32)
- Wrong study design (n=32)
- Wrong population (n=30)
- Wrong control (n=17)
- Wrong intervention (n=16)
- Repeated (n=11)

Studies included in the qualitative syntheses
(n = 8+2)*

Studies included in the primary quantitative syntheses (meta-analyses)
(n =6+2)**

PRISMA Flow Chart of the identification and selection of relevant studies (net search). Based on^22^. *8 RCTs and 2 FUs of RCTs already included. **6 RCTs and 2 FUs

# Supplemental Material 4 – Reasons for Exclusion of Articles in the Full-Text Phase

If multiple reasons for exclusion, only one was registered (under no specific hierarchical order).

June 2019 Search:

| Effects of paroxetine-mediated inhibition of GRK2 expression on depression and cardiovascular function in patients with myocardial infarction | Repeated (same RCT as Tian) |
| --- | --- |
| [Depression after first myocardial infarction. A prospective study on incidence, prognosis, risk factors and treatment] | Repeated (same RCT as Strik); we could not retrieve the article; decision based on the abstract |
| Social support deficit and depression treatment outcomes in patients with acute coronary syndrome: findings from the EsDEPACS study | Repeated study (same as Kim) and wrong outcome |
| "Cardiac side-effects of two selective serotonin reuptake inhibitors in middle-aged and elderly depressed patients": Erratum | Wrong control (2 SSRIs being compared: fluvoxamine vs fluoxetine) |
| Cardiac side-effects of two selective serotonin reuptake inhibitors in middle-aged and elderly depressed patients | Wrong control (2 SSRIs being compared: fluvoxamine vs fluoxetine) |
| Clinical activity and tolerability of trazodone, mianserin, and amitriptyline in elderly subjects with major depression: a controlled multicentre trial | Wrong control (not placebo or no intervention) |
| Clinical efficacy and safety of the Shugan Jieyu capsule in patients with acute myocardial infarction and depression | Wrong control (for the purposes of our review, Shugan Jieyu was not considered “placebo” nor “no intervention”, because it has been linked to reduced depressive symptoms in some studies) |
| Comparison of paroxetine and nortriptyline in depressed patients with ischemic heart disease | Wrong control (nortriptyline) |
| Major depression with ischemic heart disease: effects of paroxetine and nortriptyline on long-term heart rate variability measures | Wrong control (nortriptyline) |
| Major depression with ischemic heart disease: effects of paroxetine and nortriptyline on measures of nonlinearity and chaos of heart rate | Wrong control (nortriptyline) |
| Paroxetine Versus Nortriptyline in Ischemic Disease CONFERENCE ABSTRACT | Wrong control (nortriptyline) |
| Resistin and adiponectin in major depression: the association with free cortisol and effects of antidepressant treatment | Wrong control (amitriptyline) |
| Trazodone in late life depressive states: a double-blind multicenter study versus amitriptyline and mianserin | Wrong control (not placebo or no intervention) |
| Treatment of major depression with nortriptyline and paroxetine in patients with ischemic heart disease | Wrong control (nortriptyline) |
| A randomized, double-blind, clinical trial comparing the efficacy and safety of Crocus sativus L. with fluoxetine for improving mild to moderate depression in post percutaneous coronary intervention patients | Wrong control (for the purposes of our review, saffron was not considered “placebo” nor “no intervention”, because it has been linked to reduced depressive symptoms in some studies) |
| Interventional effect of fluoxetine on patients with acute myocardial infarction accompanied by anxiety and depression | Wrong control – we could not retrieve the article; excluded based on the abstract (for the purposes of our review, sheirkang was not considered “placebo” nor “no intervention”) |
| Paroxetine and amitriptyline in the treatment of depression in general practice | Wrong control (amitriptyline) |
| Antidepressant treatment and dehydroepiandrosterone sulfate: different effects of amitriptyline and paroxetine | Wrong control (amitriptyline) |
| Somatic and sociodemographic predictors of depression outcome among depressed patients with coronary artery disease-A exploratory analysis of the SPIRR-CAD study | Wrong intervention (psychotherapy) |
| Depression and late mortality after myocardial infarction in the Enhancing Recovery in Coronary Heart Disease (ENRICHD) study | Wrong intervention (intervention not exclusively SSRI; only the patients with more severe depressions or that did not respond to psychotherapy were given SSRIs, so there is a selection bias for the SSRI subgroup; as such, there is no real randomization between SSRI and no SSRI) |
| Antidepressive effect of mirtazapine in post-myocardial infarction depression is associated with soluble TNF-R1 increase: Data from the MIND-IT | Wrong intervention (for the purposes of our review, mirtazapine was not considered an SSRI) |
| Effects of antidepressant treatment following myocardial infarction | Wrong intervention (for the purposes of our review, mirtazapine was not considered an SSRI; first there was a randomization between intervention and care as usual. The patients randomized to the intervention then choose if they wanted to (1) take part in a nested double-blind mirtazapine-placebo, in which if they didn’t respond to mirtazapine or placebo they were given open treatment with an SSRI (citalopram); (2) open treatment with SSRI (citalopram); (3) non-pharmacological treatment; or (4) no treatment. Patients in the care as usual group were not informed about their diagnosis of depression so as not to influence usual care but could receive depression treatment (pharmacological or non-pharmacological), if provided to them by their physicians. There is no real randomization between SSRIs and no SSRIs. |
| Depression treatment after myocardial infarction and long-term risk of subsequent cardiovascular events and mortality: a randomized controlled trial |  |
| Treatment of post-myocardial infarction depressive disorder: a randomized, placebo-controlled trial with mirtazapine |  |
| Effects of antidepressant medication on morbidity and mortality in depressed patients after myocardial infarction | Wrong intervention (the trial compares care as usual with an intervention. The intervention is psychotherapy, but the patients that had severe depression and the ones that did not improve with psychotherapy were given SSRIs. Some patients in both the care as usual and the intervention groups were already taking antidepressants and some patients in the usual care group were prescribed antidepressants by their physicians. There is no comparison posted regarding the subgroup "severe depression" SSRI vs "severe depression" Care as usual. The comparisons made between antidepressant use and no antidepressant use include people from both the usual care and the intervention groups. There is no real randomization to a SSRI group and a no SSRI group. |
| [The influence of antidepressive therapy on short-term prognosis in elderly patients with unstable angina and depression] | Wrong intervention (SSRI + psychotherapy) |
| [Clinical efficacy of escitalopram in patients with ischemic heart disease and comorbid depression] | No relevant outcomes identified |
| Associations between Serotonergic Genes and Escitalopram Treatment Responses in Patients with Depressive Disorder and Acute Coronary Syndrome: The EsDEPACS Study | No relevant outcomes identified |
| BDNF val66met polymorphism and depressive disorders in patients with acute coronary syndrome | No relevant outcomes identified |
| Cognitive performance following fluoxetine treatment in depressed patients post myocardial infarction | No relevant outcomes identified |
| Correlates and Escitalopram Treatment Effects on Sleep Disturbance in Patients with Acute Coronary Syndrome: K-DEPACS and EsDEPACS | No relevant outcomes identified |
| Determinants and escitalopram treatment effects on suicidal ideation in patients with acute coronary syndrome: Findings from the K-DEPACS and EsDEPACS studies | No relevant outcomes identified |
| Effects of depression co-morbidity and treatment on quality of life in patients with acute coronary syndrome: the Korean depression in ACS (K-DEPACS) and the escitalopram for depression in ACS (EsDEPACS) study | No relevant outcomes identified |
| Effects of depression screening on psychiatric outcomes in patients with acute coronary syndrome: Findings from the K-DEPACS and EsDEPACS studies | No relevant outcomes identified |
| Effects of Escitalopram on Anxiety in Patients with Acute Coronary Syndrome: A Randomized Controlled Trial | No relevant outcomes identified |
| Effects of selective serotonin reuptake inhibitor therapy on endothelial function and inflammatory markers in patients with coronary heart disease | No relevant outcomes identified |
| Efficacy of citalopram in improving depression and quality of life in acute coronary syndrome | No relevant outcomes identified |
| First episode of major depressive disorder and vascular factors in coronary artery disease patients: Baseline characteristics and response to antidepressant treatment in the CREATE trial | No relevant outcomes identified |
| Genetic association with suicidal ideation in patients with acute coronary syndrome | No relevant outcomes identified |
| Heart rate variability in acute coronary syndrome patients with major depression: influence of sertraline and mood improvement | No relevant outcomes identified |
| Influence of depression and effect of treatment with sertraline on quality of life after hospitalization for acute coronary syndrome | No relevant outcomes identified |
| Influences of the Big Five personality traits on the treatment response and longitudinal course of depression in patients with acute coronary syndrome: A randomised controlled trial | No relevant outcomes identified |
| Methylation of the glucocorticoid receptor gene associated with depression in patients with acute coronary syndrome | No relevant outcomes identified |
| Onset of major depression associated with acute coronary syndromes: relationship of onset, major depressive disorder history, and episode severity to sertraline benefit | No relevant outcomes identified |
| Platelet and endothelial activity in comorbid major depression and coronary artery disease patients treated with citalopram: the Canadian Cardiac Randomized Evaluation of Antidepressant and Psychotherapy Efficacy Trial(CREATE) biomarker sub-study | No relevant outcomes identified |
| Results of the responses of myocardial ischemia to escitalopram treatment trial | No relevant outcomes identified |
| Social support deficit and depression treatment outcomes in patients with acute coronary syndrome: Findings from the EsDEPACS study | No relevant outcomes identified |
| The effection of paroxetine on PAI-1 and ET in patients with AMI who comblined with anxiety and depression after PCI | No relevant outcomes identified |
| Understanding prognostic benefits of exercise and antidepressant therapy for persons with depression and heart disease: the UPBEAT study--rationale, design, and methodological issues | No relevant outcomes identified |
| Using the Hospital Anxiety and Depression Scale to screen for depression in cardiac patients | No relevant outcomes identified |
| Effects of Exercise and Sertraline on Measures of Coronary Heart Disease Risk in Patients With Major Depression: Results From the SMILE-II Randomized Clinical Trial | No relevant outcomes identified |
| Exercise and pharmacological treatment of depressive symptoms in patients with coronary heart disease: results from the UPBEAT (Understanding the Prognostic Benefits of Exercise and Antidepressant Therapy) study | No relevant outcomes identified (1 withdrew due to illness; 2 due to side effects, 1 due to personal reasons; 2 for unknown reasons; specific reasons not specified) |
| BDNF methylation and depressive disorder in acute coronary syndrome: The K-DEPACS and EsDEPACS studies | No relevant outcomes identified (20 died; not reported by arm); also, same as Kim |
| Predictors of depressive disorder following acute coronary syndrome: Results from K-DEPACS and EsDEPACS | No relevant outcomes identified (26 died; not reported by arm); also, same as Kim. |
| Platelet/endothelial biomarkers in depressed patients treated with the selective serotonin reuptake inhibitor sertraline after acute coronary events: the Sertraline AntiDepressant Heart Attack Randomized Trial (SADHART) Platelet Substudy | No relevant outcomes identified |
| Relationship between release of platelet/endothelial biomarkers and plasma levels of sertraline and N-desmethylsertraline in acute coronary syndrome patients receiving SSRI treatment for depression | No relevant outcomes identified |
| Whole blood serotonin and platelet activation in depressed post-myocardial infarction patients | No relevant outcomes identified |
| Antidepressant therapy in patients undergoing coronary artery bypass grafting: the MOTIV-CABG trial | Wrong population (not only depressed or anxiety disorder patients; relevant outcomes not stratified by depression status) |
| Efficacy and safety of vilazodone in major depressive disorder: a randomized, double-blind, placebo-controlled trial | Wrong population (depressed patients; not just depressed patients with coronary disease) |
| Elevated stress-hemoconcentration in major depression is normalized by antidepressant treatment: exploratory analysis from a randomized, double-blind clinical trial and relevance to cardiovascular disease risk | Wrong population (depressed patients; not just depressed patients with coronary disease) |
| Lipid metabolism and insulin resistance in depressed patients: significance of weight, hypercortisolism, and antidepressant treatment | Wrong population (depressed patients; not just depressed patients with coronary disease) |
| Noradrenergic dysfunction and antidepressant treatment response | Wrong population (depressed patients; not just depressed patients with coronary disease) |
| Open-label non-randomized versus double-blind randomized antidepressive treatment: what are the advantages of clinical decision over randomization? | Wrong population (depressed patients; not just depressed patients with coronary disease) |
| Safety and efficacy of sertraline for depression in patients with CHF (SADHART-CHF): a randomized, double-blind, placebo-controlled trial of sertraline for major depression with congestive heart failure | Wrong population (heart failure, not stratified by aetiology of HF) |
| Trajectory classes of depression in a randomized depression trial of heart failure patients: a reanalysis of the SADHART-CHF trial | Wrong population (heart failure, not stratified by aetiology of HF) |
| Cardiovascular safety of one-year escitalopram therapy in clinically nondepressed patients with acute coronary syndrome: results from the DEpression in patients with Coronary ARtery Disease (DECARD) trial | Wrong population (non-depressed; prevention of depression) |
| Effects of escitalopram in prevention of depression in patients with acute coronary syndrome (DECARD) | Wrong population (non-depressed; prevention of depression) |
| Effects of escitalopram in prevention of depression in patients with acute coronary syndrome (DECARD): randomised controlled trial | Wrong population (non-depressed; prevention of depression) |
| Effects of escitalopram in prevention of depression in patients with acute coronary syndrome (DECARD): randomised controlled trial | Wrong population (non-depressed; prevention of depression) |
| Escitalopram prevents depression in patients with acute coronary syndrome (DECARD): a randomized controlled trial | Wrong population (non-depressed; prevention of depression) |
| Escitalopram prevents depression in patients with acute coronary syndrome (DECARD): a randomized controlled trial[conference abstract] | Wrong population (non-depressed; prevention of depression) |
| Prevention of depression in patients with acute coronary syndrome (DECARD) randomized trial: effects on and by self-reported health | Wrong population (non-depressed; prevention of depression) |
| Rationale, design and methodology of a double-blind, randomized, placebo-controlled study of escitalopram in prevention of Depression in Acute Coronary Syndrome (DECARD) | Wrong population (non-depressed; prevention of depression) |
| The role of the selective serotonin re-uptake inhibitor sertraline in nondepressive patients with chronic ischemic heart failure: a preliminary study | Wrong population (non-depressed; prevention of depression) |
| Mianserin and trazodone for cardiac patients with depression | Wrong population (not just ischemic coronary disease) |
| Usefulness of Myocardial Annular Velocity Change During Mental Stress to Predict Cardiovascular Outcome in Patients With Coronary Artery Disease (From the Responses of Mental Stress-Induced Myocardial Ischemia to Escitalopram Treatment Trial) | Wrong population (for the purposes of our review, mental stress was not considered to be equivalent to anxiety or depression) |
| Mental stress-induced left ventricular dysfunction and adverse outcome in ischemic heart disease patients | Wrong population (for the purposes of our review, mental stress was not considered to be equivalent to anxiety or depression) |
| Depressive symptoms and mental stress-induced myocardial ischemia in patients with coronary heart disease | Wrong population (for the purposes of our review, mental stress was not considered to be equivalent to anxiety or depression) |
| A Trial of Inflammatory Markers, Depressive Symptoms, and Heart Disease | Wrong population (registry; no results posted; Exclusion Criteria: History of coronary artery disease) |
| Effect of escitalopram on mental stress-induced myocardial ischemia: results of the REMIT trial | Wrong population (no baseline depression or anxiety disorder; for the purposes of our review, mental stress was not considered to be equivalent to anxiety or depression) |
| "Design and Rationale for a Randomized, Controlled Trial of Interpersonal Psychotherapy and Citalopram for Depression in Coronary Artery Disease (CREATE)": Erratum | Wrong publication type (design); refers to Lespérance |
| Antidepressants may temper heart-disease risk | Wrong publication type (we could not retrieve the article, however, it is not the type of publication that would report the results of a clinical trial) |
| Emotions and heart disease | Wrong publication type (we could not retrieve the article, however, it is not the type of publication that would report the results of a clinical trial) |
| Gateways to clinical trials | Wrong publication type (we could not retrieve the article, however, it is not the type of publication that would report the results of a clinical trial) |
| Gateways to clinical trials | Wrong publication type (we could not retrieve the article, however, it is not the type of publication that would report the results of a clinical trial) |
| Pharmacoeconomic analysis of sertraline treatment of depression in patients with unstable angina or a recent myocardial infarction | Wrong publication type – we could not retrieve the article, however, it was excluded based on the fact it was an economic analysis based on SADHART |
| Safe treatment for depression after heart attack | Wrong publication type (we could not retrieve the article, however, it is not the type of publication that would report the results of a clinical trial) |
| Some antidepressants may reduce heart attack risk | Wrong publication type (we could not retrieve the article, however, it is not the type of publication that would report the results of a clinical trial) |
| Treating depression after a heart attack | Wrong publication type (we could not retrieve the article, however, it is not the type of publication that would report the results of a clinical trial) |
| Depression, coronary events, platelet inhibition, and serotonin reuptake inhibitors | Wrong publication type (background article) |
| [Clinical management of patients with depression and cardiac disease] | Wrong publication type (background article) |
| The Canadian cardiac randomized evaluation of antidepressant and psychotherapy efficacy (CREATE) trial | Wrong publication type (book; comments on Lespérance) |
| [Serious QT interval prolongation associated with velafaxine administration] | Wrong publication type (case report) |
| An angry bed partner | Wrong publication type (case report) |
| effects of trazodone and desipramine on cardiac rate and rhythm in a patient with preexisting cardiovascular disease | Wrong publication type (case report) |
| Sudden chest pain with sertraline | Wrong publication type (case report) |
| Selective serotonin reuptake inhibitors may interfere with the antiplatelet effect of clopidogrel | Wrong publication type (comment on an observational study) |
| Coronary artery disease: Antidepressant treatment for mental stress-induced myocardial ischaemia | Wrong publication type (comment on an excluded study) |
| Use of selective serotonin reuptake inhibitors and myocardial infarction | Wrong publication type (comment on a case-control study |
| Therapy for mental stress-induced myocardial ischemia | Wrong publication type (comment on an excluded study) |
| Therapy for mental stress-induced myocardial ischemia | Wrong publication type (comment on an excluded study) |
| Therapy for mental stress-induced myocardial ischemia--reply | Wrong publication type (comment on an excluded study) |
| Correction: effect of escitalopram vs placebo treatment for depression on long-term cardiac outcomes in patients with acute coronary syndrome: a randomized clinical trial (JAMA - Journal of the American Medical Association (2018) 320: 4 (350-358) DOI: 10.1001/jama.2018.9422) | Wrong publication type (correction; refers to Kim) |
| Design and Methodology for the Korean Observational and Escitalopram Treatment Studies of Depression in Acute Coronary Syndrome: K-DEPACS and EsDEPACS | Wrong publication type (design; refers to Kim) |
| Treatment of depression after myocardial infarction and the effects on cardiac prognosis and quality of life: rationale and outline of the Myocardial INfarction and Depression-Intervention Trial (MIND-IT) | Wrong publication type (design of an excluded study) |
| Responses of mental stress-induced myocardial ischemia to escitalopram treatment: background, design, and method for the Responses of Mental Stress Induced Myocardial Ischemia to Escitalopram Treatment trial | Wrong publication type (design of an excluded study) |
| Comparison of the Effects of Religious Cognitive Behavioral Therapy (RCBT), Cognitive Behavioral Therapy (CBT), and Sertraline on Depression and Anxiety in Patients after Coronary Artery Bypass Graft Surgery: Study Protocol for a Randomized Controlled Trial | Wrong publication type (design; no results found) |
| Treatment of anxiety in patients with coronary heart disease: Rationale and design of the UNderstanding the benefits of exercise and escitalopram in anxious patients WIth coroNary heart Disease (UNWIND) randomized clinical trial | Wrong publication type (design, no results posted yet; estimated date of completion July 2020) |
| Design and rationale for a randomized, controlled trial of interpersonal psychotherapy and citalopram for depression in coronary artery disease (CREATE) | Wrong publication type (design; Lespérance) |
| Depression following myocardial infarction | Wrong publication type (editorial; comments on an irrelevant study) |
| [The myocardial infarct patient. Screening for depression and treatment] | Wrong publication type - News (we could not retrieve the article, however, it is not the type of publication that would report the results of a clinical trial) |
| EuroPRevent 2010 | Wrong publication type (non-relevant abstracts) |
| Comparison of therapeutic effect of sertraline and supportive psychotherapy in comparison with placebo in coronary heart disease patients with mild to moderate depression | Wrong publication type (registry; still recruiting) |
| Netherlands Heart Foundation's Myocardial INfarction and Depression-Intervention Trial: effects of antidepressant treatment following myocardial infarction | Wrong publication type (registry of an excluded study) |
| MOTIV Study- Effect of Antidepressive Treatment by Escitalopram in Patients Undergoing Coronary Artery Bypass Grafting | Wrong publication type (registry of an excluded study) |
| "Effects of citalopram and interpersonal psychotherapy on depression in patients with coronary artery disease": Reply | Wrong publication type (reply to Lespérance) |
| Acute Myocardial Necrosis and Depression: antiplatelet Effect of Reuptake Inhibition of Serotonin | Wrong publication type (registry; no results posted; only 2 patients were enrolled) |
| [Stabilizing affective symptoms without adverse cardiac effects. Myocardial infarct risk after depression--and vice versa] | Wrong publication type (review) - we could not retrieve the article; however, we contacted the author |
| Antidepressant drugs and the cardiovascular system: a comparison of tricylics and selective serotonin reuptake inhibitors and their relevance for the treatment of psychiatric patients with cardiovascular problems | Wrong publication type (review) |
| When prevention is a bad idea: Problems with the DECARD trial and the premise behind it | Wrong publication type (comment on an excluded study) |
| Treatment of depression following acute myocardial infarction | Wrong publication type (editorial on Glassman) |
| Enhancing recovery in coronary heart disease patients (ENRICHD): study design and methods. The ENRICHD investigators | Wrong publication type (design of an excluded study) |
| Session highlights from the American College of Cardiology Scientific Sessions: March 29 to April 1. 1998 | Wrong publication type (a group of studies; none of them relevant) |
| Changes in cognitive versus somatic symptoms of depression and event-free survival following acute myocardial infarction in the Enhancing Recovery In Coronary Heart Disease (ENRICHD) study | Wrong study design (exploratory analysis); also, related to an excluded study |
| Excess risk of MI in patients treated with antidepressant medications | Wrong study design (observational cohort) |
| History of depression and survival after acute myocardial infarction | Wrong study design (exploratory analysis); also, related to an excluded study |
| Nonresponse to treatment for depression following myocardial infarction: association with subsequent cardiac events | Wrong study design (exploratory analysis); also, related to an excluded study |
| Predictors of treatment response for depression and inadequate social support--the ENRICHD randomized clinical trial | Wrong study design (exploratory analysis); also, related to an excluded study |
| Safety of selective serotonin reuptake inhibitor in adults undergoing coronary artery bypass grafting | Wrong study design (observational cohort) |
| Safety of selective serotonin reuptake inhibitor use prior to coronary artery bypass grafting | Wrong study design (observational cohort) |
| Selective Serotonin Reuptake Inhibitors and Serotonin-Norepinephrine Reuptake Inhibitors Are Not Associated With Bleeding or Transfusion in Cardiac Surgical Patients | Wrong study design (observational cohort) |
| Selective serotonin reuptake inhibitors, venlafaxine and duloxetine are associated with in hospital morbidity but not bleeding or late mortality after coronary artery bypass graft surgery | Wrong study design (observational cohort) |
| Serotonin reuptake inhibitor use, depression, and long-term outcomes after an acute coronary syndrome: a prospective cohort study | Wrong study design (observational cohort) |
| Sertraline treatment for depression associated with acute coronary syndromes: a cost analysis from the viewpoint of the Italian Healthcare System | Wrong study design (an economic analysis of Glassman) |
| Impact of Suicidal Ideation on Long-Term Cardiac Outcomes in Patients with Acute Coronary Syndrome: sex-Specific Differences | Wrong study design (observational cohort), not stratified by participation in RCT |
| An open-label preliminary trial of sertraline for treatment of major depression after acute myocardial infarction (the SADHAT Trial). Sertraline Anti-Depressant Heart Attack Trial | Wrong study design (not randomized) |

April 2020 Search:

| First episode of major depressive disorder and vascular factors in coronary artery disease patients: Baseline characteristics and response to antidepressant treatment in the CREATE trial | No relevant outcomes identified |
| --- | --- |
| Efficacy of citalopram in improving depression and quality of life in acute coronary syndrome | No relevant outcomes identified |
| Social support deficit and depression treatment outcomes in patients with acute coronary syndrome: findings from the EsDEPACS study | Repeated study (same as Kim) and wrong outcome |
| Social support deficit and depression treatment outcomes in patients with acute coronary syndrome: findings from the EsDEPACS study | Repeated study (same as Kim) and wrong outcome |
| Effect of Escitalopram vs Placebo Treatment for Depression on Long-term Cardiac Outcomes in Patients With Acute Coronary Syndrome: a Randomized Clinical Trial | Repeated (Already included) |
| Effect of escitalopram vs placebo treatment for depression on long-term cardiac outcomes in patients with acute coronary syndrome: A randomized clinical trial | Repeated (Already included) |
| Effects of citalopram and interpersonal psychotherapy on depression in patients with coronary artery disease: The Canadian Cardiac Randomized Evaluation of Antidepressant and Psychotherapy Efficacy (CREATE) trial | Repeated (Already included) |
| Long-term cardiac outcomes of depression screening, diagnosis and treatment in patients with acute coronary syndrome: the DEPACS study | Repeated (excluded) |
| Modifying effects of depression on the association between BDNF methylation and prognosis of acute coronary syndrome | Repeated (excluded) |
| Association between obsessive-compulsive symptoms and long-term cardiac outcomes in patients with acute coronary syndrome: effects of depression comorbidity and treatment | Repeated (excluded) |
| The Bypassing the Blues treatment protocol: Stepped collaborative care for treating post-CABG depression | Wrong intervention (collaborative care) |
| Effects of saffron on cognition, anxiety, and depression in patients undergoing coronary artery bypass grafting: A randomized double-blind placebo-controlled trial | Wrong intervention (saffron) |
| A Chinese herbal formula shows beneficial effects on comorbid depression and coronary heart disease based on the philosophy of psycho-cardiology | Wrong control (one group was given escitalopram and the other was given a Chinese herbal medicine, which, for the purposes of our review, we did not consider to be equivalent to “placebo” or “no |
| Effect of Zaoren Anshen Priscription combined with escitalopram in treatment of coronary heart disease with anxiety disorder and its mechanism on neurotransmitters | Wrong control (one group was given escitalopram and the other was given a Chinese herbal medicine, which, for the purposes of our review, we did not consider to be equivalent to “placebo” or “no intervention”) |
| Design and baseline data from the Management of Sadness and Anxiety in Cardiology (MOSAIC) randomized controlled trial | Wrong intervention (collaborative care) |
| Antidepressive effect of mirtazapine in post-myocardial infarction depression is associated with soluble TNF-R1 increase: Data from the MIND-IT | Wrong intervention (for the purposes of our review, mirtazapine was not considered an SSRI) |
| A Randomized Placebo-Controlled Trial of Omega-3 and Sertraline in Depressed Patients With or at Risk for Coronary Heart Disease | Wrong intervention (omega-3) |
| Somatic and sociodemographic predictors of depression outcome among depressed patients with coronary artery disease-A secondary analysis of the SPIRR-CAD study | Wrong intervention (psychotherapy) |
| Red yeast rice as an adjunct to sertraline for treatment of depression in patients with percutaneous coronary intervention: placebo-controlled trial | Wrong intervention (red yeast rice) |
| Randomized controlled trial of a well-being intervention in cardiac patients | Wrong intervention (well-being sessions) |
| Open-Label Non-Randomized versus Double-Blind Randomized Antidepressive Treatment: What are the Advantages of Clinical Decision over Randomization? | Wrong population (depressed patients; not just depressed patients with coronary disease) |
| Pain Prevention and Treatment Through the Enhancement of the Anti-nociceptive Component of Pain Modulation Profiles | Wrong population (Exclusion Criteria: Presence of diagnosed psychiatric disorders) and outcomes |
| Cardiovascular, cerebrovascular, and hepatic safety of desvenlafaxine for 1 year in women with vasomotor symptoms associated with menopause | Wrong population (Healthy postmenopausal women seeking treatment of vasomotor symptoms) |
| Different Endocrine Effects of an Evening Dose of Amitriptyline, Escitalopram, and Placebo in Healthy Participants | Wrong population (healthy volunteers) |
| Effect of single-dose sertraline on the hypothalamus-pituitary-adrenal system, autonomic nervous system, and platelet function | Wrong population (healthy young men) |
| Bupropion and paroxetine differentially influence cardiovascular and neuroendocrine responses to stress in depressed patients | Wrong population (Medically healthy) |
| [Clinical management of patients with depression and cardiac disease] | Wrong publication type (background article) |
| Does inflammation link clinical depression and coronary artery disease? | Wrong publication type (book) |
| The Canadian cardiac randomized evaluation of antidepressant and psychotherapy efficacy (CREATE) trial | Wrong publication type (book; comments on Lespérance) |
| A 75-year-old man with depression | Wrong publication type (case report and review) |
| Alleviating Effect of Antidepressant Treatment on Psychiatric Symptoms and Cardiac Conditions in a Patient with Coronary Slow Flow Comorbid with Depression and Anxiety | Wrong publication type (case report) |
| [Serious QT interval prolongation associated with velafaxine administration] | Wrong publication type (case report) |
| When prevention is a bad idea: Problems with the DECARD trial and the premise behind it | Wrong publication type (comment on an excluded study) |
| "Effects of citalopram and interpersonal psychotherapy on depression in patients with coronary artery disease": Comment | Wrong publication type (comment to Lespérance) |
| Medicine-based evidence: the case of antidepressants in patients with coronary artery disease | Wrong publication type (comment/editorial about an excluded study) |
| Correction: effect of escitalopram vs placebo treatment for depression on long-term cardiac outcomes in patients with acute coronary syndrome: a randomized clinical trial (JAMA - Journal of the American Medical Association (2018) 320: 4 (350-358) DOI: 10.1001/jama.2018.9422) | Wrong publication type (correction; refers to Kim) |
| the MASTER study | Wrong publication type (registry), ongoing and wrong outcomes |
| "Effects of citalopram and interpersonal psychotherapy on depression in patients with coronary artery disease": Reply | Wrong publication type (reply to Lespérance) |
| Framingham coronary score in individuals with symptoms or diagnoses of mental disorders: A review and meta-analysis | Wrong publication type (review; no new relevant studies) |
| Recent developments in antidepressant therapy in special populations | Wrong publication type (review; no relevant studies) |
| Efficacy and acceptability of antidepressants in patients with ischemic heart disease: systematic review and meta-analysis | Wrong publication type (systematic review; relevant studies already included) |
| Antidepressant drugs and cardiovascular pathology: a clinical overview of effectiveness and safety | Wrong publication type (systematic review; relevant studies already included) |
| Pharmacological interventions for people with depression and chronic physical health problems: Systematic review and meta-analyses of safety and efficacy | Wrong publication type (systematic review; relevant studies already included) |
| Comparative cardiovascular safety of selective serotonin reuptake inhibitors (SSRIs) among Chinese senile depression patients: A network meta-analysis of randomized controlled trials | Wrong publication type (systematic review; no relevant studies) |
| Treatment of anxiety in patients with coronary heart disease: A systematic review | Wrong publication type (systematic review; no relevant studies; one study with escitalopram already assessed and excluded) |
| The effect of selective serotonin reuptake inhibitors on major adverse cardiovascular events: a meta-analysis of randomized-controlled studies in depression | Wrong publication type (systematic review; relevant studies already included) |
| Gateways to clinical trials | Wrong publication type (we could not retrieve the article; however, it is not the type of publication that would report the results of a clinical trial) |
| gateways to clinical trials | Wrong publication type (we could not retrieve the article; however, it is not the type of publication that would report the results of a clinical trial) |
| Venlafaxine treatment stimulates blood platelet activity | Wrong publication type (letter to the editor) |
| Current use of selective serotonin reuptake inhibitors and risk of acute myocardial infarction | Wrong study design (case-control study) |
| Selective Serotonin Reuptake Inhibitors and Serotonin-Norepinephrine Reuptake Inhibitors Are Not Associated With Bleeding or Transfusion in Cardiac Surgical Patients | Wrong study design (case-control study) |
| Prognosis of patients taking selective serotonin reuptake inhibitors before coronary artery bypass grafting | Wrong study design (cohort) |
| Association of baseline anxiety with depression persistence at 6 months in patients with acute cardiac illness | Wrong study design (exploratory analysis of a collaborative care trial) |
| Methylation of the glucocorticoid receptor gene associated with depression in patients with acute coronary syndrome | Wrong study design (exploratory analysis of Kim) |
| Interactions between pro-inflammatory cytokines and statins on depression in patients with acute coronary syndrome | Wrong study design (exploratory analysis of Kim) |
| Gender differences in psychosocial outcomes of psychotherapy trial in patients with depression and coronary artery disease | Wrong study design (exploratory analysis of an excluded study) |
| Modifying effects of depression on the association between BDNF methylation and prognosis of acute coronary syndrome | Wrong study design (exploratory analysis; refers to Kim) |
| Association between Obsessive-Compulsive Symptoms and Long-Term Cardiac Outcomes in Patients with Acute Coronary Syndrome: Effects of Depression Comorbidity and Treatment | Wrong study design (exploratory analysis; refers to Kim) |
| Long-term cardiac outcomes of depression screening, diagnosis and treatment in patients with acute coronary syndrome: the DEPACS study | Wrong study design (follow up analysis of a prospective cohort with a nested RCT; refers to Kim) |
| The risk for a first acute coronary syndrome in patients treated with different types of antidepressants: A population based nested case-control study | Wrong study design (nested case-control study) |
| Antidepressants and the Risk of Cardiovascular Events in Elderly Affected by Cardiovascular Disease: A Real-Life Investigation From Italy | Wrong study design (nested case-control study) |
| The association of obesity and coronary artery disease genes with response to SSRIs treatment in major depression | Wrong study design (observational cohort) |
| Impact of Suicidal Ideation on Long-Term Cardiac Outcomes in Patients with Acute Coronary Syndrome: sex-Specific Differences | Wrong study design (observational cohort), not stratified by participation in RCT |
| Antidepressant Use by Class: Association with Major Adverse Cardiac Events in Patients with Coronary Artery Disease | Wrong study design (retrospective cohort study) |
| Use of serotonin reuptake inhibitors is not associated with increased bleeding after CABG | Wrong study design (retrospective cohort study) |
| Longitudinal associations between sleep problems and mortality in acute coronary syndrome | Wrong study design (we could not retrieve the study, but exploratory analysis; refers to Kim) |
| Hypersensitivity Myocarditis and Necrotizing Coronary Vasculitis by Clomipramine Causing Steroid-Sensitive Cardiogenic Shock. | Wrong study type (case report) |
| Selective serotonin reuptake inhibitors, venlafaxine and duloxetine are associated with in hospital morbidity but not bleeding or late mortality after coronary artery bypass graft surgery | Wrong study type (cohort) |

# Supplemental Material 5 – Main Characteristics of Included Studies

Expanded from Table 1

| Author/ year | Country | Sample size (%men) | Median / mean age (years) | Study population | Definition of microalbuminuria | Outcome assessment | Outcome measures RR/HR (95 % CI) | Follow-up (days) | Adjustments of covariates |
| --- | --- | --- | --- | --- | --- | --- | --- | --- | --- |
| Berton et al. 2001 | Italy | 432 (70 %) | 64.02 ±4.21 | MI | UACR ≥ 30 mg/g | All-cause mortality  CV mortality | 3.7 (2-7) | 365 | Age, DM, history of angina, CK-MB, killip class, heart rate, thrombolysis, LVEF |
| Berton et al. 2004 | Italy | 121 (56 %) | 68.9 ± 10.9 | MI and DM | UACR ≥ 30 ug/mg | All-cause mortality | 2.2 (1.7-2.9) | 1095 | Sex, BMI, current smoking, pre-existing hypertension, prior MI, anterior site of MI |
| Lekastas et al. 2005 | Greece | 223 (77%) | 61.3± 11.5 | MI | AER 20-200 ug/min | All-cause mortality | 10.5(1.25-88.19) | 26 | Sex, hypertension, smoking, previous CAD, use of thrombolysis |
| Solomon et.al 2007 | United States, Canada, Italy | 2977 (72 %) | 66.2 ± 8.1 | Chronic stable coronary disease, LVEF > 40 %, age ≥ 50 | UACR 25-354 ug/mg in women and 17-250 ug/mg in men | All-cause mortality  CV mortality | All-cause mortality : 0.49 (0.35-0.68)  CV mortality : 3.71 (2.18-6.3) | 1752 | Age, sex, history of MI, DM, LVEF < 50 %, current smoking, eGFR, BMI |
| Barton et al. 2010 | Italy | 220 (64%) | 64.02 ± 4.21 | MI | UACR > 20.5 mg/g | All-cause mortality | Short term : 3.9 (2-9)  Longterm : 1.8 (1.1-3.1) | Short term : 515 days  Long term : 3650 days | Age, HT, DM, pre-hospital time delay, CK-MB, heart failure, creatinine clearance,thrombolysis, LVEF |
| Taskiran et al. 2010 | Denmark | 151 (86 %) | 65 (63-68) | MI | UACR > 0.65 mg/mmol | All-cause mortality | 1.71 (1.03-2.83) | 3650 | Age, gender |
| Karki et al. 2014 | Nepal | 134 (66.4%) | 63.9 | ACS | UACR : 30-300 mg/g | All-cause mortality | 1.34 (0.07-24.85) | 365 | Age, gender |
| Kunimura et al. 2015 | Japan | 698 (80%) | 72 ± 9 | Underwent elective PCI | UACR : 30-300 mg/g | CV mortality | 2.56 (1.23-5.32) | 1564 | Age, sex, BMI, LVEF, current smoker, DM, HT, dyslipidemia, eGFR < 60 ml/min/1.73 m^2^ |

*RR* risk ratio; *HR* hazard ratio; *CI* confidence intervals; *UACR* urinary albumin-to-creatinine ratio; *CV* cardiovascular; *DM* diabetes mellitus; *CK*-*MB* creatinine kinase-MB; *LVEF* left ventricular ejection fraction; *BMI* body mass index; *MI* myocardial infarction; *AER* albumin excretion rate; *CAD* coronary artery disease; *eGFR* estimated glomerular filtration rate; *HT* hypertension; *PCI* percutanous coronary intervention

# Supplemental Material 6 – Full Characteristics of the Studies Included

| Study | Publication year | Study type | Study size (n_randomised) | Location | Blinding | Randomisation Method | Allocation concealment | Start Date | End date | Follow-up time | Funding/Conflicts of interest | Ethical considerations |
| --- | --- | --- | --- | --- | --- | --- | --- | --- | --- | --- | --- | --- |
| Strik | 2000 | RCT | 54 | Maastricht, Netherlands | Double-blind | Not reported | Not reported | May 1994 | December 1997 (end of recruitment) | 25 weeks  (9wks acute treatment + 16wks continuation) | Eli Lilly, the Dutch Prevention Fund, and the Maastricht University Hospital Research Fund | Approved by two ethics committees.  Written informed consent was obtained. |
| McFarlane | 2001 | RCT | 38 (only 27 included in the analysis) | Hamilton, Canada | Double-blind | Not specified. Stratification before randomization for those on β-blocker therapy | Not specified | September 1996 | March 1999 (end of recruitment) | 6 months | Peer-reviewed grant from the Heart and Stroke Foundation of Ontario. | Approved by the Institutional Review Board of the Faculty of Health Sciences of McMaster University.  All patients gave written informed consent. |
| Glassman  SADHART | 2002 | RCT | 369 | 7 countries in US, Europe, Canada, and Australia | Double-blind during the RCT | Not specified  Randomization was stratified by LVEF (<30% or ≥30%) and by the presence of 2 depression severity criteria (≥2 prior episodes of depression and a current HAM-D scale30 score ≥18) | Not specified. | April 1997 | April 2001 | 24 weeks | Supported by Pfizer Inc, with additional support from the Suzanne C. Murphy Foundation, Thomas and Caroline Royster Research Fund, and the Perry and Martin Granoff Family Foundation.  FU funded by grant R01-HL081131 from the National Heart, Lung, and Blood Institute of the National Institutes of Health; by the National Alliance for Research in Schizophrenia and Depression; by the Suzanne C. Murphy Foundation; and by the Thomas and Caroline Royster Research Fund.  Possible financial conflicts of interest with: Pfizer, GlaxoSmithKline, Eli Lilly, SanofiSynthelabo, Forest Pharmaceuticals, Astra Merck, AstraZeneca, Aventis, Bristol-Myers Squibb, Cardiometrics, CV Therapeutics, Genentech, Guidant, Hoechst Marion, Roussel, Hoffmann-LaRoche, Knoll AG, Knoll Pharmaceutical, Lilly, Medtronic, Merck, Monarch, Novartis, Novoste, Otsuka American Pharmaceutical, Pfizer Labs, Pratt, qmed, Roche, Sankyo, Sanofi, Searle, Sigma Tau, and Wyeth-Ayerst. | The protocol was approved by each of the 50 individual institutional review boards in the United States and ethics boards in Europe, Canada, and Australia.  Patients provided written informed consent.  To ensure patient safety, an independent data and safety monitoring board provided study oversight. |
|  | 2009 | FU | 359 |  |  |  |  | April 1997 - December 1999. | spring 2005 - summer 2007 | 6.7 years |  |  |
| Mohapatra | 2005 | RCT | 17 | Cuttack, India | Single? | Not specified  The patients were randomized before being evaluated and before consent was sought. The randomization was done by the cardiologist and one psychiatrist (GK), and the rater (PM) remained blind to the status of the participants. | Not specified | Not specified | Not specified | 6 months | In part by Quality of Life Research and Development Foundation. | Consent was obtained |
| Kennedy  (registry results only) | 2006 | RCT | 19 (initial objective 290) | Denmark, Estonia, and Norway | double-blind | Not specified | Not specified | 26 October 2004 | 15 August 2005 | 88 days: 4 completed 24 weeks; 15 stopped early | H. Lundbeck A/S | Conducted in compliance with the principles of *Good Clinical Practice*.  Written informed consent was obtained |
| Lespérance  CREATE | 2007 | RCT | 284 | Canada | double-blind for the medication portion of the trial | Participants underwent 2 separate randomizations: IPT plus clinical management vs clinical management only; and citalopram vs matching placebo.  Randomizations were stratified by therapist using blocks of 4 for the randomization to active medication vs placebo and a single block of 5. followed by randomly permuted blocks of 2. 4. 6, and 8, for randomization to IPT vs clinical management. | The allocation sequences were computer generated and concealed in sequentially numbered, site-specific, sealed opaque envelopes stored at the coordinating centre until randomization. | May 1. 2002 | March 20. 2006 | 12 weeks | Supported by the Canadian Institutes of Health Research (CIHR) Clinical Trials Program grant MCT50397, the Fondation du Centre Hospitalier de l’Universite´ de Montre´ al, and the Fondation de l’Institut de Cardiologie de Montre´ al.  Citalopram and matching placebo were donated by Lundbeck Canada Inc.  None of the sponsors participated in the design, conduct, management, analysis, or interpretation of the data or was involved in the preparation, review, or approval of the manuscript.  Possible financial conflicts of interest with: GlaxoSmithKline, Lundbeck, Wyeth, IsodisNatura, Servier, Solvay, Tromsdorff, Eli Lilly. | Informed consent was obtained |
| Kim  EsDEPACS | 2014 | RCT | 300 (217 included in the analysis) | South Korea | Double-blind | Computer-generated randomization codes | Not specified. | May 2007 | March 2013 | 24 weeks | Unrestricted research grant from H. Lundbeck A/S; grants from the Korea Health 21 R&D, Ministry of Health and Welfare, Republic of Korea (HI12C0003 and A050174); the Basic Science Research Program through the National Research Foundation of Korea (NRF) funded by the Ministry of Science, ICT and Future Planning (NRF-2013R1A2A2A01067367). Escitalopram and placebo were provided by H. Lundbeck A/S.  The funders had no further role in paper publication design; in the collection, analysis, and interpretation of data; in the writing of the report; or in the decision to submit the paper for publication.  Possible financial conflicts of interest with: Lundbeck; Eli Lilly; Otsuka; Pfizer; Johnson & Johnson; Roche; Janssen | Written informed consent was obtained  Approved by the Chonnam National University Hospital Institutional Review Board. |
|  | 2018 | FU | 300 |  | At the end of the RCT, patients were unblinded.  An independent end-point committee adjudicated all potential events and was blinded to the participants’ randomization status. |  |  | May 2007 | June 2017 | 8.1 Years |  |  |
| Tian | 2016 | RCT | 67 | Shandong, China | Double-blind?  One of the groups was not given some patients were not given SSRIs nor placebo | Not specified | Not specified | Not specified | Not specified | 8 weeks | Tackle Key Problems in Science and Technology Program of Shandong Province (grant no 2012YD18051) | Written informed consent was obtained  Approved by the Human Subject and Medical Ethics Committee of Qian Fo Shan Hospital of Shandong University.  Conducted in accordance with the 1964 Declaration of Helsinki and its later amendments |

| Study | Summary of the inclusion criteria | Summary of the exclusion criteria | Primary outcome | Relevant outcomes |
| --- | --- | --- | --- | --- |
| Strik | 18-75 y.o.  3 to 12 months after 1st MI (diagnosed by a cardiologist: clinical picture typical of MI; ECG changes specific for MI; maximum plasma concentration of ASAT of 2x the UNR (80 U/L))  SCL-90 Depression Scale ≥ 23 (men); 28 (women); DSM-III-R criteria for major depression; HAMD-17 score >17 | Psychotic symptoms; another psychiatric diagnosis; history of mania, current pregnancy or lactation, life-threatening noncardiac physical illness, concurrent use of psychotropic drugs (except oxazepam up to 50 mg/d), hypersensitivity to fluoxetine, and liver or severe kidney dysfunction (creatinine clearance <10 ml/min); right ventricular filling pressure >30 mmHg and a low systolic volume or an ATVI <20 cm. | HAMD-17  SCL-90 Hostility Scale | Mortality; Hospitalization; Chest Pain |
| McFarlane | Acute MI  IDD > 15 (before hospital discharge and 2 weeks later) | Predischarge 24-hour Holter: Afib or ventricular ectopic beats > 100/h  Overt CHF  Any life-threatening comorbid condition  Inability to complete the questionnaire  Already on antidepressant medications | HR variability | Mortality |
| Glassman  SADHART | Male or female adults  Acute MI (at least 1 criterion from each of the following 2 categories: Category 1 criteria were: (a) creatine kinase isoenzyme MB (CK-MB) level greater than the upper limit of normal; (b) CK or troponin T or troponin I level more than 2 times the upper limit of normal; or (c) a total lactate dehydrogenase (LDH) level more than 1.5 times the upper limit of normal (with LDH 1 greater than LDH 2); Category 2 criteria were: (a) typical ischemic symptoms (chest pain or shortness of breath) lasting for more than 10 minutes; or (b) electrocardiographic (ECG) evidence of ischemic ST-segment depression, ST-segment elevation, or new pathological Q waves) or to have been hospitalized for unstable angina ((a) experienced angina or anginal equivalent symptoms at rest, with episodes lasting for at least 10 minutes and leading to hospitalization, and had ECG documentation of transient ST-segment elevation or depression of more than 0.5 mm, or had T-wave inversion of greater than 1 mm within 12 hours of an episode of chest pain; or (b) were hospitalized for symptoms of unstable angina and had known coronary artery disease with a documented history of a prior MI, had undergone a prior revascularization procedure, or had documented coronary artery stenosis greater than 75% in one of the major epicardial vessels) in the past 30 days  BDI ≥ 10; Current episode of MDD based on DSM-IV criteria. | Uncontrolled hypertension (sBP >180 mmHg or dBP >100 mmHg); Cardiac surgery anticipated during the next 6 months; Index MI or unstable angina developed less than 3 months after CABG procedure; Resting HR of less than 40/min (or <50/min if symptomatic or daytime sinus pauses of >3.5 seconds); MI or unstable angina of nonatherosclerotic etiology (eg, anemia, cocaine use, periprocedural); Killip class III or IV status; Persistent clinically significant laboratory abnormalities; Significant renal dysfunction, hepatic dysfunction, or other significant noncardiac disease; Women of childbearing potential not using adequate contraception; Current use of class I antiarrhythmic medications, reserpine, guanethidine, clonidine, methyldopa; anticonvulsants or neuroleptics; antidepressants; or regular benzodiazepine.; Initiation of psychotherapy in the 3 months prior to study entry.; Alcohol or substance abuse or dependence in past 6 months; Psychotic symptoms, history of psychosis, bipolar disorder, organic brain syndrome, dementia (or a Mini-Mental State Examination29 score <23); Significant suicide risk | LVEF | Mortality; MI; Stroke; Angina; MACE |
|  |  |  | Mortality | Mortality |
| Mohapatra | Acute MI  Depression diagnosis (DSM IV) | Patients recovering from by-pass surgery  Patients with a history of depressive disorders before the onset of cardiac problem  Current substance harmful use or dependence  Patients already receiving psychotropic medications | HDRS  Cardiac events | Recurrent angina; MI; Cardiac rehospitalizations; Mortality |
| Kennedy  (registry results only) | Able to read and understand the patient information sheet.  Signed the informed consent form.  Male or female outpatient aged 40-75 years (extremes included)  SCL-90-R Depression subscale >=20  Admission for chest pain (or other MI symptom) with a diagnosis of evolving MI not less than 3 weeks and not more than 24 weeks prior to screening, as evidenced by either a or b: (a) Elevation of biochemical markers of myocardial infarction (troponin, CK/MB); (b) ECG changes that are unequivocally consistent with an acute, evolving MI, i.e. development of significant Q-wave in at least two continuous leads.  Healthy physical examination, medical history, ECG, and the results of blood biochemistry and haematology tests other than what is part of the myocardial infarction and its sequalae. | Coronary artery bypass graft (CABG) or percutaneous transluminal coronary angioplasty (PTCA) within 3 weeks of screening or CABG or PTCA during the duration of the study; Known Class IV Canadian Cardiovascular Society (CCS) Classification for angina rating; Known Class IV Congestive Heart Failure Classification of the New York Heart Association (NYHA) rating; Ongoing myocardial ischemia (ST-segment elevation or ST-segment depression) on screening; Cardiac arrhythmias (except atrial fibrillation) necessitating other anti-arrhythmic treatment than beta-blockers or calcium-channel blockers; Uncontrolled high blood pressure: sBP >= 180 mmHg or dBP>= 100 mmHg; Bipolar I and II disorders, major depressive episode with psychotic features or evidence of substance abuse or dependency during the previous 12 months; MADRS total score >= 40; Serious suicide risk (investigator judgment or SCL-90-R > 3 on item 15 or MADRS >=5 on item 10; Use of IMAO or RIMA within 2 weeks prior to screening; Fluoxetine within 5 weeks and other SSRIs or TCAs or SNRIs within the past 2 weeks prior to screening; psychoactive Herbal remedies, (including St-John’s Wort, S-adenosylmethionine or SAMe, kava kava, valerian, ginkgo biloba) within 2 weeks prior to screening; Tryptophan within 2 weeks prior to screening; Any drug used for the augmentation of antidepressant action within 2 weeks prior to screening; Any other anti-depressants within the 2 weeks prior to screening; Mood stabilisers/antimanic drugs/anticonsulvants (e.g. lithium, lamotrigine, valproic acid, gabapentine, carbamazepine, phenytoin) within the 2 weeks prior to screening; Oral antipsychotics in the 2 weeks or depot anti-psychotics in the 6 months prior to screening;·Electroconvulsive therapy (ECT) within the 6 months prior to screening.; Dopamine antagonists (e.g. metoclopramide) for any indication within 2 weeks prior to screening. Serotonergic agonists (e.g. triptans) within 2 weeks prior to screening; Any other drugs with potential psychotropic effects within 2 weeks prior to screening; Formal behaviour therapy or systematic psychotherapy within 4 weeks prior to screening or was planning to initiate such therapy during the study; A serious illness or serious sequelae thereof, including liver or renal insufficiency, or a pulmonary, gastrointestinal, endocrine, neurological, infectious, neoplastic, or metabolic disturbance; Laboratory values outside the normal ranges and considered by the investigator to be clinically significant; Female patients of child-bearing potential: Pregnant or breast-feeding; Lack of adequate contraception (one of the following methods is acceptable: oral / systemic, surgical sterilisation, intra-uterine device (IUD), diaphragm in combination with spermicide or condom in combination with spermicide or vasectomy); Positive pregnancy test at screening; Patients, who have a disease or take medication that, in the opinion of the investigator, could interfere with the assessments of safety, tolerability or efficacy of escitalopram; Lack of response to a previous trial of citalopram or escitalopram; Known drug intolerance or hypersensitivity to citalopram or escitalopram; Participation in other clinical studies (including medications and medical devices) within 30 days prior to screening; Unable or unwilling to comply with the study requirements; History of severe drug allergy or hypersensitivity. | SCL-90-R DS | Mortality |
| Lespérance  CREATE | >18 y.o.  Current major depression diagnosis (DSM IV)  Depressed for 4 weeks or longer  HAM-D >= 20  Established CAD (hospital chart evidence of a previous acute MI or cardiac revascularization or coronary angiography showing >= 50% blockage in at least 1 major coronary artery)  Randomization could not occur less than 1 week following discharge for a cardiac hospitalization, and patients had to have stable CAD based on clinical judgment (eg, no worsening of angina or congestive heart failure symptoms in the past week). | Depression due to a general medical condition (based on clinical judgment); bipolar disorder or major; depression with psychotic features, substance abuse or dependency during the previous 12 months, serious suicide risk, current use of antidepressants, lithium, or anticonvulsants for mood disorder, current treatment with any form of psychotherapy, previous absence of response to citalopram or IPT, 2 or more previous unsuccessful treatments for the index depression episode, lifetime history of early termination (8 weeks) of citalopram or 2 other SSRIs because of adverse events, Mini-Mental State Examination score of less than 24. and clinician judgment that the patient would not adhere to the study regimen.  Patients with coronary artery bypass graft surgery planned during the next 4 months, those with a Canadian Cardiovascular Society Angina Class of 4 (severe limitations),  Participation in other trials  Unable to speak English or French. | HAM-D-24 | MI; CHF; Angina; Stroke; MACE |
| Kim  EsDEPACS | 18~85 yo  Confirmed ACS (ST-segment elevation MI was determined by >30 min of continuous chest pain, a new ST-segment elevation ≥2 mm on at least two contiguous electrocardiographic leads, and creatine kinase-MB more than three times normal; non-ST-segment elevation MI was diagnosed by chest pain and a positive cardiac biochemical marker without new ST-segment elevation; unstable angina was determined by chest pain within the preceding 72 h with or without ST-T wave changes or positive cardiac biochemical markers)  BDI>10; Major or minor depressive disorder (DSM-IV)  Ability to complete study questionnaires; Ability to understand the study objectives and sign informed consent | Occurrence of ACS while hospitalized for another reason; ACS developing less than 3 months after a coronary artery bypass graft procedure; uncontrolled hypertension (sBP >180mmHg or dBP >100mmHg); resting heart rate <40/min; severe physical illnesses threatening life or interfering with the recovery from ACS; persistent clinically significant laboratory abnormalities; concomitant use of class I antiarrhythmic medications, reserpine, guanethidine, clonidine, methyldopa, lithium, anticonvulsants, antipsychotics, or antidepressants; history of neuropsychiatric illnesses such as dementia, Parkinson’s disease, brain tumour, psychosis, bipolar disorder, alcoholism, or other substance dependence; pregnancy; participating in other drug trials | HDRS score | Angina; MI; Stroke |
|  |  |  | MACE | MACE; Mortality; Cardiac Mortality; MI; PCI |
| Tian | Acute MI (ischemic chest pain for>30 minutes but <24 hours, persistent ST-segment elevation >0.1 mV, ST-segment depression or T-wave inversion in two adjacent electrocardiography leads; and significantly elevated blood levels of biomarkers for myocardial injury (creatine kinase-MB and troponin I))  Depression based on HAMD-17 score ≥17 and Self-rating Depression Scale (SDS) | > 85 y. o.  Infection, allergic disorder, endocrine disease, malignancy, autoimmune disease, rheumatic heart disease, severe liver disease, renal failure, history of drug abuse, an anti-inflammatory or immunosuppressant drug except aspirin in the past 3 weeks prior to MI.  No history of heart disease prior to AMI, judged by no diagnosis of atrial fibrillation, myocarditis, endocarditis, valvular heart disease, or requirement of an implanted pacemaker. | HR variability, cardiac function, GRK2 levels | Angina; MI |

| Study | Number of withdrawals and lost to follow-up (and reasons) | Intervention (n_patients) | | Control (n_patients) | | Compliance/Protocol Violation | Time from the latest cardiac event to antidepressant | Type of analysis | Other |
| --- | --- | --- | --- | --- | --- | --- | --- | --- | --- |
| Strik | Acute treatment period dropouts: 2 fluoxetine (1 loss to follow-up; 1 other); 5 placebo (1 intervention ineffective; 2 loss to follow-up; 2 other). Continuation phase dropouts: 3 fluoxetine (2 intervention ineffective; 1 other); 4 placebo (2 intervention ineffective; 2 other) | Fluoxetine 20 mg/d (could be increased to 40 mg/d in week 3 and to 60 mg/d in week 6 depending on the clinical response: the dose was increased if there was a <50% decrease in the HAMD-17 score)  At week 9, patients who wished to continue participating in the trial were given the same dose of fluoxetine for another 16 weeks regardless of their response.  The mean fluoxetine dose was 47.3 mg/d (SD 5 19.1) | 27 | Placebo (same dosage rules as intervention) | 27 | All patients taking fluoxetine had a plasma level of fluoxetine >4 mcg/L on repeated measurements. | Range 3 to 12 months after MI | Assumed mITT  12 dropped out (no reasons reported) – not included  2 excluded (according to pre-established exclusion criteria)  14 dropped out – included; unclear if LOCF or end of trial information was available for these patients | Some patients participated in a routine cardiological rehabilitation program, consisting of physical therapy and psychological education, that lasted up to 3 months after the MI. Patients who were re-hospitalized were dropped from the study and offered additional antidepressant treatment |
| McFarlane | 11 dropped out (6 sertraline and 5 placebo), 3 because of drug side effects, 7 because of noncompliance, and 1 because of frequent ventricular ectopy. | Sertraline 50 mg | 12 | Placebo | 15 | 11 dropped out | Not reported | Per-protocol  11 dropped out – not included | The patients who dropped out were not included in the analysis |
| Glassman  SADHART | Sertraline: 53 Discontinued Trial (16 Adverse Events; 2 Deaths; 5 Lack of Efficacy; 17 Withdrew Consent; 4 Protocol Violation; 6 Lost to Follow-up; 3 Moved Away);  Placebo: 46 Discontinued Trial (11 Adverse Events; 5 Deaths; 6 Lack of Efficacy; 11 Withdrew Consent; 7 Protocol Violation; 4 Lost to Follow-up; 2 Moved Away)  These patients were included in the analysis | Sertraline 50mg/d for the first 6 weeks; could be increased to 100 mg/d at the end of week 6; to 150 mg/d at week 10; and to 200 mg/d at week 12. based on clinical response and tolerability.  If adverse events occurred, the dosage could be reduced by 50 mg at a time, as long as a minimum daily dose of 50 mg was maintained. 50mg tablets were used. Mean final dose was 68.8 mg | 186 | Placebo (same dosage rules as intervention)  Mean final dose was 70.5 mg | 183 | Compliance was checked using pill counts. Not reported | Mean 33.7 (SD 9.9) days | ITT (LOCF for patients who discontinued prematurely) | No concomitant psychiatric medication was allowed apart from chloral hydrate, which could be used intermittently for sleep. |
|  | Vital status was obtained on 361 of 369 (97.8%). |  | 183 |  | 176 |  |  | mITT (patients with missing outcome data were excluded) |  |
| Mohapatra | There were no dropouts due to side effects or non-response. All patients included in the final analysis | Sertraline was titrated to a range of 50 to 200 mg per day depending upon clinical evaluation. | 11 | Treatment as usual | 6 | Not reported | Not reported | Per-protocol?  Patients in the control group, who received antidepressant medication during the study were excluded from the study with LOCF. However, it is not clear how many, if any, patients were in fact excluded. | Patients in 'treatment as usual' (TAU) group who received antidepressant medication during the study from their cardiologists or other sources, were excluded from the study with the last observation carried forward. |
| Kennedy  (registry results only) | 13 patients withdrawn because the study was terminated due to insufficient patient enrolment. 2 patients in the escitalopram group withdrew from the study due to AEs (insomnia and hypersensitivity | Escitalopram 10mg/day for the first 8 weeks  Weeks 9 to 24: the dose was flexible (10 or 20mg/day), adjusted according to the patient’s response to treatment, as judged by the investigator. | 9 | Placebo | 10 | 13 patients withdrawn | Range 3-24 weeks | ITT  Ended early; all patients included in the analysis | Terminated early due to insufficient patient enrolment.  Due to the small number of patients enrolled, no conclusions regarding efficacy could be drawn. |
| Lespérance  CREATE | The number of patients stopping because of intolerance did not differ between the citalopram and placebo groups  Citalopram NO IPT: 10 Discontinued Treatment: 7 Discontinued Medication Only (6 Medication Intolerance; 1 Elevated Liver Enzymes); 3 Discontinued Clinical Management and Medication (1 Medication Intolerance; 1 Brain Tumour; 1 Lost to Follow-up)  Placebo NO IPT: 20 Discontinued Treatment: 14 Discontinued Medication Only (4 Medication Intolerance; 9 Lack of Efficacy; 1 Withdrew); 6 Discontinued Clinical Management and Medication (1 Medication Intolerance; 3 Lack of Efficacy; 1 Wanted IPT; 1 Worsening Depression, Dyspnoea)  Citalopram+ IPT: 8 Discontinued: 6 Discontinued Medication Only (5 Medication Intolerance; 1 Pulmonary Fibrosis); 1 Discontinued IPT Only (No Longer Depressed); 1 Discontinued IPT and Medication (Abdominal Pain)  Placebo + IPT: 16 Discontinued Treatment: 7 Discontinued Medication Only (2 Medication Intolerance; 3 Lack of Efficacy; 1 Unstable Angina; 1 Biopsy Scheduled); 1 Discontinued IPT Only (Found IPT Too Upsetting); 8 Discontinued IPT and Medication (3 Medication Intolerance; 1 Lack of Efficacy; 2 Worsening Depression, Dyspnoea; 1 Lost to Follow-up; 1 Withdrew) | Clinical management only + citalopram (10 mg/d for 1 week and then increased to 20 mg/d. If the centralized 24-item HAM-D score at 6 weeks was not 8 or lower, the dose was increased to a maximum of 40 mg/d. If tolerated, this dose was maintained through the end of the trial).  Given in 20mg tablets. | 75 | Clinical management only + placebo (same dosage rules as intervention) | 67 | All 284 randomized patients received at least 1 dose of study medication and attended at least 1 clinical management or 1 IPT session.  54 patients discontinued 1 or both allocated treatments  during the study. | Median 18.9 months (Range 3 weeks to 31 years) | ITT  94% completed the last HAM-D assessment with LOCF. It is not explicit if all patients completed the serious adverse events analyses, or only 94%. | New prescriptions of benzodiazepines were not permitted. Short-term use of diphenhydramine or zopiclone was allowed for major sleep difficulties. |
|  |  | IPT + clinical management + citalopram (same dosage as above) | 67 | IPT + clinical management + placebo (same dosage as intervention) | 75 |  |  |  |  |
|  |  | the mean citalopram dose at the last visit was 33.1 mg (SD, 10.82 mg) | | The mean final placebo dose was 34.2 mg; (SD, 9.91 mg; P=.38). | |  |  |  |  |
| Kim  EsDEPACS | Escitalopram: 41 exited after baseline evaluation (not included in the analysis); 30 discontinued trial (included in the analysis): 19 lost to follow-up, 6 withdrew consent, 3 lack of efficacy, 2 adverse events.  Placebo: 42 exited after baseline evaluation (not included in the analysis); 30 discontinued trial (included in the analysis): 16 lost to follow-up, 7 withdrew consent, 3 lack of efficacy, 1 adverse event, 3 protocol violation | Escitalopram - 10mg/d initially and could be changed (from 5mg/d to 20mg/d)  Drugs are taken once daily per orally within 30 min after the supper meal.  Mean doses at the last visit were 7.6 (SD 3.7) mg | 149 (108 included in the analysis) | Placebo (same dosage as intervention)  Mean doses at the last visit 8.5 (SD 3.9) mg | 151 (109 included in the analysis) | 83 (28%) exited the study after baseline.  Adherence to medications was ≥75% in 100 of 108 people (93%) receiving escitalopram and in 103 of 109 people (95%) receiving placebo. | 2–14 weeks after an ACS. Mean 29.0 (SD 24.1) days. | mITT | In the recruiting process, higher participation rates were noted in patients with more severe depressive symptoms, resulting in a frequency of major depressive disorder of 57.0%(85/149) among the escitalopram group and 55.6% (84/151) among the placebo group, compared with 22.6%(33/146) in patients who met inclusion criteria but were not randomized |
|  | All patients (including the ones who exited the RCT) completed the follow-up |  | 149 |  | 151 |  |  | ITT | Pre-planned analysis |
| Tian | Not explicitly reported. All patients included in the final analysis. | Paroxetine/Fluoxetine - 10 mg/day initially and increased to 20 mg/day within 1 week based on individual patient response | 23+23 | No antidepressant | 21 | Not reported. | Not explicitly reported. Assumed 1-2 days | ITT |  |
| ITT – intention to treat; mITT – modified intention to treat; LOCF – last observations carried forward; In case of the ITT analysis being the same as the per-protocol analysis, we classified it as ITT. | | | | | | | | | |

# Supplemental Material 7 – Baseline Characteristics of Patients in the Studies Included

Given that there were very few differences in the number of patients included in the Glassman RCT and the Glassman FU, we only extracted baseline characteristics of the Glassman RCT.

| Study | | Demographic Data | | | | Depression | | | | | | | | | | | | | | | |
| --- | --- | --- | --- | --- | --- | --- | --- | --- | --- | --- | --- | --- | --- | --- | --- | --- | --- | --- | --- | --- | --- |
|  |  | Age | | Gender (male) | | MDD (DSM diagnosis) | | mDD^a^ (DSM diagnosis) | | HAMD 17 score^b^ | | HAM-D-24 score | | IDD score | | BDI-II score^c^ | | Prior episodes of depression | | Prior psychotropic treatment | |
|  |  | SSRI | C | SSRI | C | SSRI | C | SSRI | C | SSRI | C | SSRI | C | SSRI | C | SSRI | C | SSRI | C | SSRI | C |
| Strik | | 54.1 (11.3) | 58.7 (10.1) | 78% | 63% | 100% | | 0% | | 22.0 (3.5) | 21.2 (3.7) |  |  |  |  |  |  |  |  |  |  |
| McFarlane | | 56 (11) | 56 (12) | 67% | 53% |  |  |  |  |  |  |  |  | 21 (6.7) | 23 (6.4) |  |  |  |  |  |  |
| Glassman  SADHART | | 56.8 (11.1) | 57.6 (10.4) | 63% | 64% | 100% | | 0% | | 19.6 (5.3) | 19.6 (5.4) |  |  |  |  |  |  | 52% | 50% | 35% | 34% |
| Mohapatra | | 55.2 (12.1) | 56.8 (10.8) | 54.5%^d^ | 66.7% | 100% | | 0% | | 15.0 (5.33) | 16.67 (7.06) |  |  |  |  |  |  |  |  |  |  |
| Kennedy | | 61 (44-75) | | 56% | |  |  |  |  |  |  |  |  |  |  |  |  |  |  |  |  |
| Lespérance  CREATE | No IPT | 57.3 (7.83) | 57.3 (8.95) | 90.7% | 71.6% | 100% | | 0% | |  |  | 29.6 (6.43) | 30.3 (7.64) |  |  | 30.4 (9.27) | 31.3 (9.34) | 45.3% | 40.3% |  |  |
|  | IPT | 58.6 (10.44) | 59.4 (9.28) | 61.2% | 76% | 100% | | 0% | |  |  | 28.8 (6.39) | 30.0 (6.43) |  |  | 30.2 (8.85) | 29.4 (9.83) | 49.3% | 56.0% |  |  |
| Kim  EsDEPACS | RCT | 60.1 (10.9) | 58.5 (10.6) | 62.0% | 57.8% | 56.5% | 55.0% | 43.5% | 45.0% | 15.9 (4.9) | 15.1 (4.3) |  |  |  |  | 19.1 (8.6) | 19.2 (7.5) | 5.6% | 4.6% |  |  |
|  | FU | 60.0 (11.2) | 60.1 (10.5) | 59.1% | 61.6% | 57.0% | 55.6% | 43.0% | 44.4% | 15.9^e^ | 15.6^e^ |  |  |  |  | 18.8 (8.3) | 19.2 (7.7) | 4.0% | 4.6% |  |  |
| Tian | | 62.6 | 62.3 | 54.3% | 57.1% |  |  |  |  | 27.3 (4.5) | 27.1 (4.7) |  |  |  |  |  |  |  |  |  |  |
| Values for all characteristics not reported as percentages are means (standard deviation) or means (range). Values reported in percentages refer to the percentage of patients in the study arm with those characteristics.  a All but Kim RCT were not explicitly reported, but calculated  b Mohapatra does not specify in the text which HAMD scale was used, but cites an article that describes the original HAMD, which is the 17 items HAMD  c Kim does not specify in the text which version of the BDI was used, but cites an article that describes the BDI-II score  d The study presents a table with conflicting data in terms of the number of patients and the respective percentage reported. We chose to calculate the percentage from the number of patients.  e Calculated  SD – Standard deviation  C - Control | | | | | | | | | | | | | | | | | | | | | |

| Study | | Cardiac Status at Baseline | | | | | | | | Treatment of ACS | | | | | |
| --- | --- | --- | --- | --- | --- | --- | --- | --- | --- | --- | --- | --- | --- | --- | --- |
|  |  | MI | | UA^a^ | | Killip Class >1 | | LVEF (mean %) | | PCI | | Lysis | | CABG | |
|  |  | SSRI | C | SSRI | C | SSRI | C | SSRI | C | SSRI | C | SSRI | C | SSRI | C |
| Strik | | 100% | | 0% | |  |  | 51.3% | 50.7% |  |  |  |  |  |  |
| McFarlane | | 100% | | 0% | |  |  | 55% | 58% | 8% | 13% | 42% | 32% | 0% | 0% |
| Glassman SADHART | | 81% | 78% | 19% | 22% | 7.1% | 7.1% | 54% | 52% |  |  |  |  |  |  |
| Mohapatra | | 100% | | 0% | |  |  |  |  |  |  |  |  |  |  |
| Kennedy | | 100% | | 0% | |  |  |  |  |  |  |  |  |  |  |
| Lespérance  CREATE | No IPT | Stable CAD | | | |  |  |  |  | Stable CAD | | | | | |
|  | IPT |  |  |  |  |  |  |  |  |  |  |  |  |  |  |
| Kim  EsDEPACS | RCT | 61.1% | 61.5% | 38.9% | 38.5% | 13.9% | 20.2% | 60.1% | 62.4% |  |  |  |  |  |  |
|  | FU | 61.7% | 60.9% | 38.3% | 39.1% | 16.1% | 23.2% | 60.4% | 61.9% |  |  |  |  |  |  |
| Tian | | 100% | | 0% | |  |  |  |  | 95%^b^ | 91%^b^ |  |  |  |  |
| Values reported in percentages refer to the percentage of patients in the study arm with those characteristics, apart from LVEF.  a All but Glassman RCT and Kim RCT were not explicitly reported, but calculated  b PCI+stent  MI – Myocardial Infarction  UA – Unstable angina  LVEF – Left ventricle ejection fraction  PCI – percutaneous coronary interventions  CABG – Coronary artery bypass graft  C - Control | | | | | | | | | | | | | | | |

| Study | | CV Risk Factors | | | | | | | | | | | | | | | | | | | |
| --- | --- | --- | --- | --- | --- | --- | --- | --- | --- | --- | --- | --- | --- | --- | --- | --- | --- | --- | --- | --- | --- |
|  |  | Previous MI | | Prior revascularizations | | BMI | | Obesity^a^ | | Diabetes^a^ | | Smoker^a^ | | Hypertension^a^ | | Dyslipidemia^a^ | | History of CHF^a^ | | Family History  of CAD^a^ | |
|  |  | SSRI | C | SSRI | C | SSRI | C | SSRI | C | SSRI | C | SSRI | C | SSRI | C | SSRI | C | SSRI | C | SSRI | C |
| Strik | |  |  |  |  |  |  |  |  |  |  |  |  |  |  |  |  |  |  |  |  |
| McFarlane | | 10% | 7% |  |  | 25.6 | 27.2 |  |  | 30% | 23% | 58% | 67% | 50% | 38% |  |  |  |  |  |  |
| Glassman  SADHART | | 43% | 41% | 43% | 42% | 29.8 (men); 29.7 (women) | 28.8 (men); 30.5 (women) | 36% | 30% | 31% | 30% | 27%^b^ | 28%^b^ | 61% | 69% | 70% | 67% | 12% | 16% |  |  |
| Mohapatra | |  |  |  |  |  |  |  |  | 45.5% | 16.7% | 27.3%^c^ | 50%^c^ | 36.4% | 66.7% |  |  |  |  | 45.5% | 0% |
| Kennedy | |  |  |  |  |  |  |  |  |  |  |  |  |  |  |  |  |  |  |  |  |
| Lespérance  CREATE | No IPT | 65.3% | 61.2% | 65.3%; 49.3%^d^ | 54.7%; 46.3%^d^ |  |  | 53.3% | 38.8% | 24.0%^e^ | 25.4%^e^ | 22.7%^b^ | 29.9%^b^ | 66.7%^f^ | 74.6%^f^ |  |  |  |  |  |  |
|  | IPT | 59.7% | 72% | 56.7%; 43.3%^d^ | 53.3%; 42.7%^d^ |  |  | 39.4% | 46.7% | 17.9%^e^ | 22.7%^e^ | 19.4%^b^ | 23.0%^b^ | 70.1%^f^ | 64.0%^f^ |  |  |  |  |  |  |
| Kim  EsDEPACS | RCT | 5.6%^g^ | 7.3%^g^ |  |  |  |  | 38.0% | 50.0% | 31.5% | 30.3% | 33.3%^b^ | 25.7%^b^ | 58.3% | 59.6% | 47.2% | 45.9% |  |  | 4.6%^h^ | 3.7%^h^ |
|  | FU | 5.4%^g^ | 7.3%^g^ |  |  |  |  | 39.6% | 43.0% | 29.5% | 27.2% | 28.9%^b^ | 27.8%^b^ | 60.4% | 62.3% | 49.0% | 47.0% |  |  | 6.0%^h^ | 5.3%^h^ |
| Tian^i^ | |  |  |  |  |  |  | 27% | 24% | 26% | 29% | 50% | 50% | 57% | 54% |  |  |  |  | 45% | 50% |
| BMI reported as means. Values reported in percentages refer to the percentage of patients in the study arm with those characteristics.  a We cannot guaranty that the diagnosis criteria used were the same, nor that they were the ones currently used  b Current smoker  c History of smoking  d Previous angioplasty; Previous CABG surgery  e Medication for diabetes  f History of treatment for hypertension  g Previous ACS  h Family History of ACS  i The study presents a table with some conflicting data in terms of the number of patients and the respective percentage reported. We chose to calculate the percentage from the number of patients.  MI – Myocardial Infarction  C - Control  CHF – Congestive Heart Failure  CAD – Coronary Artery Disease | | | | | | | | | | | | | | | | | | | | | |

| Study | | Concomitant Cardiovascular Drugs | | | | | | | | | | | | | | | | | |
| --- | --- | --- | --- | --- | --- | --- | --- | --- | --- | --- | --- | --- | --- | --- | --- | --- | --- | --- | --- |
|  |  | Aspirin | | Other antiplatelet | | Anticoagulants | | Statins | | ACEi | | β-Blockers | | CCB | | Nitrates | | Diuretic | |
|  |  | SSRI | C | SSRI | C | SSRI | C | SSRI | C | SSRI | C | SSRI | C | SSRI | C | SSRI | C | SSRI | C |
| Strik | | 77.8% | |  | | 11.% | | 40.7%^a^ | | 29.6% | | 61.1%+9.3%^b^ | | 27.8% | | 42.6% | |  | |
| McFarlane | |  |  |  |  |  |  |  |  | 75% | 53% | 60% | 65% | 17% | 7% |  |  |  |  |
| Glassman  SADHART | | 91% | 86% | 19% | 14% | 30% | 28% | 85% | 86% | 53% | 56% | 78% | 85% | 33% | 36% | 66% | 64% | 32% | 45% |
| Mohapatra | |  |  |  |  |  |  |  |  |  |  |  |  |  |  |  |  |  |  |
| Kennedy | |  |  |  |  |  |  |  |  |  |  |  |  |  |  |  |  |  |  |
| Lespérance  CREATE | No IPT | 77.3% | 83.6% | 25.3% | 29.9% |  |  | 84.0% | 95.5% | 50.7% | 53.7% | 64.0% | 67.2% | 37.3% | 23.9% | 9.3% | 11.9% | 21.3% | 25.4% |
|  | IPT | 83.6% | 76.0% | 23.9% | 20.0% |  |  | 82.1% | 86.7% | 58.2% | 52.0% | 68.7% | 73.3% | 19.4% | 22.7% | 13.4% | 17.3% | 26.9% | 22.7% |
| Kim  EsDEPACS | RCT | 91.7% | 89.9% | 76.9% | 72.5% |  |  | 81.5% | 77.1% | 33.3%; 53.7%^c^ | 39.4%; 53.2%^c^ | 71.3% | 73.4% | 35.2% | 45.0% |  |  | 80.6% | 81.7% |
|  | FU |  |  |  |  |  |  |  |  |  |  |  |  |  |  |  |  |  |  |
| Tian | | 100% | 98% | 91% | 100% |  |  | 100% | 100% | 29% | 32% | 76% | 76% | 24% | 28% | 76% | 76% |  |  |
| a Cholesterol-lowering medication  b lipophilic b-blockers + hydrophilic b-blockers  c ACEi; ARA  C - Control  ACEi – Angiotensin conversion enzyme inhibitor  ARA – Angiotensin II receptor antagonist  CCB – Calcium channel blockers | | | | | | | | | | | | | | | | | | | |

| Study | Other Information |
| --- | --- |
| Strik | There were no statistically significant differences between the fluoxetine and placebo groups in age, gender, severity of depression, severity of hostility at baseline (HAMD-17 and SCL-90 hostility scores), or severity of MI (maximum ASAT). There were no differences in specific drugs between groups. No patients were taking antidepressants or antipsychotic drugs before the study. |
| McFarlane | Baseline demographic and clinical characteristics were similar. There were no significant group differences for risk factors, ejection fraction, cardiac medications, revascularization procedures, thrombolysis, or history of previous MI or IDD baseline scores. |
| Glassman  SADHART | There were no significant differences in any baseline demographic or clinical variables. Most patients were in their 50s or 60s and had at least 2 cardiovascular risk factors. Approximately 40% of the patients had experienced a previous MI and about half had experienced at least 1 previous major depression. Depression severity was in the mild to moderate range. The mean number of concomitant medications was 11. Mostly Caucasian (74% vs 79%). |
| Mohapatra | The groups were comparable including age, HDRS scores, and associated risk factors, except that 45% of patients in the intervention group receiving antidepressants had family history of CAD, compared to none in the control group. |
| Kennedy | There were no clinically relevant differences in age, sex, weight, BMI, medical history, or the use of concomitant medication between the treatment groups. All the patients were Caucasian, except one. |
| Lespérance  CREATE | The groups were well-balanced. The only significant difference involved a lower proportion of women randomized to clinical management alone than to IPT. The duration of the index depression episode was more than 6 months in more than 60% of the sample. Comorbid anxiety disorder (24.0% vs 17.9%; 26.9% vs 26.7%). Most had a history of myocardial infarction or at least 1 revascularization procedure. Almost half of the participants had had previous depression, and one quarter had a comorbid anxiety disorder. |
| Kim  EsDEPACS | There were no significant differences between the 2 groups in any characteristic (age; gender; education; HDRS score; MADRS score; BDI score; CGI-S score; Previous depression; DSM-IV diagnosis (Major depressive disorder vs Minor depressive disorder); Hypertension; Diabetes mellitus; Hypercholesterolemia; Obesity; Current smoker; Previous ACS; Family history of ACS; current ACS diagnosis (myocardial infarction; unstable angina); Killip class; LVEF; Troponin I; Creatine kinase-MB). 61.3 of patients had Myocardial infarction and severity of ACS was relatively mild (81% Killip class 1 and 89% NYHA class I). The serum CK-MB level was significantly higher in patients exiting after baseline evaluation compared to those followed up (P=.043) |
| Tian | There were no significant differences in the ratio of sex, average age, common risk factors for cardio­vascular diseases, data from laboratory assays, or drugs from concomitant therapies. |

# Supplemental Material 8 – Assumptions and Data Treatment for the Meta-analysis

**Assumptions and data treatment regarding the inclusion of studies for the quantitative analyses, merger of arms, and subdivision into sub-studies**

All studies that explicitly reported any of the relevant outcomes were included for the qualitative analysis, however, only studies with non-null event rates were included in the primary metanalysis for each outcome; studies with null event rates were included in some of the exploratory analyses. In the case of the same study being reported as an RCT and as a follow-up after an RCT, both results were extracted, but only one of them was included in each analysis to avoid repetition of patients (in the primary analyses for each outcome, the data used was the one pooled from the FU, due to its longer follow-up time). In the case of multiple interventions within a trial, we included all comparisons between groups that only differed in SSRI use as separate sub-studies. In the case of multiple arms using SSRIs compared with a control arm, the multiple SSRI arms were grouped into one, so as not to duplicate the data of the control group.

In the Lespérance et al (CREATE) trial^32^, the participants were randomized twice, first, between interpersonal psychotherapy (IPT) and no interpersonal psychotherapy, and then in each of those groups, between citalopram and placebo. We included both the No IPT + citalopram vs No IPT + placebo and the IPT+citalopram vs ITP+Placebo comparisons as separate sub-studies instead of grouping them in only one Citalopram and Placebo comparison, in order not to have homogenous comparison groups. Analyses removing the IPT comparison were carried out in order to ensure that that extra intervention did not affect our results due to possible synergetic or redundant effects of the 2 interventions administered. The choice to include the IPT+citalopram vs ITP+Placebo comparison and to separate the CREATE study into 2 sub-studies did not significantly affect the results of our analyses nor the conclusions drawn.

In the Tian et al trial^34^, there were 3 groups being compared: paroxetine, fluoxetine, and no antidepressant treatment. We merged the paroxetine and fluoxetine groups and compared them to the no antidepressant treatment group. This assumption did not substantially affect the results of our analyses nor the conclusions drawn.

**Assumptions and data treatment regarding outcome data**

For the outcomes other than mortality, some articles reported the number of patients with events (Glassman RCT, Lespérance, and Kim RCT) whereas others reported the number of events (Strik, Mohapatra, and Tian). It is unclear if the number of events and patients with events is the same in the studies that reported the number of events (even though the follow-up time was short, and that Mohapatra and Tian report those results also as percentages, we cannot guaranty that the number of events and patients with events was the same). Kim FU refers that patients could have more than 1 event; all patients were followed up to the present evaluation point or until death, and that nonhierarchical end-point analyses were used.

We chose not to make any distinction between the studies that reported the outcomes differently in this regard and used the data as provided.

In the studies that had losses to follow-up/discontinuations, but included those patients in the analyses (Strik^27^, Glassman RCT^29^, Glassman FU^14^, Lespérance^32^, Kim RCT^33^, and possibly Mohapatra^30^), we used the outcome data as reported, thus assuming, for statistical purposes, that no-one that was lost to follow-up/discontinued had relevant events after they exited the study, even though we cannot guaranty that that was the case.

McFarlane^28^ reported a per-protocol analysis, with 11 patients excluded from the trial. We made no assumption of no mortality for the excluded patients, even though none of the reasons for exclusion was death; and used the outcome data as reported.

No assumption of null mortality was made in the articles that did not explicitly report mortality, even when all patients were accounted for at the end of the trial or none of the discontinuation reasons was mortality. This assumption did not substantially affect the results of our analyses nor the conclusions drawn, given that we only included studies with non-null mortality in the primary metanalysis for mortality.

Glassman RCT^29^ reported that 2 cerebrovascular events occurred in patients in the placebo group and were not included in the reported outcome data for stroke, given that they occurred in hospitalized patients. We decided to use the data as reported and not add those 2 events to the stroke tally. This decision did not substantially affect the results of our analyses nor the conclusions drawn.

Glassman RCT^29^ refers that the composite endpoints were death or urgent rehospitalization for MI, congestive heart failure, stroke, or angina. Given that the article does not refer the location of the deaths, we took a conservative approach and withdrew the number of deaths to the composite endpoint to get the number of hospitalizations. This assumption did not substantially affect the results of our analyses nor the conclusions drawn.

In the Lespérance et al (CREATE) trial^32^. we did not assume the total number of patients with cardiovascular serious adverse events to be equivalent to the number of hospitalizations.

Strik referred that one of the reasons for rehospitalization for a cardiac event was anaemia, even though anaemia is not, *per se*, a cardiac event. Despite Lespérance classifying anaemia as a non-cardiovascular event, we chose not to withdraw the anaemia rehospitalization from the total number of rehospitalization for a cardiac event in the data pooled from Strik, given that we did not know the specific circumstances of that hospitalization. This assumption did not significantly affect the results of our analyses nor the conclusions drawn.

We assumed that “chest pain” reported in Strik as cardiac chest pain, i.e. angina, even though we cannot guaranty that none of the cases were regarding non-cardiac chest pain.

The studies that reported angina (Strik^27^, Glassman RCT^29^, Mohapatra^30^, Lespérance^32^, Kim RCT^33^, Tian^34^), didn’t specify whether the data reported was regarding stable angina or unstable angina. We did not make any assumptions about the type of angina reported and analysed all the data jointly.

GlassmanFU refers that they were able to obtain the vital status of 361 of the 369 patients of the SADHART trial and that 75 patients had died during a median follow-up of 6.7 years. However, the only place in the article that reports mortality stratified by intervention/placebo allocation is a table that only includes patients whose CGI-I scores were available (359 patients). Given that the number of deaths was 75 and that that table reports rates of 37/176 and 38/183 deaths for the intervention and placebo groups respectively, and that the article also informs us that they retrieved the vital status of 177 and 184 patients in the intervention and placebo groups respectively, it is possible to extrapolate that the mortality rate including all 361 patients was 37/177 and 38/184. However, given that that is not specifically reported, we chose to use the 37/176 and 38/183 rates. This decision did not significantly affect the results of our analyses nor the conclusions drawn.

KimFU refers that the rate of myocardial infarction rate was 13/149 and 23/151 for the escitalopram and placebo groups, respectively. However, another article (Kim J-M et al (2020). Long term cardiac outcomes of depression screening, diagnosis, and treatment in patients with acute coronary syndrome: the DEPACS study. Psychological Medicine 1–11. https:// doi.org/10.1017/S003329171900388X) that reports on the same study refers that the rate was 12/149 and 12/151 respectively and provides the results also in percentages 8.1% and 15.2% (which do not correspond with the 12/149 and 12/151 outcome rates). We chose to use the results reported in the article specific for the EsDEPACS extended follow up (the included article for KimFU: JAMA. 2018;320(4):350-357. doi:10.1001/jama.2018.9422).

No other assumptions or data treatment were carried out.

These decisions were made before any statistical analysis was carried out, and posthumous statistical analyses (not reported) were carried out that proved that they did not significantly affect the results of our analyses nor the conclusions drawn. We did not carry out statistical analyses regarding the KimFU 12/149 and 12/151 MI rates, given that we assumed those rates were mistakes.

# Supplemental Material 9 – Relevant Outcomes Extracted from the Studies Included

| Study | Date | Outcomes (ITT) | Events_SSRI | Total_SSRI | Events_Control | Total_Control |
| --- | --- | --- | --- | --- | --- | --- |
| Strik | 2000 | Mortality | 0 | 27 | 0 | 27 |
|  |  | Hospitalization | 1 | 27 | 6 | 27 |
|  |  | Chest pain | 5 | 27 | 4 | 27 |
| McFarlane | 2001 | Mortality (per protocol) | 0 | 12 | 0 | 15 |
| Glassman RCT | 2002 | Mortality | 2 | 186 | 5 | 183 |
|  |  | MI | 5 | 186 | 7 | 183 |
|  |  | Composite end point* | 32 | 186 | 41 | 183 |
|  |  | Hospitalization** | 30 | 186 | 36 | 183 |
|  |  | Angina | 26 | 186 | 30 | 183 |
|  |  | Stroke*** | 2 | 186 | 2 | 183 |
| Glassman FU | 2009 | Mortality | 38 | 183 | 37 | 176 |
| Mohapatra | 2005 | Mortality | 0 | 11 | 1 | 6 |
|  |  | MI | 2 | 11 | 4 | 6 |
|  |  | Hospitalization | 4 | 11 | 5 | 6 |
|  |  | Angina | 5 | 11 | 4 | 6 |
|  |  | CHF | 0 | 11 | 3 | 6 |
| Kennedy | 2006 | Mortality | 0 | 9 | 0 | 10 |
| Lespérance NO IPT | 2007 | MI | 0 | 75 | 1 | 67 |
|  |  | Angina | 0 | 75 | 1 | 67 |
|  |  | Stroke | 0 | 75 | 0 | 67 |
|  |  | CHF | 1 | 75 | 0 | 67 |
|  |  | Other | 0 | 75 | 0 | 67 |
|  |  | Total**** | 1 | 75 | 2 | 67 |
| Lespérance IPT | 2007 | MI | 0 | 67 | 2 | 75 |
|  |  | Angina | 2 | 67 | 1 | 75 |
|  |  | Stroke | 1 | 67 | 0 | 75 |
|  |  | CHF | 1 | 67 | 1 | 75 |
|  |  | Other | 1 | 67 | 0 | 75 |
|  |  | Total**** | 5 | 67 | 4 | 75 |
| Kim RCT | 2015 | MI | 1 | 108 | 0 | 109 |
|  |  | Angina | 1 | 108 | 4 | 109 |
|  |  | Stroke | 1 | 108 | 1 | 109 |
| Kim FU | 2018 | Mortality | 31 | 149 | 37 | 151 |
|  |  | Cardiovascular mortality | 16 | 149 | 20 | 151 |
|  |  | MI | 13 | 149 | 23 | 151 |
|  |  | PCI | 19 | 149 | 30 | 151 |
|  |  | MACE***** | 61 | 149 | 81 | 151 |
| Tian (merged) | 2016 | MI | 6 | 46 | 4 | 21 |
|  |  | Angina | 13 | 46 | 10 | 21 |
| Tian (paroxetine) |  | MI | 3 | 23 | 4 | 21 |
|  |  | Angina | 7 | 23 | 10 | 21 |
| Tian (fluoxetine) |  | MI | 3 | 23 | 4 | 21 |
|  |  | Angina | 6 | 23 | 10 | 21 |

RCT – Randomized controlled trial; FU – Follow-up analysis; MI – Myocardial infarction; CHF – Congestive heart failure; PCI – Percutaneous coronary intervention; *Death or urgent rehospitalization for MI, congestive heart failure, stroke, or angina; ** The article refers that the composite endpoints were death or urgent rehospitalization for MI, congestive heart failure, stroke, or angina. Given that the article does not refer of the location of the deaths, we withdrew the number of deaths to the composite endpoint to get the number of hospitalizations; *** There were 2 additional cerebrovascular events in the placebo arm (1 stroke and 1 transient ischemic attack) that were not included, because they occurred during an hospitalization but were not the initial reason for the hospitalization; **** If more than one outcome occurred in a patient, the patient was only counted once; ***** Mortality + Cardiovascular mortality + MI +PCI.

# Supplemental Material 10 – RoB 2 Table of Risk of Bias for the Studies Included

The risk of bias was not different among the different outcomes measured (apart from ‘angina’, which is already discussed in the ‘Limitations’ portion of the article). As a result, we report the risk of bias for all outcomes (but for angina) simultaneously.

| Study | Randomization process | Deviations from intended interventions | Missing outcome data | Measurement of the outcome | Selection of the reported result |  | Overall |
| --- | --- | --- | --- | --- | --- | --- | --- |
| Strik |  |  |  |  |  |  |  |
| McFarlane |  |  |  |  |  |  |  |
| Glassman RCT |  |  |  |  |  |  |  |
| Mohapatra |  |  |  |  |  |  |  |
| Kennedy |  |  |  |  |  |  |  |
| Lespérance |  |  |  |  |  |  |  |
| Kim RCT |  |  |  |  |  |  |  |
| Tian |  |  |  |  |  |  |  |
|  |  |  |  |  |  |  |  |
| Glassman FU |  |  |  |  |  |  |  |
| Kim FU |  |  |  |  |  |  |  |

RoB 2^23^ classification of risk of bias for the included studies. Kennedy was downgraded due to insufficient recruitment and early termination. The FUs were downgraded due to being extended follow-up analyses. +: Low risk; ?: Some concerns; -: High risk

# Supplemental Material 11 – Rob2 Assessment of the RCTs Included

The risk of bias was not different among the different outcomes measured (apart from ‘angina’, which is already discussed in the ‘Limitations’ portion of the article). As a result, we report the risk of bias for all outcomes (but for angina) simultaneously.

| Strik – Mortality; Hospitalization; Chest pain | | |
| --- | --- | --- |
| **Domain** | **Signalling question** | **Response** |
| **Bias arising from the randomization process** | 1.1 Was the allocation sequence random? | NI |
|  | 1.2 Was the allocation sequence concealed until participants were enrolled and assigned to interventions? | NI |
|  | 1.3 Did baseline differences between intervention groups suggest a problem with the randomization process? | N |
|  | **Risk of bias judgement** | Some concerns |
| **Bias due to deviations from intended interventions** | 2.1.Were participants aware of their assigned intervention during the trial? | PN |
|  | 2.2.Were carers and people delivering the interventions aware of participants' assigned intervention during the trial? | PN |
|  | 2.3. If Y/PY/NI to 2.1 or 2.2: Were there deviations from the intended intervention that arose because of the experimental context? | NA |
|  | 2.4 If Y/PY to 2.3: Were these deviations likely to have affected the outcome? | NA |
|  | 2.5. If Y/PY/NI to 2.4: Were these deviations from intended intervention balanced between groups? | NA |
|  | 2.6 Was an appropriate analysis used to estimate the effect of assignment to intervention? | PY |
|  | 2.7 If N/PN/NI to 2.6: Was there potential for a substantial impact (on the result) of the failure to analyse participants in the group to which they were randomized? | NA |
|  | **Risk of bias judgement** | Low |
| **Bias due to missing outcome data** | 3.1 Were data for this outcome available for all, or nearly all, participants randomized? | N |
|  | 3.2 If N/PN/NI to 3.1: Is there evidence that result was not biased by missing outcome data? | N |
|  | 3.3 If N/PN to 3.2: Could missingness in the outcome depend on its true value? | PY |
|  | 3.4 If Y/PY/NI to 3.3: Is it likely that missingness in the outcome depended on its true value? | PN |
|  | **Risk of bias judgement** | Some concerns |
| **Bias in measurement of the outcome** | 4.1 Was the method of measuring the outcome inappropriate? | PN |
|  | 4.2 Could measurement or ascertainment of the outcome have differed between intervention groups? | N |
|  | 4.3 Were outcome assessors aware of the intervention received by study participants? | PN |
|  | 4.4 If Y/PY/NI to 4.3: Could assessment of the outcome have been influenced by knowledge of intervention received? | NA |
|  | 4.5 If Y/PY/NI to 4.4: Is it likely that assessment of the outcome was influenced by knowledge of intervention received? | NA |
|  | **Risk of bias judgement** | Low |
| **Bias in selection of the reported result** | 5.1 Were the data that produced this result analysed in accordance with a pre-specified analysis plan that was finalized before unblinded outcome data were available for analysis? | PY |
|  | 5.2 ... multiple eligible outcome measurements (e.g. scales, definitions, time points) within the outcome domain? | N |
|  | 5.3 ... multiple eligible analyses of the data? | N |
|  | **Risk of bias judgement** | Low |
| **Overall bias** | **Risk of bias judgement** | Some concerns |

Relevant commentaries:

1.1 and 1.2 – Only refers ‘patients were randomly assigned’

1.3 There were no statistically significant differences between the fluoxetine and placebo groups in age, gender, severity of depression, severity of hostility at baseline (HAMD-17 and SCL-90 hostility scores), or severity of MI (maximum ASAT). There were no differences in specific drugs between groups

2.1 and 2.2 – ‘randomized, placebo-controlled, double-blind design’; no significant differences in side effects or occurrence of drug-specific side effects.

2.6 – Study refers that ‘Of the 68 depressed patients, 12 dropped out at a later stage, and 2 were excluded because of an ATVI <20 cm and a right ventricular filling pressure >30 mm Hg’. The exclusions were in line with the predefined exclusion criteria. It is unclear why the patients dropped out or if they had already been randomized (although the study also refers the number of randomized patients was 54). 14 patients withdrew from the study but they were included in the analysis. We considered the 12 dropped-out patients to be participants with missing outcome data, and as a result, we considered this analysis this to be an mITT analysis.

3 – The data of the 14 patients that withdrew from the study was imputed using the last observation carried forward technique. No analysis methods that correct for bias, or sensitivity analyses were carried out. It is possible that the withdrawals were related to participants’ health status, but not likely, given that, the reasons for discontinuation were lack of effect, loss to follow-up, and other.

4 – The study doesn’t specify the specific method of measuring the rate of cardiac events, but does refer that ‘adverse effects and events were assessed at baseline and weeks 1, 3, 6, and 9; thereafter these parameters were measured monthly’.

5 – ‘adverse effects and events were assessed’. We extracted the raw number of patients with events that occurred in each arm and calculated the RRs ourselves.

| McFarlane – Mortality | | |
| --- | --- | --- |
| **Domain** | **Signalling question** | **Response** |
| **Bias arising from the randomization process** | 1.1 Was the allocation sequence random? | NI |
|  | 1.2 Was the allocation sequence concealed until participants were enrolled and assigned to interventions? | NI |
|  | 1.3 Did baseline differences between intervention groups suggest a problem with the randomization process? | N |
|  | **Risk of bias judgement** | Some concerns |
| **Bias due to deviations from intended interventions** | 2.1.Were participants aware of their assigned intervention during the trial? | PN |
|  | 2.2.Were carers and people delivering the interventions aware of participants' assigned intervention during the trial? | PN |
|  | 2.3. If Y/PY/NI to 2.1 or 2.2: Were there deviations from the intended intervention that arose because of the experimental context? | NA |
|  | 2.4 If Y/PY to 2.3: Were these deviations likely to have affected the outcome? | NA |
|  | 2.5. If Y/PY/NI to 2.4: Were these deviations from intended intervention balanced between groups? | NA |
|  | 2.6 Was an appropriate analysis used to estimate the effect of assignment to intervention? | N |
|  | 2.7 If N/PN/NI to 2.6: Was there potential for a substantial impact (on the result) of the failure to analyse participants in the group to which they were randomized? | PN |
|  | **Risk of bias judgement** | Some concerns |
| **Bias due to missing outcome data** | 3.1 Were data for this outcome available for all, or nearly all, participants randomized? | N |
|  | 3.2 If N/PN/NI to 3.1: Is there evidence that result was not biased by missing outcome data? | N |
|  | 3.3 If N/PN to 3.2: Could missingness in the outcome depend on its true value? | NI |
|  | 3.4 If Y/PY/NI to 3.3: Is it likely that missingness in the outcome depended on its true value? | PN |
|  | **Risk of bias judgement** | Some concerns |
| **Bias in measurement of the outcome** | 4.1 Was the method of measuring the outcome inappropriate? | N |
|  | 4.2 Could measurement or ascertainment of the outcome have differed between intervention groups? | N |
|  | 4.3 Were outcome assessors aware of the intervention received by study participants? | N |
|  | 4.4 If Y/PY/NI to 4.3: Could assessment of the outcome have been influenced by knowledge of intervention received? | NA |
|  | 4.5 If Y/PY/NI to 4.4: Is it likely that assessment of the outcome was influenced by knowledge of intervention received? | NA |
|  | **Risk of bias judgement** | Low |
| **Bias in selection of the reported result** | 5.1 Were the data that produced this result analysed in accordance with a pre-specified analysis plan that was finalized before unblinded outcome data were available for analysis? | NI |
|  | 5.2 ... multiple eligible outcome measurements (e.g. scales, definitions, time points) within the outcome domain? | N |
|  | 5.3 ... multiple eligible analyses of the data? | N |
|  | **Risk of bias judgement** | Some concerns |
| **Overall bias** | **Risk of bias judgement** | Some concerns |

Relevant commentaries:

1.1 and 1.2 – Only refers ‘randomized, placebo-controlled trial with stratification before randomization for those on β-blocker therapy’

1.3 Baseline demographic and clinical characteristics were similar. There were no significant group differences for risk factors, ejection fraction, cardiac medications, revascularization procedures, thrombolysis, or history of previous MI or IDD baseline scores.

2.1 and 2.2 – ‘double-blind, randomized, placebo-controlled trial’; no reference to the occurrence of substantial side effects.

2.6 – Study refers that ‘Eleven dropped out 3 because of drug side effects, 7 because of noncompliance, and 1 because of frequent ventricular ectopy’. Those patients were not included in the analysis (per-protocol analysis).

3 – Data missing for the patients who dropped out. Reasons for dropping out were recorded: none of them were mortality, there is no reference to their vital status at the end of the trial. Those patients were not included in the analysis (per-protocol analysis).

4 – Mortality – no patients died.

5 – ‘Although it was not the purpose of the study to measure clinical outcomes, the sample size being too small, we note there were no deaths in any of the groups during 6 months of follow-up’.

| Glassman RCT – Mortality; MI; Hospitalization; Angina; Stroke | | |
| --- | --- | --- |
| **Domain** | **Signalling question** | **Response** |
| **Bias arising from the randomization process** | 1.1 Was the allocation sequence random? | Y |
|  | 1.2 Was the allocation sequence concealed until participants were enrolled and assigned to interventions? | PY |
|  | 1.3 Did baseline differences between intervention groups suggest a problem with the randomization process? | N |
|  | **Risk of bias judgement** | Low |
| **Bias due to deviations from intended interventions** | 2.1.Were participants aware of their assigned intervention during the trial? | N |
|  | 2.2.Were carers and people delivering the interventions aware of participants' assigned intervention during the trial? | N |
|  | 2.3. If Y/PY/NI to 2.1 or 2.2: Were there deviations from the intended intervention that arose because of the experimental context? | NA |
|  | 2.4 If Y/PY to 2.3: Were these deviations likely to have affected the outcome? | NA |
|  | 2.5. If Y/PY/NI to 2.4: Were these deviations from intended intervention balanced between groups? | NA |
|  | 2.6 Was an appropriate analysis used to estimate the effect of assignment to intervention? | Y |
|  | 2.7 If N/PN/NI to 2.6: Was there potential for a substantial impact (on the result) of the failure to analyse participants in the group to which they were randomized? | NA |
|  | **Risk of bias judgement** | Low |
| **Bias due to missing outcome data** | 3.1 Were data for this outcome available for all, or nearly all, participants randomized? | PN |
|  | 3.2 If N/PN/NI to 3.1: Is there evidence that result was not biased by missing outcome data? | PN |
|  | 3.3 If N/PN to 3.2: Could missingness in the outcome depend on its true value? | NI |
|  | 3.4 If Y/PY/NI to 3.3: Is it likely that missingness in the outcome depended on its true value? | PN |
|  | **Risk of bias judgement** | Some concerns |
| **Bias in measurement of the outcome** | 4.1 Was the method of measuring the outcome inappropriate? | N |
|  | 4.2 Could measurement or ascertainment of the outcome have differed between intervention groups? | N |
|  | 4.3 Were outcome assessors aware of the intervention received by study participants? | N |
|  | 4.4 If Y/PY/NI to 4.3: Could assessment of the outcome have been influenced by knowledge of intervention received? | NA |
|  | 4.5 If Y/PY/NI to 4.4: Is it likely that assessment of the outcome was influenced by knowledge of intervention received? | NA |
|  | **Risk of bias judgement** | Low |
| **Bias in selection of the reported result** | 5.1 Were the data that produced this result analysed in accordance with a pre-specified analysis plan that was finalized before unblinded outcome data were available for analysis? | Y |
|  | 5.2 ... multiple eligible outcome measurements (e.g. scales, definitions, time points) within the outcome domain? | N |
|  | 5.3 ... multiple eligible analyses of the data? | N |
|  | **Risk of bias judgement** | Low |
| **Overall bias** | **Risk of bias judgement** | Some concerns |

Relevant commentaries:

1.1 and 1.2 – Randomization was stratified by LVEF (<30% or ≥30%) and by the presence of 2 depression severity criteria (≥2 prior episodes of depression and a current HAM-D scale30 score ≥18)

1.3 – There were no significant differences in any baseline demographic or clinical variables.

2.1 and 2.2 – ‘double-blind, randomized, placebo-controlled trial’; ‘physicians [] were blinded’. ‘Nausea and diarrhea were significantly more common in patients taking sertraline’ – however, we did not consider this to significantly compromise blinding.

2.6 – ITT analysis carried out.

3 – Data missing for the patients who discontinued was imputed, using the last assessment carried forward method.

Reasons for discontinuing included Adverse Events, Lack of Efficacy, Withdrawing Consent, and Losses to Follow-up, among others.

4 – ‘The clinical events committee (CEC), based at the DCRI, provided adjudication of all serious adverse events by physicians who were blinded to treatment assignment. The CEC-adjudicated composite endpoints were death or urgent rehospitalization for MI, congestive heart failure, stroke, or angina’.

5 – We extracted the raw number of patients with events that occurred in each arm and calculated the RRs ourselves.

| Mohapatra – Mortality; MI; Hospitalization; Angina; CHF | | |
| --- | --- | --- |
| **Domain** | **Signalling question** | **Response** |
| **Bias arising from the randomization process** | 1.1 Was the allocation sequence random? | NI |
|  | 1.2 Was the allocation sequence concealed until participants were enrolled and assigned to interventions? | NI |
|  | 1.3 Did baseline differences between intervention groups suggest a problem with the randomization process? | PY |
|  | **Risk of bias judgement** | High |
| **Bias due to deviations from intended interventions** | 2.1.Were participants aware of their assigned intervention during the trial? | NI |
|  | 2.2.Were carers and people delivering the interventions aware of participants' assigned intervention during the trial? | PY |
|  | 2.3. If Y/PY/NI to 2.1 or 2.2: Were there deviations from the intended intervention that arose because of the experimental context? | N |
|  | 2.4 If Y/PY to 2.3: Were these deviations likely to have affected the outcome? | NA |
|  | 2.5. If Y/PY/NI to 2.4: Were these deviations from intended intervention balanced between groups? | NA |
|  | 2.6 Was an appropriate analysis used to estimate the effect of assignment to intervention? | PN |
|  | 2.7 If N/PN/NI to 2.6: Was there potential for a substantial impact (on the result) of the failure to analyse participants in the group to which they were randomized? | NI |
|  | **Risk of bias judgement** | High |
| **Bias due to missing outcome data** | 3.1 Were data for this outcome available for all, or nearly all, participants randomized? | NI |
|  | 3.2 If N/PN/NI to 3.1: Is there evidence that result was not biased by missing outcome data? | N |
|  | 3.3 If N/PN to 3.2: Could missingness in the outcome depend on its true value? | NI |
|  | 3.4 If Y/PY/NI to 3.3: Is it likely that missingness in the outcome depended on its true value? | PN |
|  | **Risk of bias judgement** | Some Concerns |
| **Bias in measurement of the outcome** | 4.1 Was the method of measuring the outcome inappropriate? | N |
|  | 4.2 Could measurement or ascertainment of the outcome have differed between intervention groups? | PN |
|  | 4.3 Were outcome assessors aware of the intervention received by study participants? | PY |
|  | 4.4 If Y/PY/NI to 4.3: Could assessment of the outcome have been influenced by knowledge of intervention received? | PN |
|  | 4.5 If Y/PY/NI to 4.4: Is it likely that assessment of the outcome was influenced by knowledge of intervention received? | NA |
|  | **Risk of bias judgement** | Low |
| **Bias in selection of the reported result** | 5.1 Were the data that produced this result analysed in accordance with a pre-specified analysis plan that was finalized before unblinded outcome data were available for analysis? | PY |
|  | 5.2 ... multiple eligible outcome measurements (e.g. scales, definitions, time points) within the outcome domain? | N |
|  | 5.3 ... multiple eligible analyses of the data? | N |
|  | **Risk of bias judgement** | Low |
| **Overall bias** | **Risk of bias judgement** | High |

Relevant commentaries:

1.1 and 1.2 – ‘The patients were randomized as treatment as usual group or intervention group before being evaluated. The process followed a design where participants are randomized to control or intervention before consent is sought. The randomization was done by the cardiologist and one psychiatrist (GK), and the rater (PM) remained blind to the status of the participants’.

1.3 – 45% of patients in the intervention group receiving antidepressants had family history of CAD, compared to none in the tau group’.

2.1 and 2.2 – No placebo; ‘The randomization was done by the cardiologist and one psychiatrist (GK), and the rater (PM) remained blind to the status of the participants’; ‘GK helped in evaluation of patients, monitored treatment, MB helped in collection of cardiological data’.

2.6, 2.7, 3 – ‘Patients in this group [control], who received antidepressant medication during the study from their cardiologists or other sources, were excluded from the study with the last observation carried forward’. No information on how many patients were excluded.

4 – We considered that the rate of occurrence of Mortality; MI; Hospitalization; Angina; or CHF is probably not influenceable by inadequate blinding in a way that would affect the results.

5 – We extracted the raw number of patients with events that occurred in each arm and calculated the RRs ourselves.

| Kennedy – Mortality | | |
| --- | --- | --- |
| **Domain** | **Signalling question** | **Response** |
| **Bias arising from the randomization process** | 1.1 Was the allocation sequence random? | NI |
|  | 1.2 Was the allocation sequence concealed until participants were enrolled and assigned to interventions? | NI |
|  | 1.3 Did baseline differences between intervention groups suggest a problem with the randomization process? | NI |
|  | **Risk of bias judgement** | Some concerns |
| **Bias due to deviations from intended interventions** | 2.1.Were participants aware of their assigned intervention during the trial? | N |
|  | 2.2.Were carers and people delivering the interventions aware of participants' assigned intervention during the trial? | N |
|  | 2.3. If Y/PY/NI to 2.1 or 2.2: Were there deviations from the intended intervention that arose because of the experimental context? | NA |
|  | 2.4 If Y/PY to 2.3: Were these deviations likely to have affected the outcome? | NA |
|  | 2.5. If Y/PY/NI to 2.4: Were these deviations from intended intervention balanced between groups? | NA |
|  | 2.6 Was an appropriate analysis used to estimate the effect of assignment to intervention? | Y |
|  | 2.7 If N/PN/NI to 2.6: Was there potential for a substantial impact (on the result) of the failure to analyse participants in the group to which they were randomized? | NA |
|  | **Risk of bias judgement** | Low |
| **Bias due to missing outcome data** | 3.1 Were data for this outcome available for all, or nearly all, participants randomized? | Y |
|  | 3.2 If N/PN/NI to 3.1: Is there evidence that result was not biased by missing outcome data? | NA |
|  | 3.3 If N/PN to 3.2: Could missingness in the outcome depend on its true value? | NA |
|  | 3.4 If Y/PY/NI to 3.3: Is it likely that missingness in the outcome depended on its true value? | NA |
|  | **Risk of bias judgement** | Low |
| **Bias in measurement of the outcome** | 4.1 Was the method of measuring the outcome inappropriate? | N |
|  | 4.2 Could measurement or ascertainment of the outcome have differed between intervention groups? | N |
|  | 4.3 Were outcome assessors aware of the intervention received by study participants? | N |
|  | 4.4 If Y/PY/NI to 4.3: Could assessment of the outcome have been influenced by knowledge of intervention received? | NA |
|  | 4.5 If Y/PY/NI to 4.4: Is it likely that assessment of the outcome was influenced by knowledge of intervention received? | NA |
|  | **Risk of bias judgement** | Low |
| **Bias in selection of the reported result** | 5.1 Were the data that produced this result analysed in accordance with a pre-specified analysis plan that was finalized before unblinded outcome data were available for analysis? | PY |
|  | 5.2 ... multiple eligible outcome measurements (e.g. scales, definitions, time points) within the outcome domain? | N |
|  | 5.3 ... multiple eligible analyses of the data? | N |
|  | **Risk of bias judgement** | Low |
| **Overall bias** | **Risk of bias judgement** | High |

Relevant commentaries:

Downgraded due to very insufficient enrolment (objective: 290; actual:19) and early termination.

The authors refer: ‘The limited number of enrolled patients resulted in insufficient data for any meaningful analyses’.

| Lespérance – MI; Angina; Stroke; CHF | | |
| --- | --- | --- |
| **Domain** | **Signalling question** | **Response** |
| **Bias arising from the randomization process** | 1.1 Was the allocation sequence random? | Y |
|  | 1.2 Was the allocation sequence concealed until participants were enrolled and assigned to interventions? | Y |
|  | 1.3 Did baseline differences between intervention groups suggest a problem with the randomization process? | N |
|  | **Risk of bias judgement** | Low |
| **Bias due to deviations from intended interventions** | 2.1.Were participants aware of their assigned intervention during the trial? | N |
|  | 2.2.Were carers and people delivering the interventions aware of participants' assigned intervention during the trial? | N |
|  | 2.3. If Y/PY/NI to 2.1 or 2.2: Were there deviations from the intended intervention that arose because of the experimental context? | NA |
|  | 2.4 If Y/PY to 2.3: Were these deviations likely to have affected the outcome? | NA |
|  | 2.5. If Y/PY/NI to 2.4: Were these deviations from intended intervention balanced between groups? | NA |
|  | 2.6 Was an appropriate analysis used to estimate the effect of assignment to intervention? | Y |
|  | 2.7 If N/PN/NI to 2.6: Was there potential for a substantial impact (on the result) of the failure to analyse participants in the group to which they were randomized? | NA |
|  | **Risk of bias judgement** | Low |
| **Bias due to missing outcome data** | 3.1 Were data for this outcome available for all, or nearly all, participants randomized? | Y |
|  | 3.2 If N/PN/NI to 3.1: Is there evidence that result was not biased by missing outcome data? | NA |
|  | 3.3 If N/PN to 3.2: Could missingness in the outcome depend on its true value? | NA |
|  | 3.4 If Y/PY/NI to 3.3: Is it likely that missingness in the outcome depended on its true value? | NA |
|  | **Risk of bias judgement** | Low |
| **Bias in measurement of the outcome** | 4.1 Was the method of measuring the outcome inappropriate? | N |
|  | 4.2 Could measurement or ascertainment of the outcome have differed between intervention groups? | N |
|  | 4.3 Were outcome assessors aware of the intervention received by study participants? | N |
|  | 4.4 If Y/PY/NI to 4.3: Could assessment of the outcome have been influenced by knowledge of intervention received? | NA |
|  | 4.5 If Y/PY/NI to 4.4: Is it likely that assessment of the outcome was influenced by knowledge of intervention received? | NA |
|  | **Risk of bias judgement** | Low |
| **Bias in selection of the reported result** | 5.1 Were the data that produced this result analysed in accordance with a pre-specified analysis plan that was finalized before unblinded outcome data were available for analysis? | Y |
|  | 5.2 ... multiple eligible outcome measurements (e.g. scales, definitions, time points) within the outcome domain? | N |
|  | 5.3 ... multiple eligible analyses of the data? | N |
|  | **Risk of bias judgement** | Low |
| **Overall bias** | **Risk of bias judgement** | Low |

Relevant commentaries:

1.1 and 1.2 – ‘Participants underwent 2 separate randomizations: once to receive IPT plus clinical management vs clinical management only and once to receive citalopram vs matching placebo pill. This resulted in 4 groups: (1) IPT plus clinical management and citalopram; (2) IPT plus clinical management and placebo pill; (3) clinical management only and citalopram; and (4) clinical management only and placebo pill. Randomizations were stratified by therapist using blocks of 4 for the randomization to active medication vs placebo and a single block of 5, followed by randomly permuted blocks of 2, 4, 6, and 8, for randomization to IPT vs clinical management. The allocation sequences were computer generated and concealed in sequentially numbered, site-specific, sealed opaque envelopes stored at the coordinating center until randomization. Prior to the first study appointment, the coordinating center verified all eligibility criteria by telephone and facsimile. After confirming eligibility, the therapist completed the first clinical management session and then telephoned the coordinating center, where the randomization envelope was opened’.

1.3 – ‘The groups were well-balanced’

2.1 and 2.2 – The medication portion of the trial was completed in a double-blind fashion, with all therapists, patients, site psychiatrists, telephone raters, and coordinating center personnel blinded to patients’ group assignment. Code-break cards were provided to site pharmacies’.

2.6 – ‘All analyses were based on the intention-to-treat principle, with the last-observation-carried-forward approach applied for missing data’.

3 – 94% of patients completed the final assessment.

4 – ‘The safety of each treatment was judged according to the occurrence of serious adverse events as defined by US Food and Drug Administration regulations. All serious adverse events were classified as cardiovascular or noncardiovascular by the event committee, blinded to treatment allocation.

5 – We extracted the raw number of patients with events that occurred in each arm and calculated the RRs ourselves.

| Kim RCT – MI; Angina; Stroke | | |
| --- | --- | --- |
| **Domain** | **Signalling question** | **Response** |
| **Bias arising from the randomization process** | 1.1 Was the allocation sequence random? | Y |
|  | 1.2 Was the allocation sequence concealed until participants were enrolled and assigned to interventions? | PY |
|  | 1.3 Did baseline differences between intervention groups suggest a problem with the randomization process? | N |
|  | **Risk of bias judgement** | Low |
| **Bias due to deviations from intended interventions** | 2.1.Were participants aware of their assigned intervention during the trial? | N |
|  | 2.2.Were carers and people delivering the interventions aware of participants' assigned intervention during the trial? | N |
|  | 2.3. If Y/PY/NI to 2.1 or 2.2: Were there deviations from the intended intervention that arose because of the experimental context? | NA |
|  | 2.4 If Y/PY to 2.3: Were these deviations likely to have affected the outcome? | NA |
|  | 2.5. If Y/PY/NI to 2.4: Were these deviations from intended intervention balanced between groups? | NA |
|  | 2.6 Was an appropriate analysis used to estimate the effect of assignment to intervention? | PY |
|  | 2.7 If N/PN/NI to 2.6: Was there potential for a substantial impact (on the result) of the failure to analyse participants in the group to which they were randomized? | NA |
|  | **Risk of bias judgement** | Low |
| **Bias due to missing outcome data** | 3.1 Were data for this outcome available for all, or nearly all, participants randomized? | PN |
|  | 3.2 If N/PN/NI to 3.1: Is there evidence that result was not biased by missing outcome data? | N |
|  | 3.3 If N/PN to 3.2: Could missingness in the outcome depend on its true value? | NI |
|  | 3.4 If Y/PY/NI to 3.3: Is it likely that missingness in the outcome depended on its true value? | PN |
|  | **Risk of bias judgement** | Some concerns |
| **Bias in measurement of the outcome** | 4.1 Was the method of measuring the outcome inappropriate? | N |
|  | 4.2 Could measurement or ascertainment of the outcome have differed between intervention groups? | N |
|  | 4.3 Were outcome assessors aware of the intervention received by study participants? | N |
|  | 4.4 If Y/PY/NI to 4.3: Could assessment of the outcome have been influenced by knowledge of intervention received? | NA |
|  | 4.5 If Y/PY/NI to 4.4: Is it likely that assessment of the outcome was influenced by knowledge of intervention received? | NA |
|  | **Risk of bias judgement** | Low |
| **Bias in selection of the reported result** | 5.1 Were the data that produced this result analysed in accordance with a pre-specified analysis plan that was finalized before unblinded outcome data were available for analysis? | Y |
|  | 5.2 ... multiple eligible outcome measurements (e.g. scales, definitions, time points) within the outcome domain? | N |
|  | 5.3 ... multiple eligible analyses of the data? | N |
|  | **Risk of bias judgement** | Low |
| **Overall bias** | **Risk of bias judgement** | Some concerns |

Relevant commentaries:

1.1 and 1.2 – ‘Computer-generated randomization codes’.

1.3 – ‘There were no significant differences between the 2 groups in any characteristic’

2.1 and 2.2 – Double-blind, placebo-controlled.

2.6 – mITT

3 – 28% of patients without information after baseline; The only difference between followed up patients and not followed up patients was the serum CK-MB level (higher in patients exiting after baseline evaluation P=.043.)

4 – For general safety outcomes, adverse events were recorded at all visits. Serious adverse events, both clinical and laboratory, were assessed. Double-blind

5 – We extracted the raw number of patients with events that occurred in each arm and calculated the RRs ourselves.

| Tian – MI; Angina | | |
| --- | --- | --- |
| **Domain** | **Signalling question** | **Response** |
| **Bias arising from the randomization process** | 1.1 Was the allocation sequence random? | NI |
|  | 1.2 Was the allocation sequence concealed until participants were enrolled and assigned to interventions? | NI |
|  | 1.3 Did baseline differences between intervention groups suggest a problem with the randomization process? | N |
|  | **Risk of bias judgement** | Some concerns |
| **Bias due to deviations from intended interventions** | 2.1.Were participants aware of their assigned intervention during the trial? | NI/PY |
|  | 2.2.Were carers and people delivering the interventions aware of participants' assigned intervention during the trial? | NI/PY |
|  | 2.3. If Y/PY/NI to 2.1 or 2.2: Were there deviations from the intended intervention that arose because of the experimental context? | NI |
|  | 2.4 If Y/PY to 2.3: Were these deviations likely to have affected the outcome? | NA |
|  | 2.5. If Y/PY/NI to 2.4: Were these deviations from intended intervention balanced between groups? | NA |
|  | 2.6 Was an appropriate analysis used to estimate the effect of assignment to intervention? | Y |
|  | 2.7 If N/PN/NI to 2.6: Was there potential for a substantial impact (on the result) of the failure to analyse participants in the group to which they were randomized? | NA |
|  | **Risk of bias judgement** | Some concerns |
| **Bias due to missing outcome data** | 3.1 Were data for this outcome available for all, or nearly all, participants randomized? | Y |
|  | 3.2 If N/PN/NI to 3.1: Is there evidence that result was not biased by missing outcome data? | NA |
|  | 3.3 If N/PN to 3.2: Could missingness in the outcome depend on its true value? | NA |
|  | 3.4 If Y/PY/NI to 3.3: Is it likely that missingness in the outcome depended on its true value? | NA |
|  | **Risk of bias judgement** | Low |
| **Bias in measurement of the outcome** | 4.1 Was the method of measuring the outcome inappropriate? | PN |
|  | 4.2 Could measurement or ascertainment of the outcome have differed between intervention groups? | N |
|  | 4.3 Were outcome assessors aware of the intervention received by study participants? | NI |
|  | 4.4 If Y/PY/NI to 4.3: Could assessment of the outcome have been influenced by knowledge of intervention received? | PN |
|  | 4.5 If Y/PY/NI to 4.4: Is it likely that assessment of the outcome was influenced by knowledge of intervention received? | NA |
|  | **Risk of bias judgement** | Low |
| **Bias in selection of the reported result** | 5.1 Were the data that produced this result analysed in accordance with a pre-specified analysis plan that was finalized before unblinded outcome data were available for analysis? | PY |
|  | 5.2 ... multiple eligible outcome measurements (e.g. scales, definitions, time points) within the outcome domain? | N |
|  | 5.3 ... multiple eligible analyses of the data? | N |
|  | **Risk of bias judgement** | Low |
| **Overall bias** | **Risk of bias judgement** | Some concerns |

Relevant commentaries:

1.1 and 1.2 – The study only refers ‘randomized’.

1.3 – ‘There were no significant differences in the ratio of sex, average age, common risk factors for cardiovascular diseases, data from laboratory assays, or drugs from concomitant therapies’.

2.1 and 2.2 – The study refers “double bind”, but one of the arms did not receive any intervention (probably double-blind between the SSRI groups but not between the no antidepressant group).

2.3 – No information in terms of deviations from interventions.

2.6 – ITT

3 – Data available for all patients. Patients were reassessed using continuous follow-up questionnaire scores and outcome surveys after 8 weeks, including the incidence of postinfarction angina pectoris and recurrent MI.

4 – The method (self-reporting only, or complemented by hospital records, ECG+lab, …) for determining the rates of MI and Angina were not specified. However, for the outcome Myocardial Infarction, we assumed it followed the standards of STEMI and NSTEMI diagnosis and the Universal Definition of Myocardial Infarction, so, the method of measuring the outcome was probably not inappropriate, the measurement or ascertainment of the outcome couldn’t realistically have differed between groups and nor could the assessment of the outcome have been influenced by knowledge of the intervention received. For the angina outcome, it is not known if what was reported was referring to unstable angina or stable angina, a limitation that is already referenced in the main text. Regarding the possible effect in terms of a decrease of angina self-reporting, we concede that it is possible that it may have influenced by knowledge of intervention received, but we do not consider it to be likely. Under those circumstances, we consider that the method of measuring the outcome was probably not inappropriate (N/PN/NI net the same result for question 4.1), the method was similar between groups (a NI answer here would only change the result of the domain result to “some concerns”, but wouldn’t affect the overall rating), it is not explicitly known if the outcome assessors were aware of the allocation (given the probable non-blinding of patients, an answer of Y/PY could have been given to this question, however, it would not affect the domain rating); and given that it is possible that it may have been influenced by knowledge of the intervention received, but we do not consider it to be likely, the domain score would be “some concerns”, but it would not change the overall score (given that it is already “some concerns”).

The study refers “double bind”, but one of the arms did not receive any intervention.

5 – We extracted the raw number of patients with events that occurred in each arm and calculated the RRs ourselves.

Rob 2 statistics of the classification of risk of bias for the RCTs (as a percentage)

# Supplemental Material 12 – Rob2 Assessment of the FUs Included

| Glassman FU – Mortality | | |
| --- | --- | --- |
| **Domain** | **Signalling question** | **Response** |
| **Bias arising from the randomization process** | 1.1 Was the allocation sequence random? | Y |
|  | 1.2 Was the allocation sequence concealed until participants were enrolled and assigned to interventions? | PY |
|  | 1.3 Did baseline differences between intervention groups suggest a problem with the randomization process? | N |
|  | **Risk of bias judgement** | Low |
| **Bias due to deviations from intended interventions** | 2.1.Were participants aware of their assigned intervention during the trial? | PY |
|  | 2.2.Were carers and people delivering the interventions aware of participants' assigned intervention during the trial? | NI |
|  | 2.3. If Y/PY/NI to 2.1 or 2.2: Were there deviations from the intended intervention that arose because of the experimental context? | N |
|  | 2.4 If Y/PY to 2.3: Were these deviations likely to have affected the outcome? | NA |
|  | 2.5. If Y/PY/NI to 2.4: Were these deviations from intended intervention balanced between groups? | NA |
|  | 2.6 Was an appropriate analysis used to estimate the effect of assignment to intervention? | Y |
|  | 2.7 If N/PN/NI to 2.6: Was there potential for a substantial impact (on the result) of the failure to analyse participants in the group to which they were randomized? | NA |
|  | **Risk of bias judgement** | Low |
| **Bias due to missing outcome data** | 3.1 Were data for this outcome available for all, or nearly all, participants randomized? | Y |
|  | 3.2 If N/PN/NI to 3.1: Is there evidence that result was not biased by missing outcome data? | NA |
|  | 3.3 If N/PN to 3.2: Could missingness in the outcome depend on its true value? | NA |
|  | 3.4 If Y/PY/NI to 3.3: Is it likely that missingness in the outcome depended on its true value? | NA |
|  | **Risk of bias judgement** | Low |
| **Bias in measurement of the outcome** | 4.1 Was the method of measuring the outcome inappropriate? | N |
|  | 4.2 Could measurement or ascertainment of the outcome have differed between intervention groups? | N |
|  | 4.3 Were outcome assessors aware of the intervention received by study participants? | NI |
|  | 4.4 If Y/PY/NI to 4.3: Could assessment of the outcome have been influenced by knowledge of intervention received? | N |
|  | 4.5 If Y/PY/NI to 4.4: Is it likely that assessment of the outcome was influenced by knowledge of intervention received? | NA |
|  | **Risk of bias judgement** | Low |
| **Bias in selection of the reported result** | 5.1 Were the data that produced this result analysed in accordance with a pre-specified analysis plan that was finalized before unblinded outcome data were available for analysis? | PY |
|  | 5.2 ... multiple eligible outcome measurements (e.g. scales, definitions, time points) within the outcome domain? | N |
|  | 5.3 ... multiple eligible analyses of the data? | N |
|  | **Risk of bias judgement** | Low |
| **Overall bias** | **Risk of bias judgement** | Some concerns |

Relevant commentaries:

Same as Glassman RCT, apart from:

2.1, 2.2, 4.3 – No information on blinding after the end of the RCT period.

2.3 – The experimental part of the study occurred in a double-blind fashion, and as a result, no deviations from the intended interventions arose because of the experimental context during that phase. We considered that the intervention during the observational part of the study was “treatment as usual provided by their physicians outside of the trial (according to what they considered necessary for each patient)” for both arms. Given this definition, there were also no deviations from the intended interventions. This decision did not affect the overall rating for this study (see Supplemental material 13 – Further discussion of limitations)

3.1 – Contrasting the RCT, in which there were considerable losses to follow-up/discontinuations, in the FU vital status was obtained for 97.8% of randomized patients (361 out of 369 participants) during a median follow-up of 6.7 years.

4.1, 4.2 – Five years after the final patient completed the trial, researchers began to collect the vital status of SADHART participants. Site personnel were asked to provide evidence (office visit, laboratory testing, or documented telephone calls) of the last date on which each participant was known to be alive or the date of death if they knew the patient had died. If evidence that the patient was alive in the previous 18 months was unavailable, US sites were asked to send names and Social Security numbers to Columbia University College of Physicians and Surgeons, where they were combined with sex and birth date to create a database for submission to the National Death Index, Division of Vital Statistics, National Center for Health Statistics, US Department of Health and Human Services.

Overall bias: Downgraded due to the observational nature of the follow-up period

| Kim FU – Mortality; Cardiac Mortality; MI; PCI | | |
| --- | --- | --- |
| **Domain** | **Signalling question** | **Response** |
| **Bias arising from the randomization process** | 1.1 Was the allocation sequence random? | Y |
|  | 1.2 Was the allocation sequence concealed until participants were enrolled and assigned to interventions? | PY |
|  | 1.3 Did baseline differences between intervention groups suggest a problem with the randomization process? | N |
|  | **Risk of bias judgement** | Low |
| **Bias due to deviations from intended interventions** | 2.1.Were participants aware of their assigned intervention during the trial? | PY |
|  | 2.2.Were carers and people delivering the interventions aware of participants' assigned intervention during the trial? | NI |
|  | 2.3. If Y/PY/NI to 2.1 or 2.2: Were there deviations from the intended intervention that arose because of the experimental context? | N |
|  | 2.4 If Y/PY to 2.3: Were these deviations likely to have affected the outcome? | NA |
|  | 2.5. If Y/PY/NI to 2.4: Were these deviations from intended intervention balanced between groups? | NA |
|  | 2.6 Was an appropriate analysis used to estimate the effect of assignment to intervention? | Y |
|  | 2.7 If N/PN/NI to 2.6: Was there potential for a substantial impact (on the result) of the failure to analyse participants in the group to which they were randomized? | NA |
|  | **Risk of bias judgement** | Low |
| **Bias due to missing outcome data** | 3.1 Were data for this outcome available for all, or nearly all, participants randomized? | Y |
|  | 3.2 If N/PN/NI to 3.1: Is there evidence that result was not biased by missing outcome data? | NA |
|  | 3.3 If N/PN to 3.2: Could missingness in the outcome depend on its true value? | NA |
|  | 3.4 If Y/PY/NI to 3.3: Is it likely that missingness in the outcome depended on its true value? | NA |
|  | **Risk of bias judgement** | Low |
| **Bias in measurement of the outcome** | 4.1 Was the method of measuring the outcome inappropriate? | N |
|  | 4.2 Could measurement or ascertainment of the outcome have differed between intervention groups? | N |
|  | 4.3 Were outcome assessors aware of the intervention received by study participants? | N |
|  | 4.4 If Y/PY/NI to 4.3: Could assessment of the outcome have been influenced by knowledge of intervention received? | NA |
|  | 4.5 If Y/PY/NI to 4.4: Is it likely that assessment of the outcome was influenced by knowledge of intervention received? | NA |
|  | **Risk of bias judgement** | Low |
| **Bias in selection of the reported result** | 5.1 Were the data that produced this result analysed in accordance with a pre-specified analysis plan that was finalized before unblinded outcome data were available for analysis? | Y |
|  | 5.2 ... multiple eligible outcome measurements (e.g. scales, definitions, time points) within the outcome domain? | N |
|  | 5.3 ... multiple eligible analyses of the data? | N |
|  | **Risk of bias judgement** | Low |
| **Overall bias** | **Risk of bias judgement** | Some concerns |

Relevant commentaries:

Same as Kim RCT, apart from:

2.1, 2.2, 4.3 – At the end of the RCT patients were unblinded. An independent end-point committee adjudicated all potential events and was blinded to the participants’ randomization status.

2.3 – The experimental part of the study occurred in a double-blind fashion, and as a result, no deviations from the intended interventions arose because of the experimental context during that phase. We considered that the intervention during the observational part of the study was “treatment as usual provided by their physicians outside of the trial (according to what they considered necessary for each patient)” for both arms. Given this definition, there were also no deviations from the intended interventions. This decision did not affect the overall rating for this study (see Supplemental material 13 – Further discussion of limitations)

3.1, 4.1, 4.2 – Contrasting the RCT, in which there were considerable losses to follow-up/discontinuations, in the FU the relevant outcomes were obtained for all patients: Comprehensive evaluations for cardiac outcomes were possible for this study because KAMIR manages and records detailed data electronically on hospital admissions, deaths, recurrent MI, and percutaneous coronary intervention (PCI). Each patient’s status was evaluated and recorded at every hospital visit using the KAMIR protocol administered by KAMIR researchers. At least 2 researchers were exclusively assigned to this study during the entire study period specifically to obtain accurate data on long-term cardiac outcomes. These researchers made telephone contact with patients or their family members the day before each expected hospital visit to facilitate continued participation and to maximize follow-up. For those transferred to other hospitals, the researchers verified the patient’s status through a telephone call to the hospital and using the KAMIR database shared between the hospitals. When reasons for loss to follow-up could not be identified in the hospital records, mainly deaths outside of the hospital (3% of all participants), deaths were confirmed through telephone contact with a family member and through death certification in the National Registration Records, with which it was confirmed that all patients were registered. Emigration did not occur for any cohort member. As described, assertive maximization of outcome information collection was carried out at the Clinical Trial Centre of CNUH, including checks for completeness and accuracy each year after the registration and manual supplementary data collection via records checks and telephone follow-up where indicated. Through these measures, all baseline participants were thus successfully and completely followed up for these outcomes. Because a patient could have more than 1 event, all patients were followed up to the present evaluation point or until death, and non-hierarchical end-point analyses were used. An independent end-point committee composed of study cardiologists adjudicated all potential events and was blinded to the participants’ randomization status.

Overall bias: Downgraded due to the observational nature of the follow-up period

5.3 – The results were calculated in adjusted and non-adjusted HR. This did not influence our extraction of the data, given that we extracted the number of events and calculated the unadjusted RR ourselves.

Rob 2 statistics of the classification of risk of bias for the FUs (as a percentage)

Relevant commentaries:

In contrast to the corresponding RCTs, in which there were considerable losses to follow-up/discontinuations, in the FUs the relevant outcomes extracted were obtained for 97.8% and 100% of patients.

# Supplemental Material 13 – List of the Quantitative Analyses Carried Out

| Quantitative analyses | | Number of Studies | Number of Patients | | RR (95% CI) | I^2^ |  |
| --- | --- | --- | --- | --- | --- | --- | --- |
| **Outcome 1** | **All-cause Mortality** | | | | | | |
| Result 1.1 | Primary analysis (only studies with at least one event were included) | 3 | 676 | | 0.906 (0.678-1.210) | 0% | ns |
| Result 1.2 | Exploratory analysis: Only studies with at least one event were included; high risk of bias studies were excluded; Same analysis as only FU | 2 | 659 | | 0.919 (0.687-1.229) | 0% | ns |
| Result 1.3 | Exploratory analysis: All studies that explicitly reported mortality were included (even the ones where mortality was null) | 6 | 776 | | 0.909 (0.682-1.211) | 0% | ns |
| Result 1.4 | Exploratory analysis: Only RCTs with at least one event were included | 2 | 386 | | 0.337 (0.080-1.418) | 0% | ns |
| Result 1.5 | Exploratory analysis: Only RCTs with at least one event were included and the high bias risk studies were excluded - only 1 study included; metanalysis not carried out | 1 | 369 | | 0.394 (0.077-2.003) |  | ns |
| Result 1.6 | Exploratory analysis: Only RCTs that explicitly reported mortality were included (even the ones where mortality was null) | 5 | 486 | | 0.478 (0.143-1.599) | 0% | ns |
| Result 1.7 | Exploratory analysis: Only RCTs that explicitly reported mortality were included (even the ones where mortality was null); high bias risk studies were excluded. | 3 | 450 | | 0.516 (0.127-2.090) | 0% | ns |
| **Outcome 2** | **Cardiac Mortality** | | | | | | |
| Result 2.1 | Primary analysis – only 1 study included; metanalysis not carried out | 1 | 300 | | 0.811 (0.437-1.503) |  | ns |
| **Outcome 3** | **Myocardial infarction** | | | | | | |
| Result 3.1 | Primary analysis | 6 | 1037 | | 0.541 (0.341-0.859) | 0% | s |
| Result 3.2 | Leave one out (jackknife) analysis of the primary analysis | Statistical significance maintained even if any one of the studies were excluded | | | | | |
| Result 3.3 | Exploratory analysis: All studies included, except for the high bias risk study | 5 | 1020 | | 0.590 (0.362-0.963) | 0% | s |
| Result 3.4 | Exploratory analysis: Only studies that only included post-ACS patients were included | 4 | 753 | | 0.560 (0.350-0.898) | 0% | s |
| Result 3.5 | Exploratory analysis: Only studies that only included post-ACS patients were included; the high bias risk study was excluded | 3 | 736 | | 0.617 (0.373-1.019) | 0% | ns |
| Result 3.6 | Exploratory analysis: Only RCTs were included | 6 | 954 | | 0.549 (0.286-1.051) | 0% | ns |
| Result 3.7 | Exploratory analysis: Only RCTs were included; the high bias risk study was excluded | 5 | 937 | | 0.671 (0.321-1.402) | 0% | ns |
| Result 3.8 | Exploratory analysis: Only RCTs that only included post-ACS patients were included | 4 | 670 | | 0.590 (0.299-1.166) | 0% | ns |
| Result 3.9 | Exploratory analysis: Only RCTs that only included post-ACS patients were included; the high bias risk study was excluded | 3 | 653 | | 0.758 (0.347-1.659) | 0% | ns |
| Result 3.10 | Exploratory analysis: Comparison with psychotherapy in both arms excluded | 5 | 895 | | 0.553 (0.347-0.882) | 0% | s |
| Result 3.11 | Exploratory analysis: Comparison with psychotherapy in both arms excluded; the high bias risk study was excluded | 4 | 878 | | 0.606 (0.369-0.995) | 0% | s |
| Result 3.12 | Exploratory analysis: Only RCTs; comparison with psychotherapy in both arms excluded | 5 | 812 | | 0.573 (0.295-1.115) | 0% | ns |
| Result 3.13 | Exploratory analysis: Only RCTs; comparison with psychotherapy in both arms excluded; the high bias risk study was excluded | 4 | 795 | | 0.719 (0.336-1.538) | 0% | ns |
| **Outcome 4** | **Hospitalizations** | | | | | | |
| Result 4.1 | Primary analysis | 3 | 440 | | 0.570 (0.291-1.118) | 43% | ns |
| Result 4.2 | Exploratory analysis: All studies included, except for the high bias risk study | 2 | 223 | | 0.513 (0.123-2.129) | 55% | ns |
| **Outcome 5** | **Angina** | | | | | | |
| Result 5.1 | Primary analysis | 7 | 1008 | | 0.756 (0.544-1.051) | 0% | ns |
| Result 5.2 | Exploratory analysis: All studies included, except for the high bias risk study | 6 | 991 | | 0.770 (0.539-1.100) | 0% | ns |
| Result 5.3 | Exploratory analysis: Only studies that only included post-ACS patients were included | 5 | 724 | | 0.748 (0.535-1.045) | 0% | ns |
| Result 5.4 | Exploratory analysis: Only studies that only included post-ACS patients were included; the high risk of bias study was excluded | 4 | 707 | | 0.760 (0.529-1.093) | 0% | ns |
| **Outcome 6** | **Stroke** | | | | | | |
| Result 6.1 | Primary analysis (only studies with at least one event were included) | 3 | 728 | | 1.266 (0.305-5.258) | 0% | ns |
| Result 6.2 | Exploratory analysis: All studies that explicitly reported stroke incidence were included (even the ones where event rate was null) | 4 | 870 | | 1.215 (0.319-4.632) | 0% | ns |
| Result 6.3 | Exploratory analysis: Only studies that only included post-ACS patients were included | 2 | 586 | | 0.992 (0.202-4.876) | 0% | ns |
| **Outcome 7** | **CHF** | | | | | | |
| Result 7.1 | Primary analysis | 3 | 301 | | 0.585 (0.077-4.473) | 32% | ns |
| **Outcome 8** | **PCI** | | | | | | |
| Result 8.1 | Primary analysis – only 1 study included; metanalysis not carried out | 1 | | 300 | 0.642 (0.378-1.088) |  | ns |

ns- non statistically significant; s – statistically significant

# Supplemental Material 14 – Metanalyses Results and Forest Plots

**Outcome 1 – All-cause mortality**

**Result 1.1** - All-cause mortality – Primary analysis (only studies with at least one event were included)


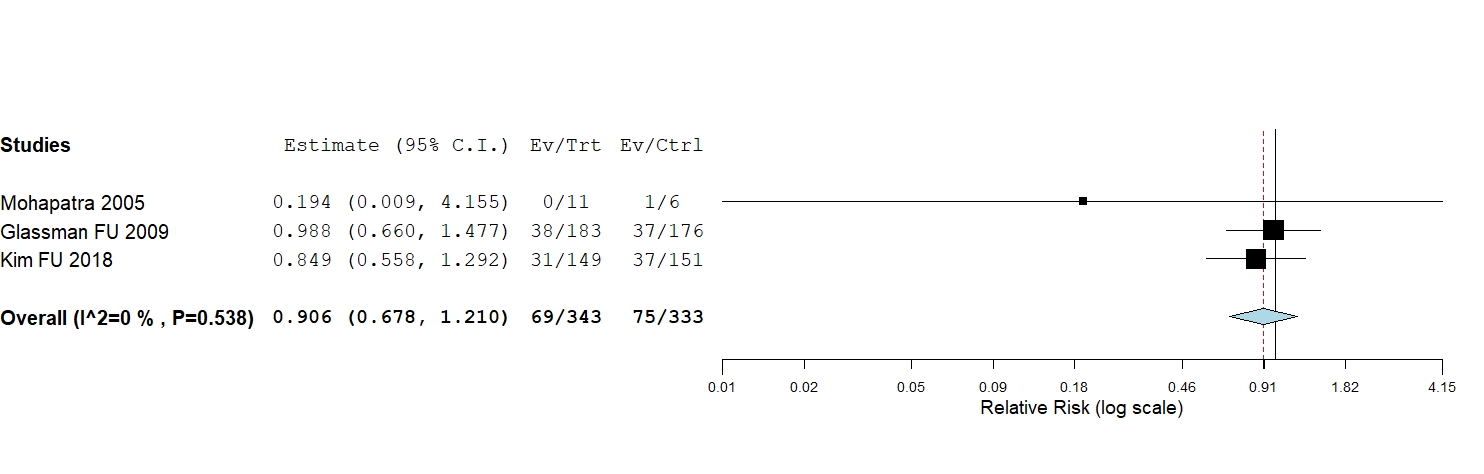


Forest plot for the primary metanalysis of mortality (only studies with at least one event were included): Binary Random-Effects Model; Relative Risk 0.906; 95% CI: 0.678-1.210; p-Value: 0.504. Heterogeneity: tau^2: 0.000; Q(df=2): 1.239; Het p-Value: 0.538; I^2: 0. Study weight: (Mohapatra: 0.893%; Glassman FU: 51.664%; Kim FU: 47.443%)

**Result 1.2** - All-cause mortality – Exploratory analysis: Only studies with at least one event were included; high risk of bias studies were excluded; Same analysis as only FU


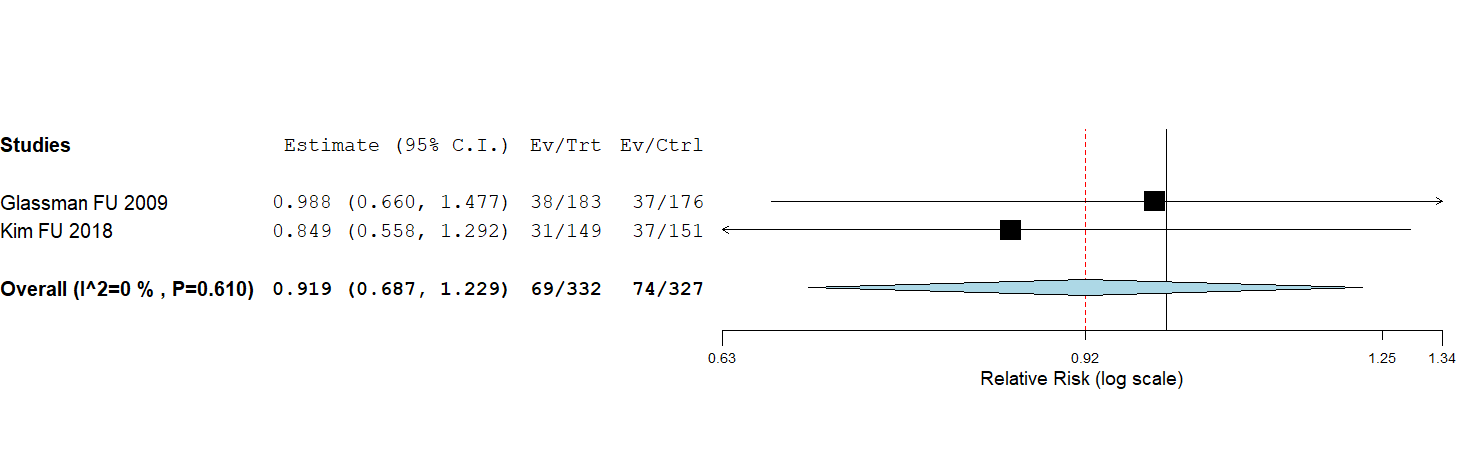


Forest plot for the secondary metanalysis of mortality where only studies with at least one event were included and the high risk of bias study was excluded; Same as the forest plot for the secondary metanalysis of mortality where only FUs were included: Binary Random-Effects Model; Relative Risk 0.919; 95% CI: 0.687-1.229; p-Value: 0.568. Heterogeneity: tau^2: 0.000; Q(df=1): 0.260; Het p-Value: 0.610; I^2: 0. Study weight: (Glassman FU: 52.130%; Kim FU: 47.870%)

**Result 1.3** - All-cause mortality – Exploratory analysis: All studies that explicitly reported mortality were included (even the ones where mortality was null).


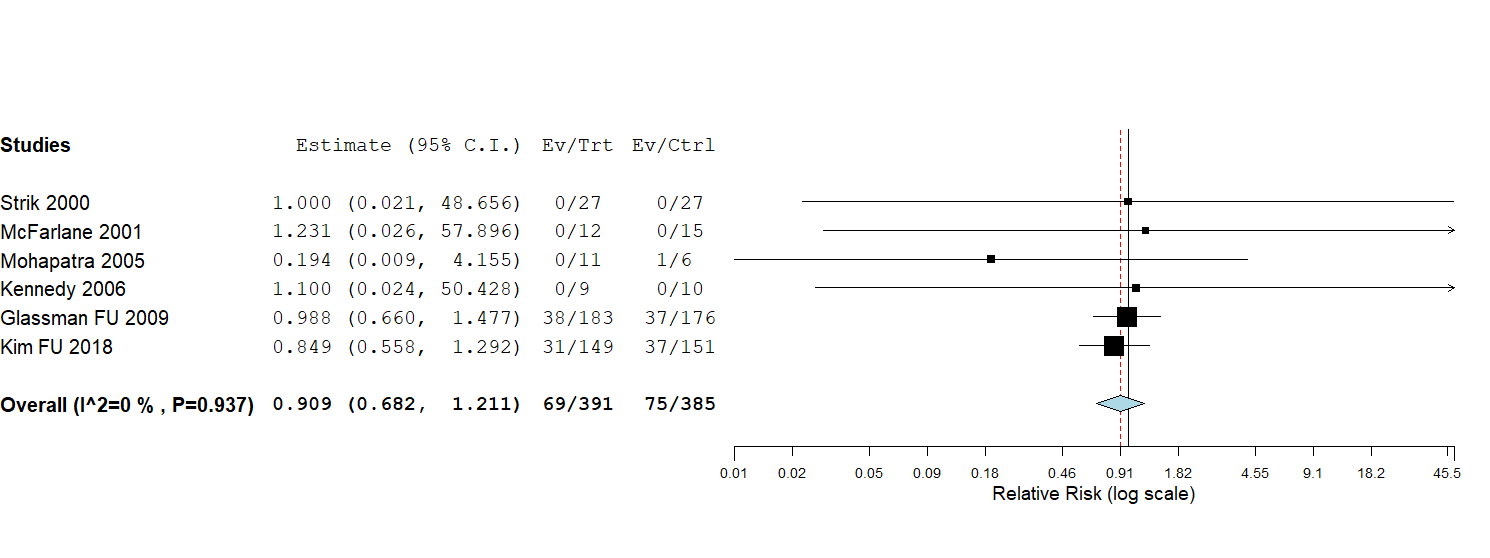


Forest plot for the secondary metanalysis of mortality where all studies that explicitly reported mortality were included (even the ones where mortality was null): Binary Random-Effects Model; Relative Risk 0. 909; 95% CI: 0. 682-1. 211; p-Value: 0.515. Heterogeneity: tau^2: 0.000; Q(df=5): 1.275; Het p-Value: 0.937; I^2: 0. Study weight: (Strik: 0.546%; McFarlane: 0.555%; Mohapatra: 0.878%; Kennedy: 0.563; Glassman FU: 50.804%; Kim FU: 46.653%)

**
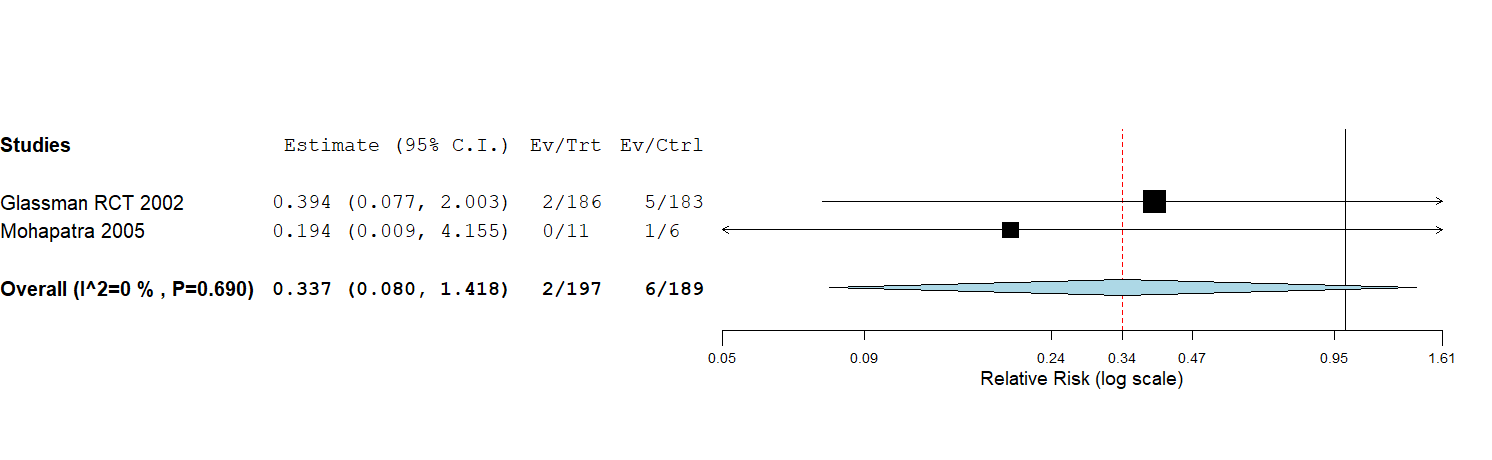
Result 1.4** - All-cause mortality – Exploratory analysis: Only RCTs with at least one event were included.

Forest plot for the secondary metanalysis of mortality where only RCTs with at least one event were included: Binary Random-Effects Model; Relative Risk 0.337; 95% CI: 0. 080-1.418; p-Value: 0.138. Heterogeneity: tau^2: 0.000; Q(df=1): 0.159; Het p-Value: 0.690; I^2: 0. Study weight: (Glassman RCT: 77.980%; Mohapatra: 22.020%)

**Result 1.5** - All-cause mortality – Exploratory analysis: Only RCTs with at least one event were included and the high bias risk studies were excluded

Not carried out because it would only include one study (Glassman RCT; n=369; Ev/Trt: 2/186; Ev/Ctrl: 5/183; RR 0.394, 95% CI: 0.077-2.003).

**
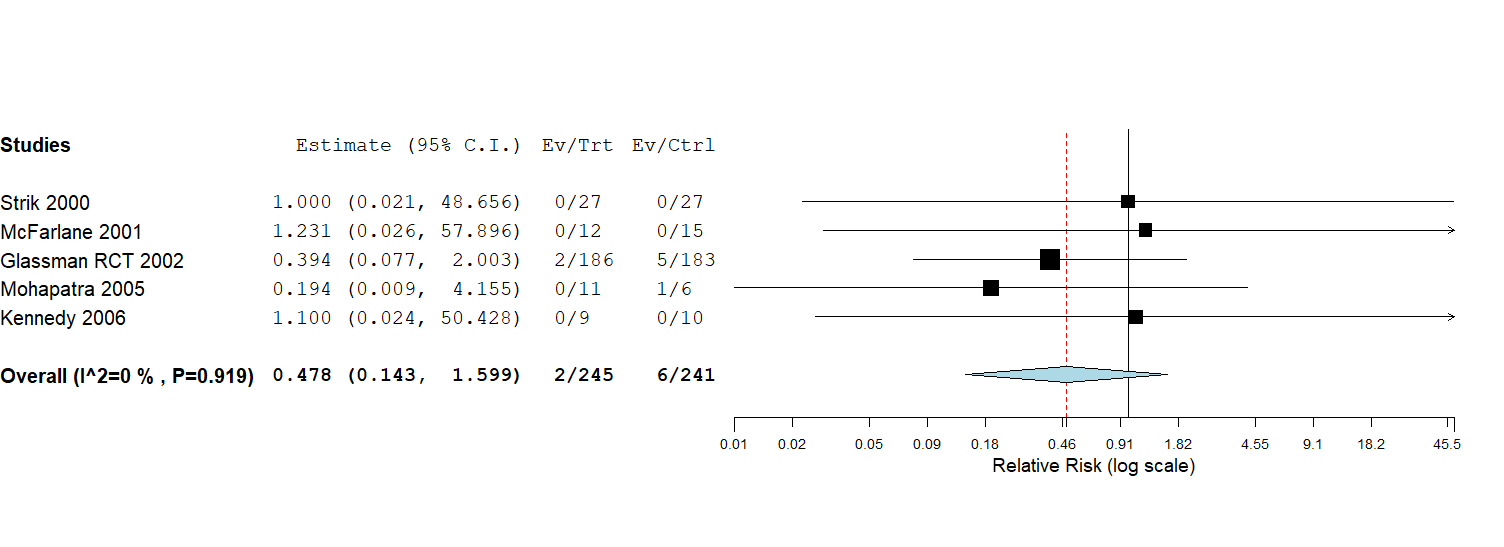
Result 1.6** - All-cause mortality – Exploratory analysis: Only RCTs that explicitly reported mortality were included (even the ones where mortality was null).

Forest plot for the secondary metanalysis of mortality where only RCTs with at least one event were included: Binary Random-Effects Model; Relative Risk 0.478; 95% CI: 0. 143-1.599; p-Value: 0.231. Heterogeneity: tau^2: 0.000; Q(df=4): 0.939; Het p-Value: 0.919; I^2: 0. Study weight: (Strik: 9.653%; McFarlane: 9.823%; Glassman RCT: 55.028%; Mohapatra: 15.539%; Kennedy: 9.956).

**
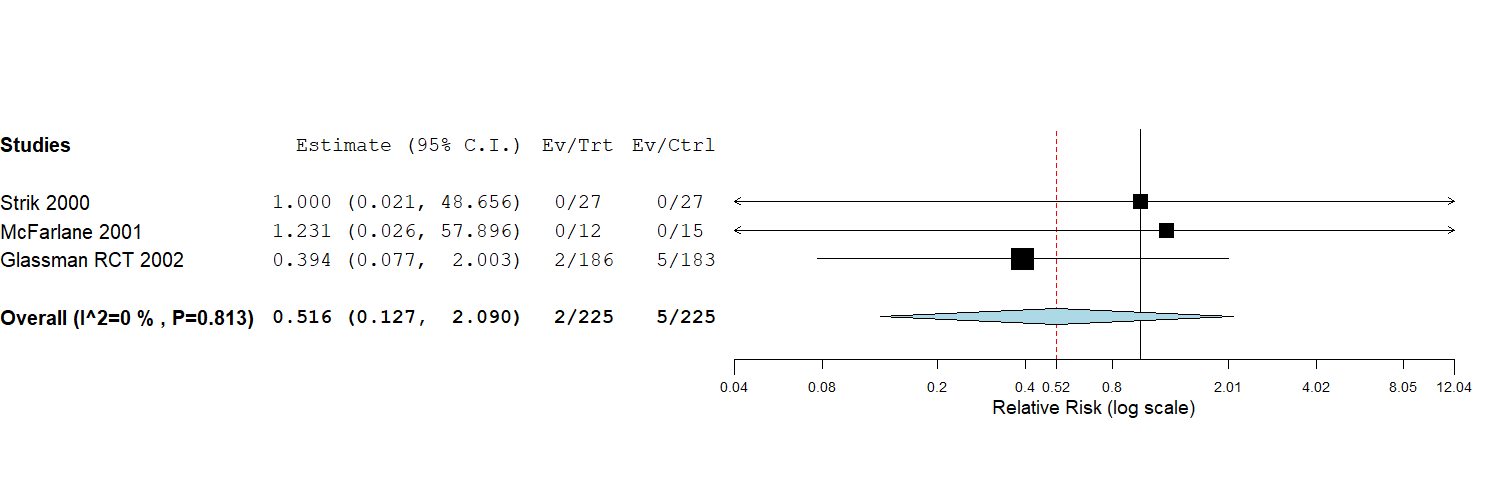
Result 1.7** - All-cause mortality – Exploratory analysis: Only RCTs that explicitly reported mortality were included (even the ones where mortality was null); high bias risk studies were excluded.

Forest plot for the secondary metanalysis of mortality where only RCTs with at least one event were included and the high bias risk studies were excluded: Binary Random-Effects Model; Relative Risk 0.516; 95% CI: 0. 127-2.090; p-Value: 0.354. Heterogeneity: tau^2: 0.000; Q(df=2): 0.414; Het p-Value: 0.813; I^2: 0. Study weight: (Strik: 12.956%; McFarlane: 13.185%; Glassman RCT: 73.859%).

**Outcome 2 –** **Cardiac Mortality**

**Result 2.1** - Cardiac Mortality – Primary analysis

Only one study included (Kim FU; n=300; Ev/Trt: 16/149; Ev/Ctrl: 20/151; RR: 0.811, 95% CI: 0.437-1.503).

Metanalysis not carried out.

**Outcome 3 – Myocardial infarction incidence**

**Result 3.1** - Myocardial infarction incidence – Primary analysis


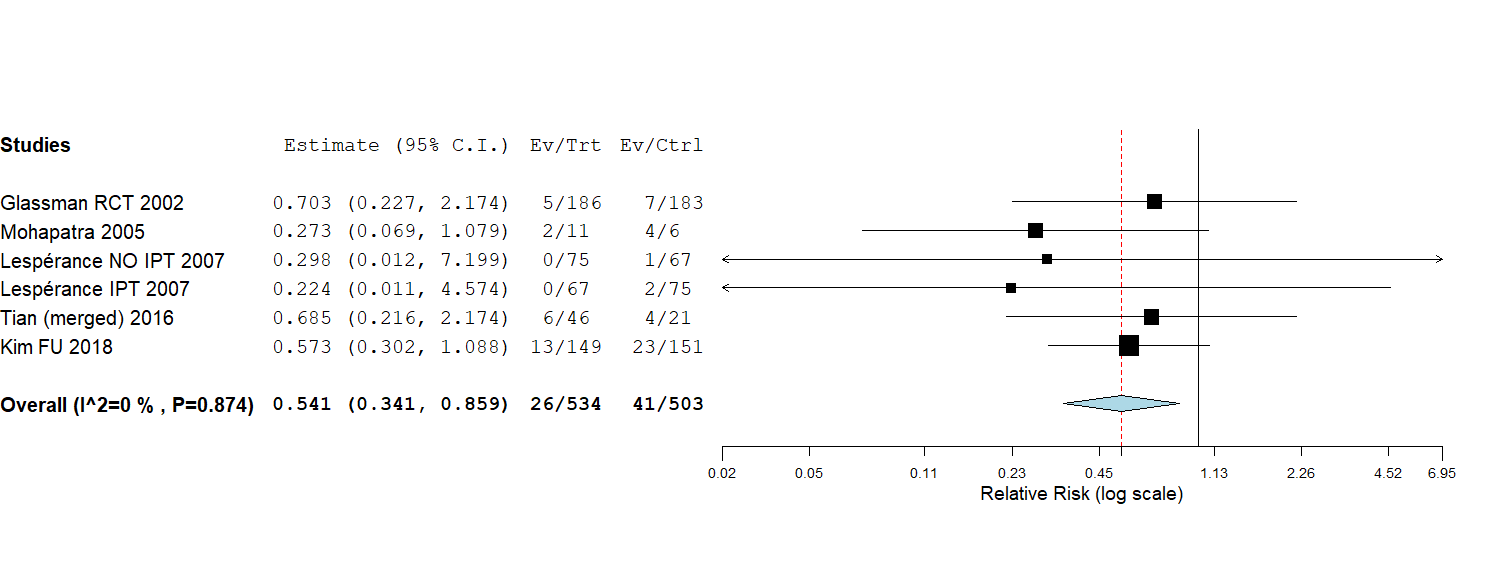


Forest plot for the primary metanalysis of myocardial infarction incidence: Binary Random-Effects Model; Relative Risk 0.541; 95% CI: 0.341-0.859; p-Value: 0.009. Heterogeneity: tau^2: 0.000; Q(df=5): 1.813; Het p-Value: 0.874; I^2: 0.


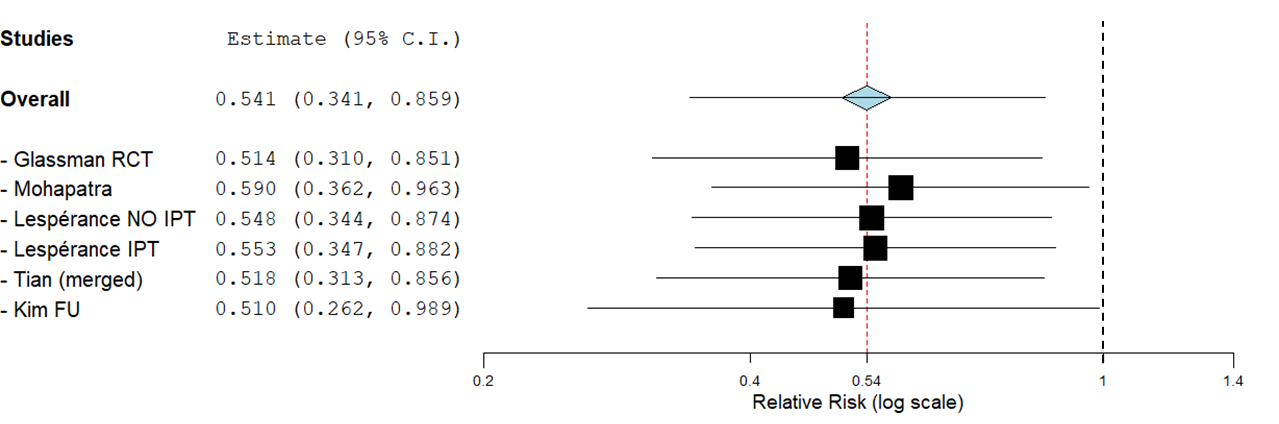
**Result 3.2** - Myocardial infarction incidence – Leave one out (jackknife) analysis of the primary analysis

Forest plot for the leave one out analysis of the primary metanalysis of myocardial infarction incidence (traced black line added manually).

**Result 3.3** - Myocardial infarction incidence – Exploratory analysis: All studies included, except for the high bias risk study


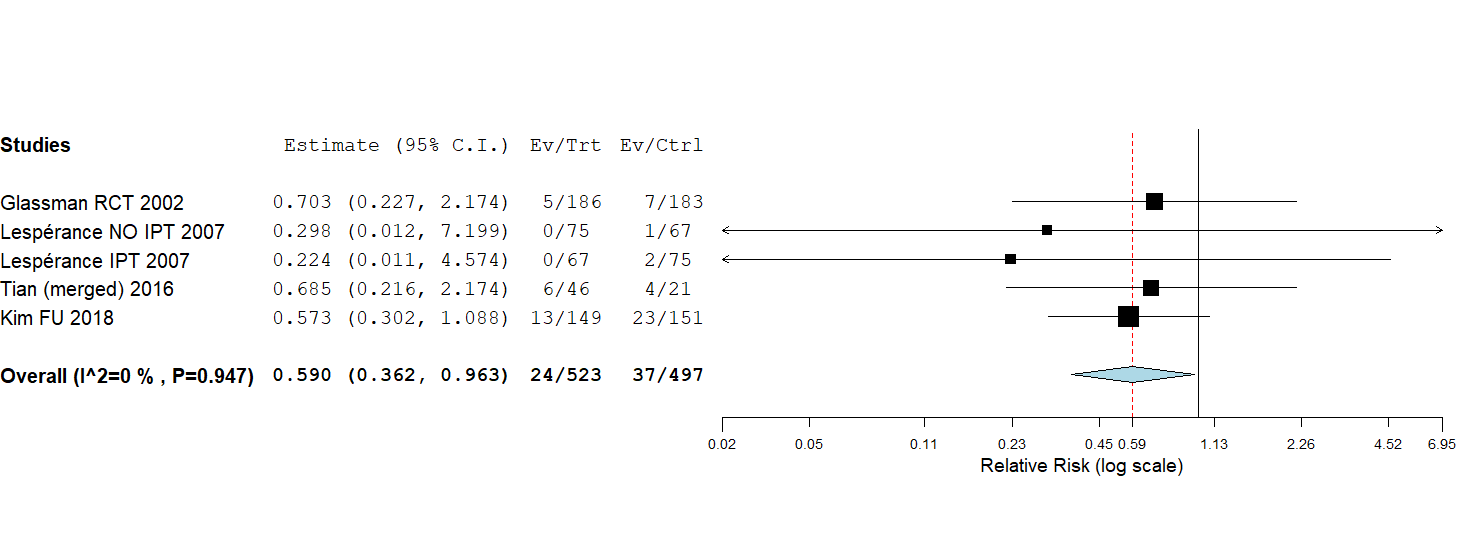


Forest plot for the secondary metanalysis of myocardial infarction incidence where all studies were included, except for the high bias risk study: Binary Random-Effects Model; Relative Risk 0. 590; 95% CI: 0. 362-0. 963; p-Value: 0.035. Heterogeneity: tau^2: 0.000; Q(df=4): 0.738; Het p-Value: 0.947; I^2: 0.

**Result 3.4** - Myocardial infarction incidence – Exploratory analysis: Only studies that only included post-ACS patients were included


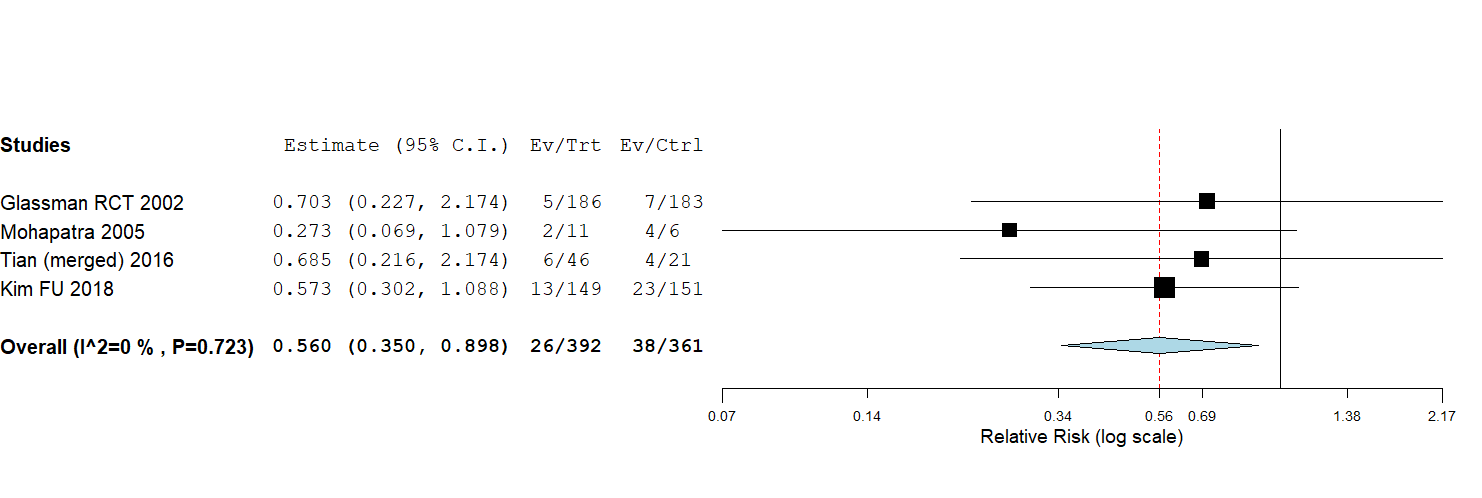


Forest plot for the secondary metanalysis of myocardial infarction incidence where only studies that only included post-ACS patients were included: Binary Random-Effects Model; Relative Risk 0.560; 95% CI: 0. 350-0.898; p-Value: 0.016. Heterogeneity: tau^2: 0.000; Q(df=3): 1.328; Het p-Value: 0.723; I^2: 0. Study weight: (Glassman RCT: 17.448%; Mohapatra: 11.764%; Tian (merged): 16.680%; Kim FU: 54.107%)

**
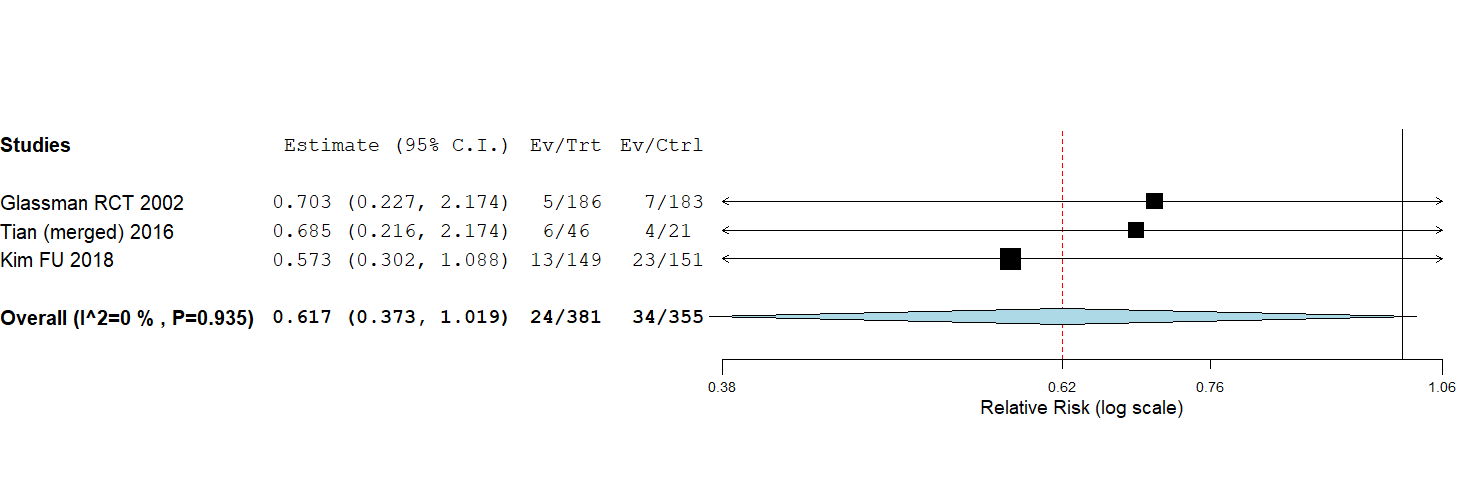
Result 3.5** - Myocardial infarction incidence – Exploratory analysis: Only studies that only included post-ACS patients were included; the high bias risk study was excluded

Forest plot for the secondary metanalysis of myocardial infarction incidence where only studies that only included post-ACS patients were included and the high bias risk study was excluded: Binary Random-Effects Model; Relative Risk 0.617; 95% CI: 0.373-1.019; p-Value: 0.059. Heterogeneity: tau^2: 0.000; Q(df=2): 0.134; Het p-Value: 0.935; I^2: 0. Study weight: (Glassman RCT: 19.775%; Tian (merged): 18.904%; Kim FU: 61.321%)


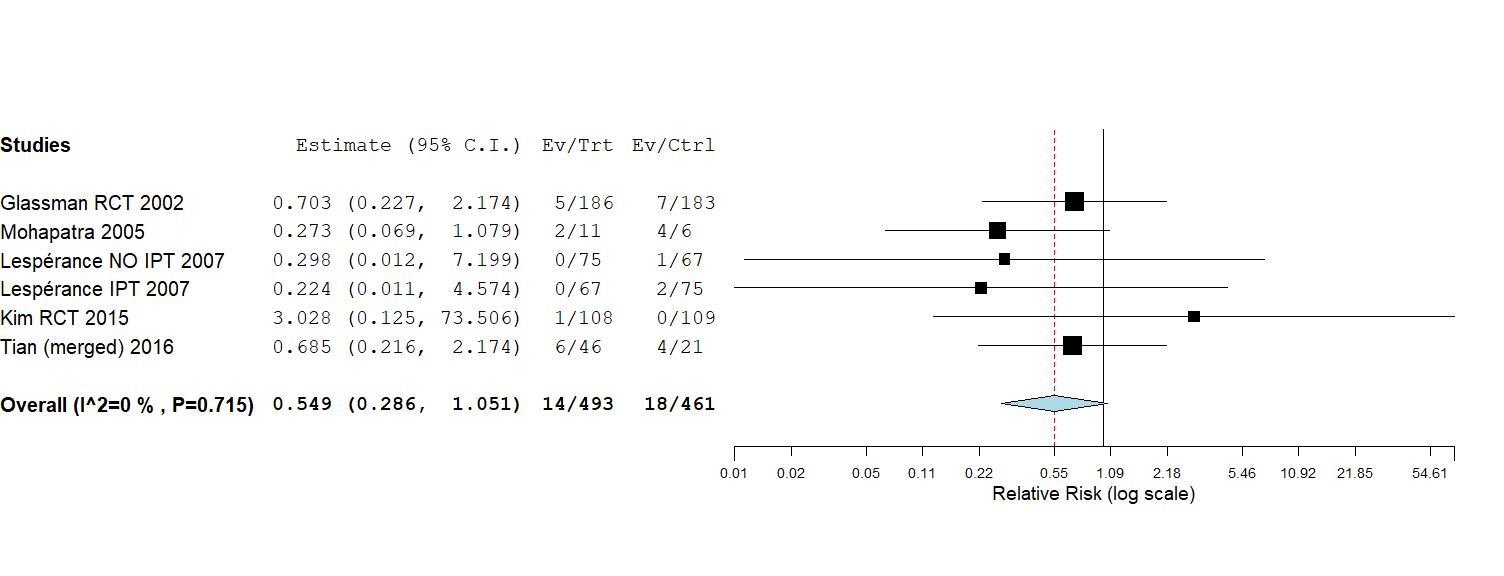
**Result 3.6** - Myocardial infarction incidence – Exploratory analysis: Only RCTs were included

Forest plot for the secondary metanalysis of myocardial infarction incidence where only RCTs were included: Binary Random-Effects Model; Relative Risk 0.549; 95% CI: 0.286-1.051; p-Value: 0.070. Heterogeneity: tau^2: 0.000; Q(df=5): 2.900; Het p-Value: 0.715; I^2: 0.

**Result 3.7** - Myocardial infarction incidence – Exploratory analysis: Only RCTs were included; the high bias risk study was excluded


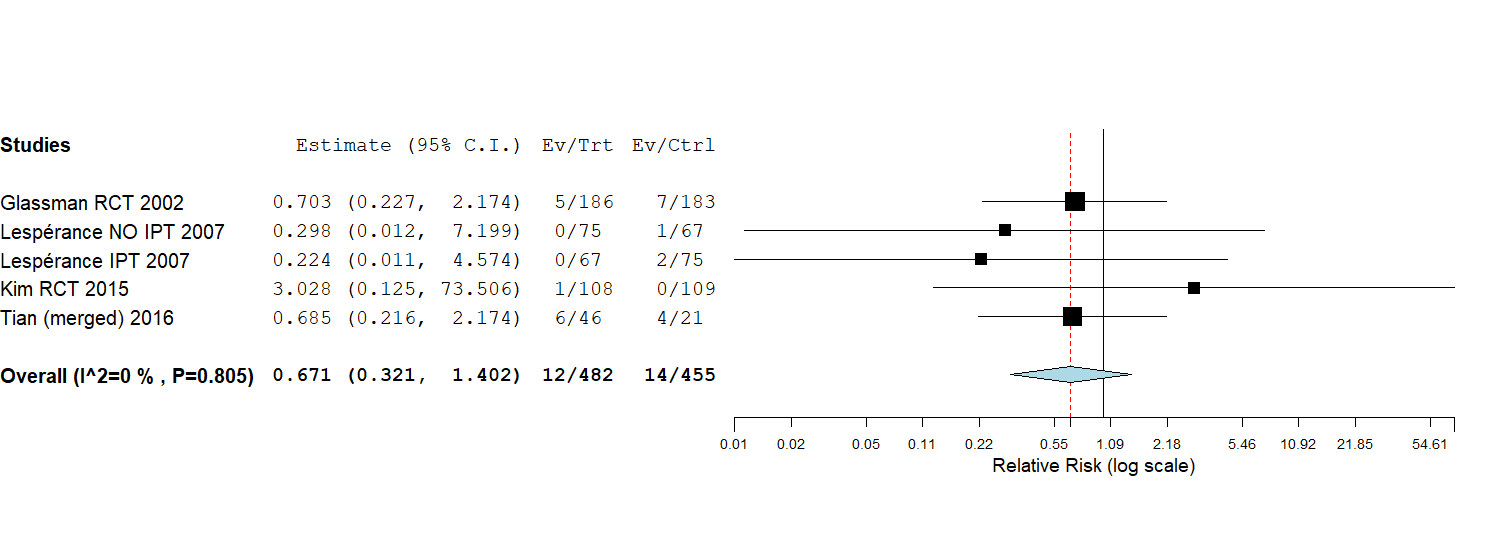


Forest plot for the secondary metanalysis of myocardial infarction incidence where only RCTs were included and the high bias risk study was excluded: Binary Random-Effects Model; Relative Risk 0.671; 95% CI: 0.321-1.402; p-Value: 0.288. Heterogeneity: tau^2: 0.000; Q(df=4): 1.623; Het p-Value: 0.805; I^2: 0.


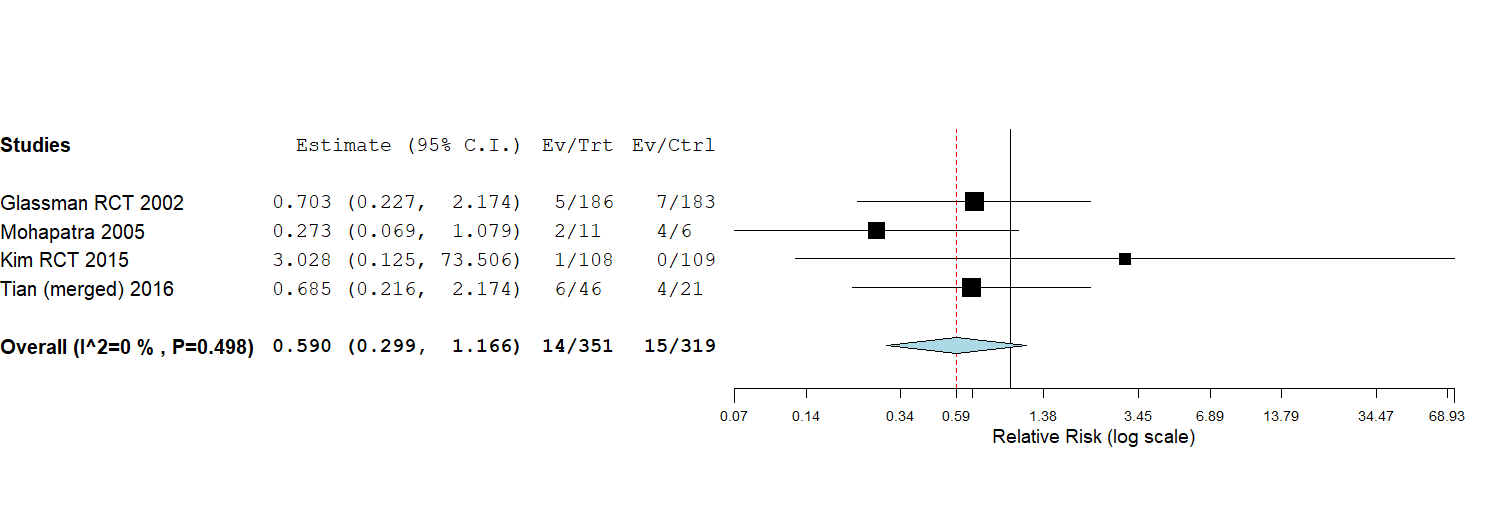
**Result 3.8** - Myocardial infarction incidence – Exploratory analysis: Only RCTs that only included post-ACS patients were included

Forest plot for the secondary metanalysis of myocardial infarction incidence where only RCTs that only included post-ACS patients were included: Binary Random-Effects Model; Relative Risk 0.590; 95% CI: 0.299-1.166; p-Value: 0.129. Heterogeneity: tau^2: 0.000; Q(df=3): 2.375; Het p-Value: 0.498; I^2: 0. Study weight: (Glassman RCT: 36.290%; Mohapatra 24.468%; Kim RCT: 4.549%; Tian (merged): 34.692%)

**Result 3.9** - Myocardial infarction incidence – Exploratory analysis: Only RCTs that only included post-ACS patients were included; the high bias risk study was excluded


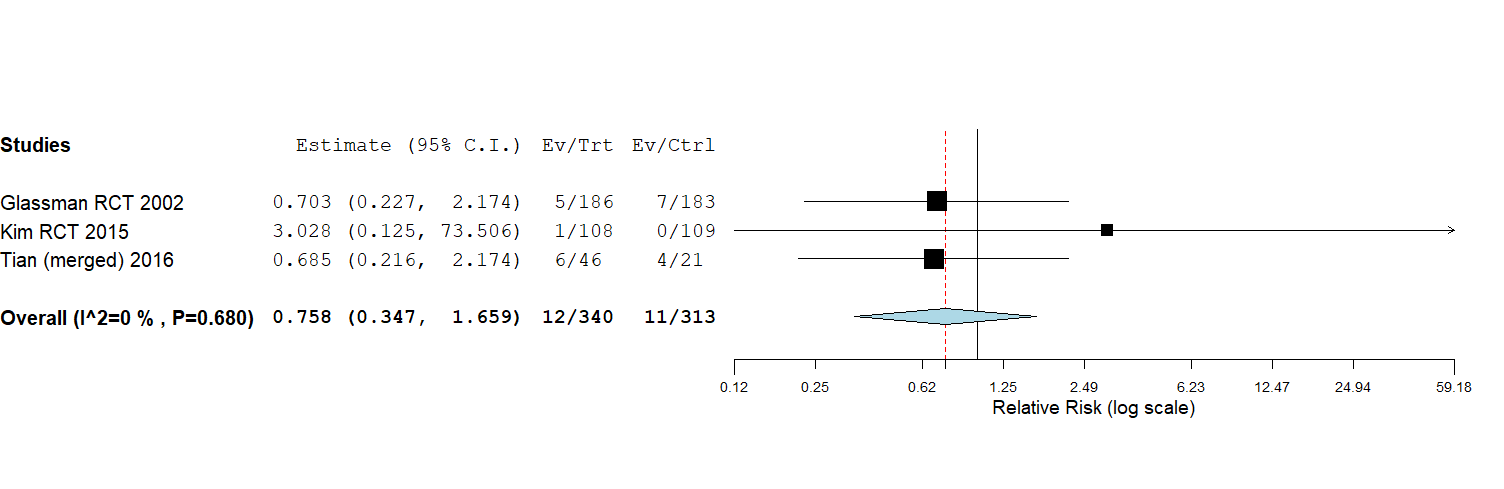


Forest plot for the secondary metanalysis of myocardial infarction incidence where only RCTs that only included post-ACS patients were included and the high bias risk study was excluded: Binary Random-Effects Model; Relative Risk 0.758; 95% CI: 0.347-1.659; p-Value: 0.488. Heterogeneity: tau^2: 0.000; Q(df=2): 0.771; Het p-Value: 0.680; I^2: 0. Study weight: (Glassman RCT: 48.046%; Kim RCT: 6.023%; Tian (merged): 45.931%)

**Result 3.10** - Myocardial infarction incidence – Exploratory analysis: comparison with psychotherapy in both arms excluded


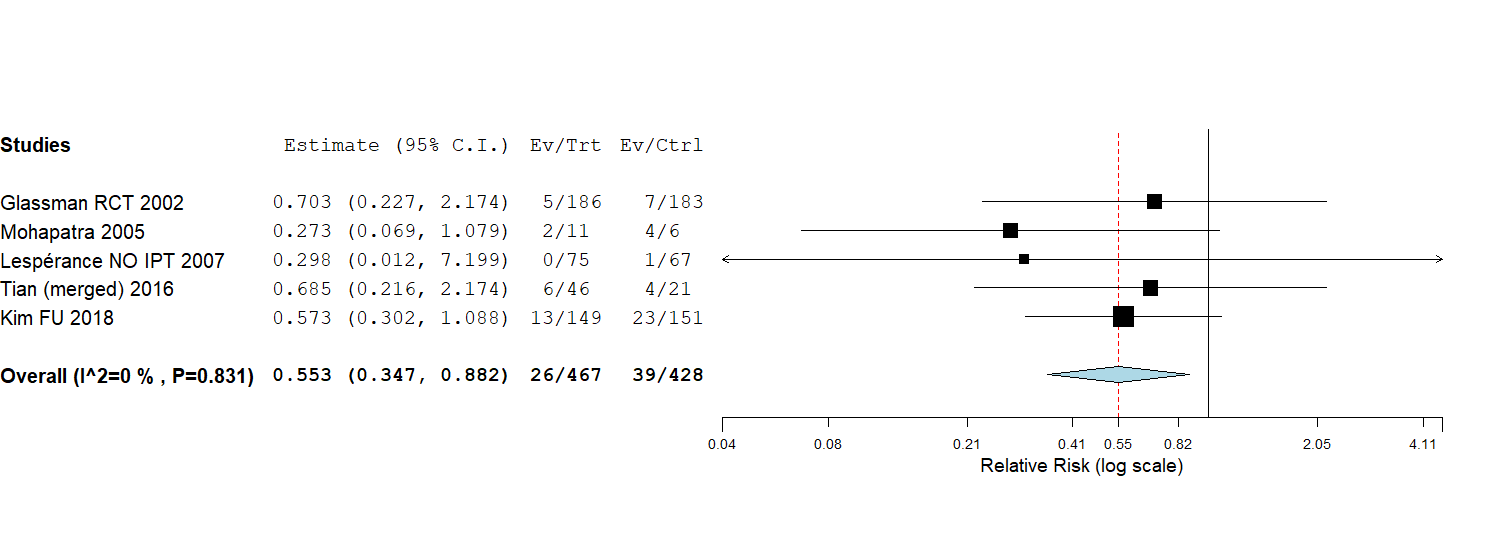


Forest plot for the secondary metanalysis of myocardial infarction incidence where the comparison with psychotherapy in both arms was excluded: Binary Random-Effects Model; Relative Risk 0.553; 95% CI: 0.347-0.882; p-Value: 0.013. Heterogeneity: tau^2: 0.000; Q(df=4): 1.475; Het p-Value: 0.831; I^2: 0.

**Result 3.11** - Myocardial infarction incidence – Exploratory analysis: comparison with psychotherapy in both arms excluded; the high bias risk study was excluded


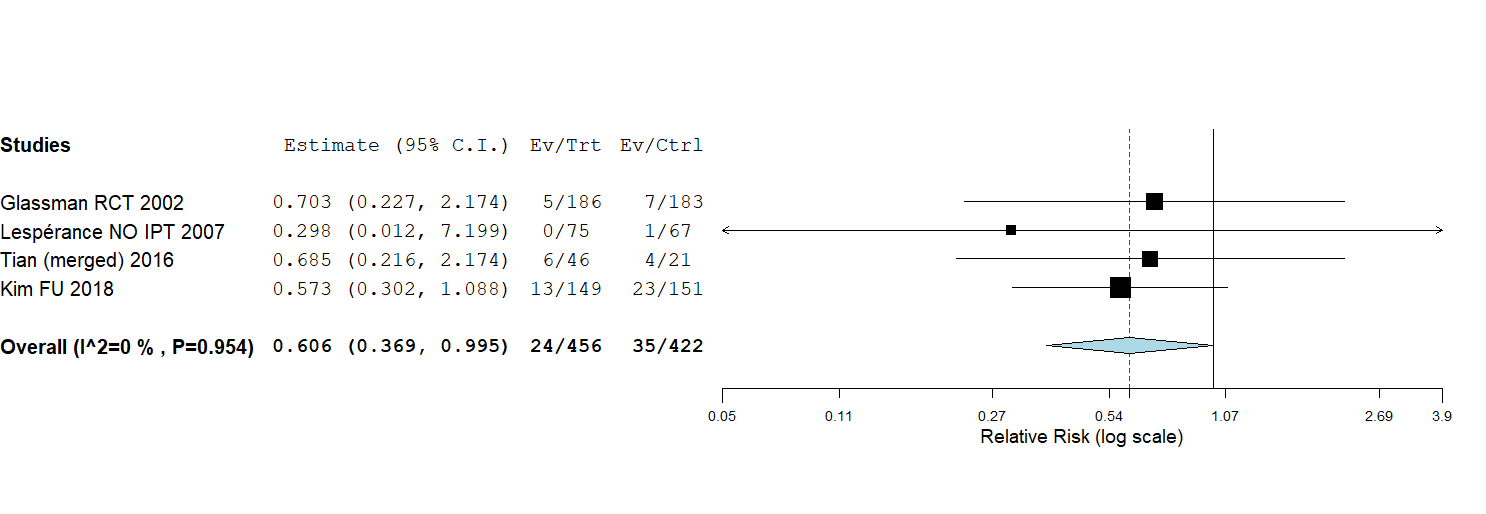


Forest plot for the secondary metanalysis of myocardial infarction incidence where the comparison with psychotherapy in both arms and the high bias risk study were excluded: Binary Random-Effects Model; Relative Risk 0.606; 95% CI: 0.369-0.995; p-Value: 0.048. Heterogeneity: tau^2: 0.000; Q(df=3): 0.329; Het p-Value: 0.954; I^2: 0.

**
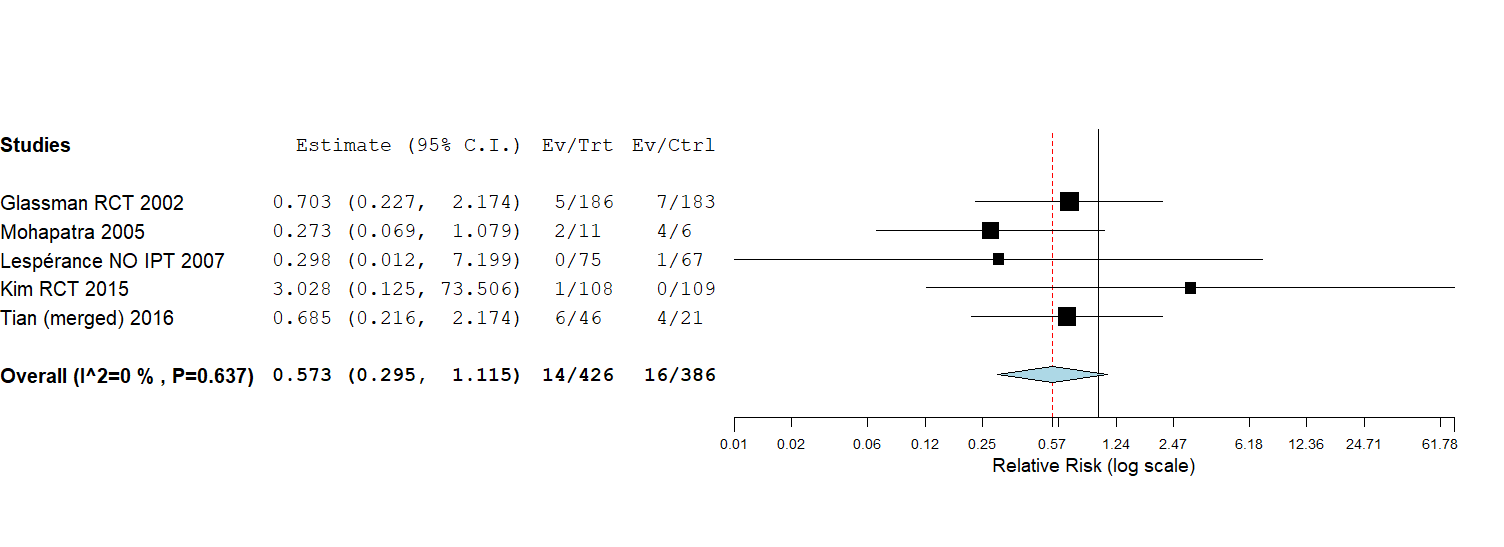
Result 3.12** - Myocardial infarction incidence – Exploratory analysis: Only RCTs; comparison with psychotherapy in both arms excluded

Forest plot for the secondary metanalysis of myocardial infarction incidence where only RCTs were included and the comparison with psychotherapy in both arms was excluded: Binary Random-Effects Model; Relative Risk 0.573; 95% CI: 0.295-1.115; p-Value: 0.101. Heterogeneity: tau^2: 0.000; Q(df=4): 2.544; Het p-Value: 0.637; I^2: 0.


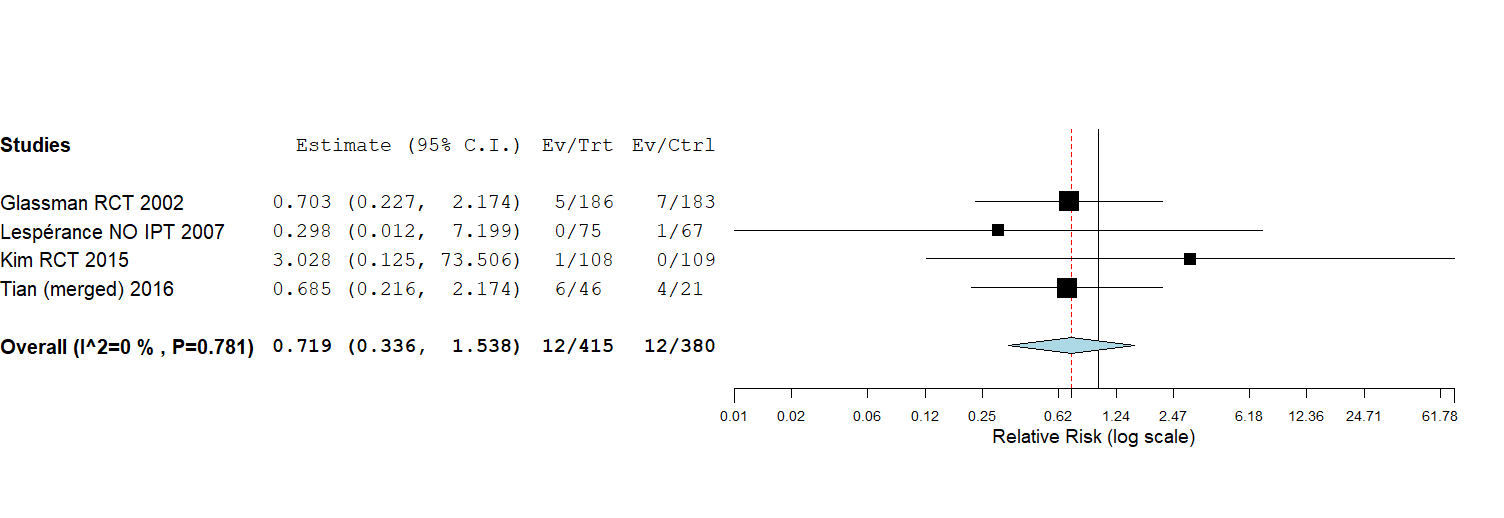
**Result 3.13** - Myocardial infarction incidence – Exploratory analysis: Only RCTs; comparison with psychotherapy in both arms excluded; the high bias risk study was excluded

Forest plot for the secondary metanalysis of myocardial infarction incidence where only RCTs were included and the comparison with psychotherapy in both arms and the high bias risk study were excluded: Binary Random-Effects Model; Relative Risk 0.719; 95% CI: 0.336-1.538; p-Value: 0.395. Heterogeneity: tau^2: 0.000; Q(df=3): 1.082; Het p-Value: 0.781; I^2: 0.

**Outcome 4 – Hospitalizations**

**Result 4.1** - Hospitalizations– Primary analysis


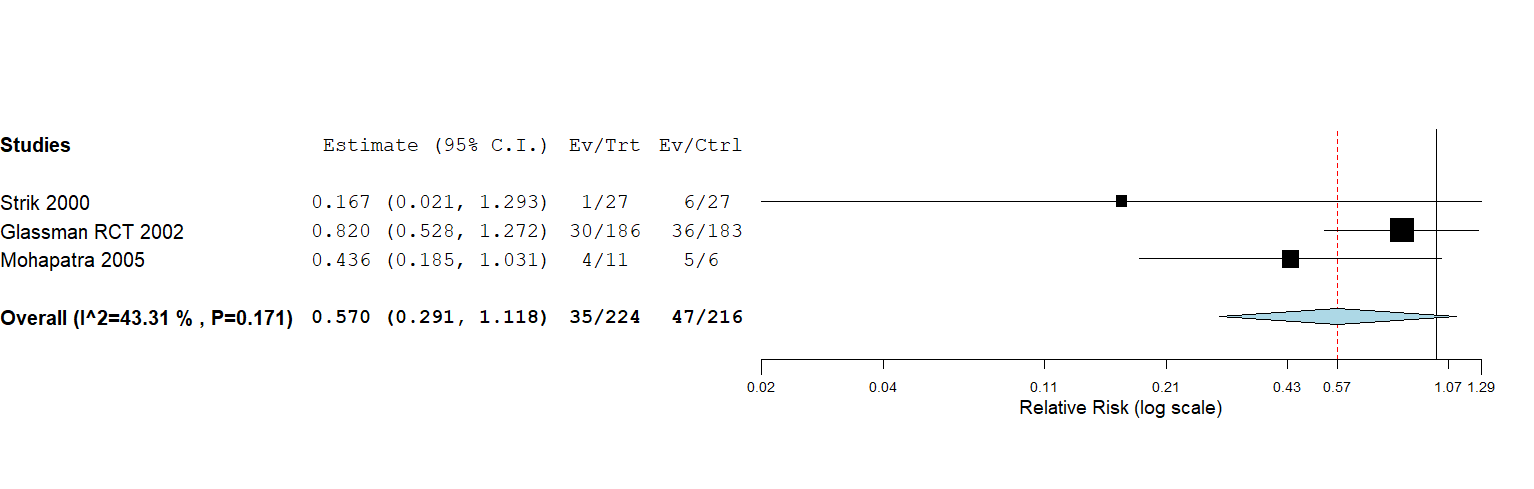


Forest plot for the primary metanalysis of hospitalizations: Binary Random-Effects Model; Relative Risk 0.570; 95% CI: 0.291-1.118; p-Value: 0.102. Heterogeneity: tau^2: 0.157; Q(df=2): 3.528; Het p-Value: 0.171; I^2: 43.308. Study weight: (Strik: 9.436%; Glassman RCT: 56.835%; Mohapatra: 33.729%).

**Result 4.2** - Hospitalizations– Exploratory analysis: All studies included, except for the high bias risk study


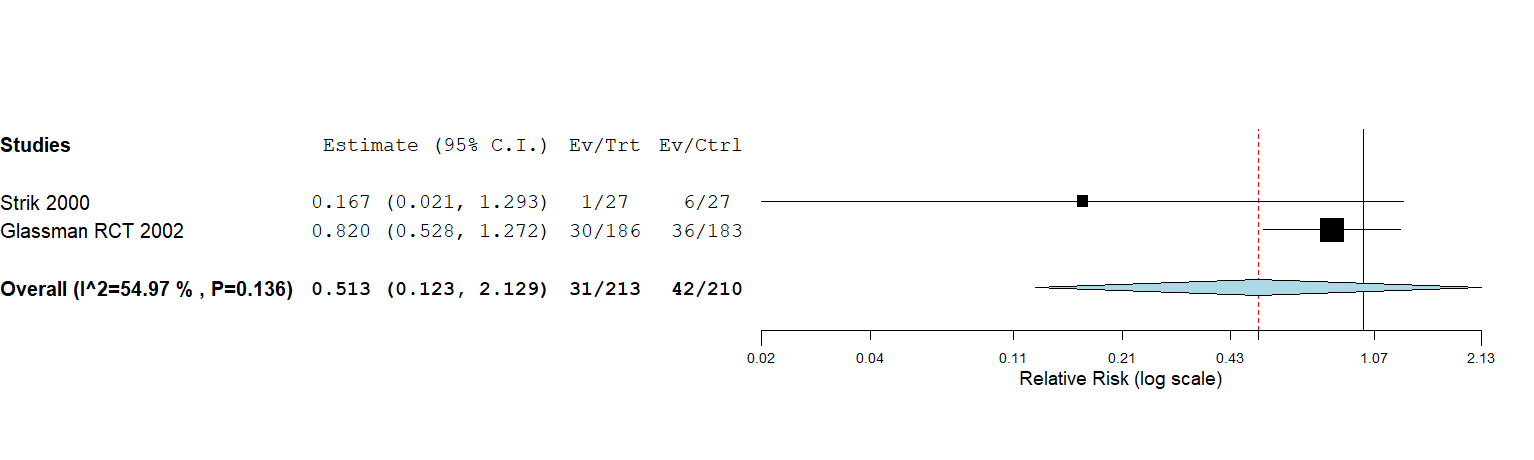


Forest plot for the secondary metanalysis of hospitalization incidence where all studies were included, except for the high bias risk study: Binary Random-Effects Model; Relative Risk 0.513; 95% CI: 0.123-2.129; p-Value: 0.358. Heterogeneity: tau^2: 0.698; Q(df=1): 2.221; Het p-Value: 0.136; I^2: 54.974. Study weight: (Strik: 29.467%; Glassman RCT: 70.533%).

**Outcome 5 – Angina**

**Result 5.1** - Angina– Primary analysis


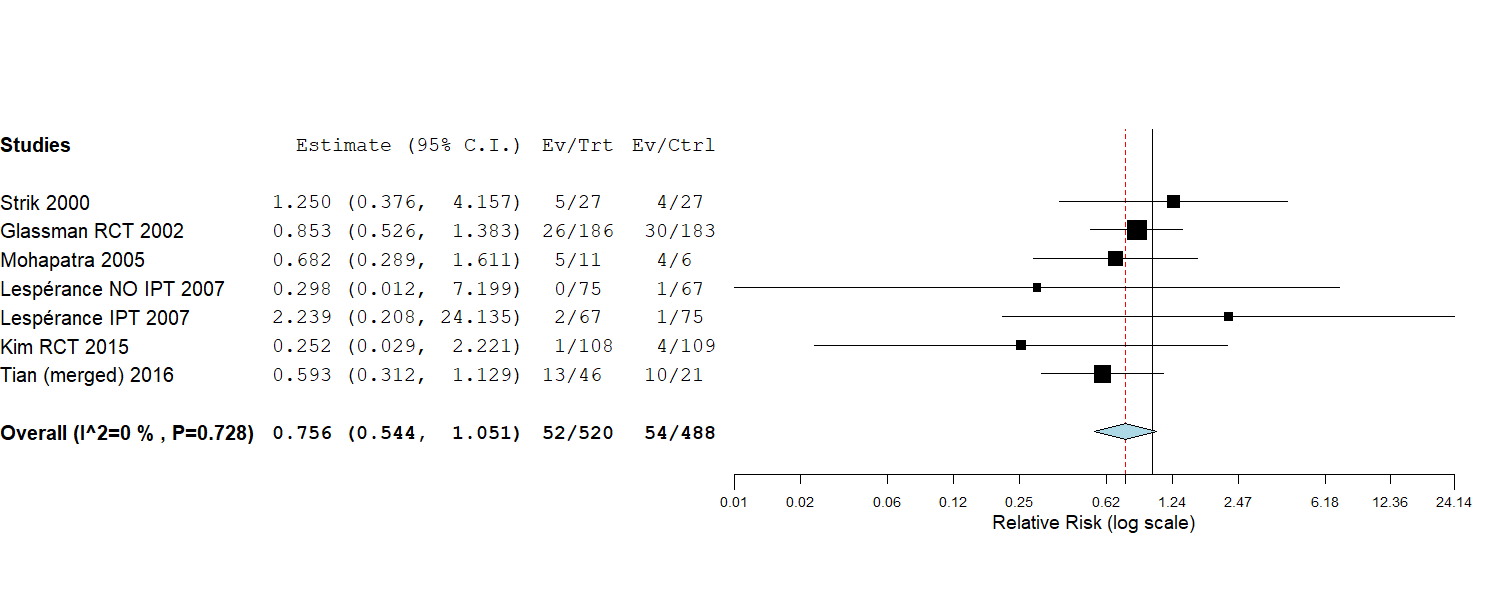


Forest plot for the primary metanalysis of angina prevalence: Binary Random-Effects Model; Relative Risk 0.756; 95% CI: 0.544-1.051; p-Value: 0.097. Heterogeneity: tau^2: 0.000; Q(df=6): 3.617; Het p-Value: 0.728; I^2: 0.

**Result 5.2** - Angina– Exploratory analysis: All studies included, except for the high bias risk study


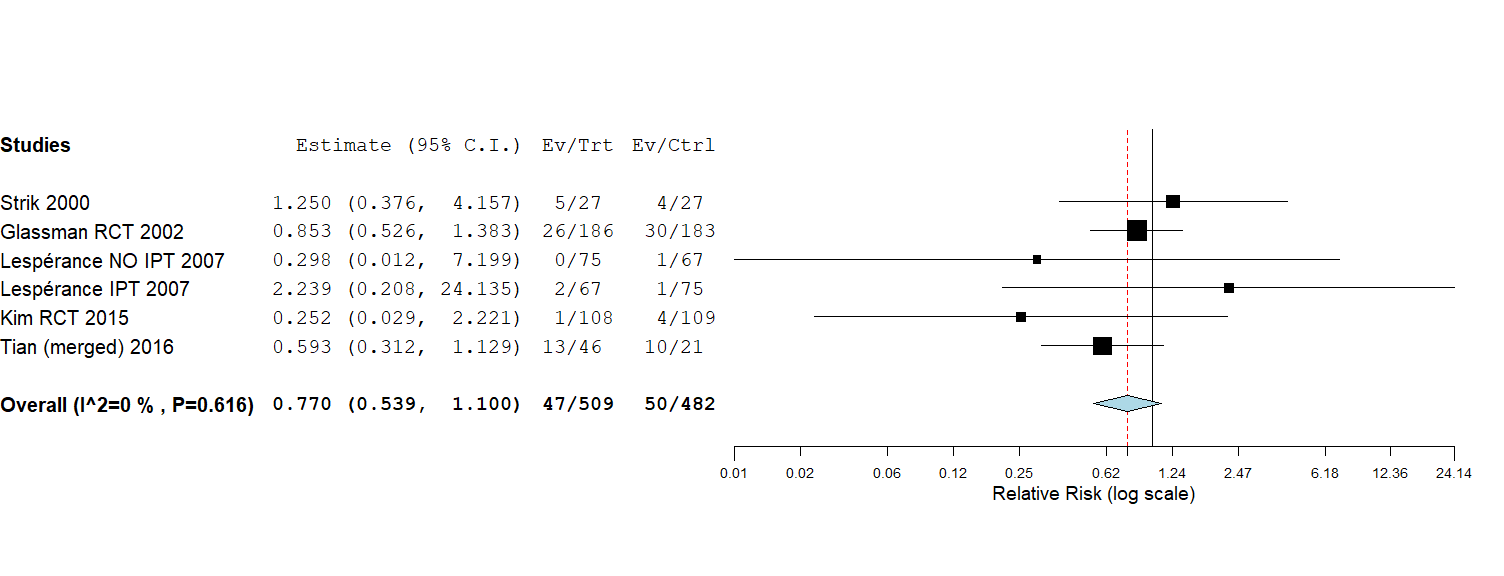


Forest plot for the secondary metanalysis of angina prevalence where all studies were included, except for the high bias risk study: Binary Random-Effects Model; Relative Risk 0.770; 95% CI: 0.539-1.100; p-Value: 0.151. Heterogeneity: tau^2: 0.000; Q(df=5): 3.551; Het p-Value: 0.616; I^2: 0.


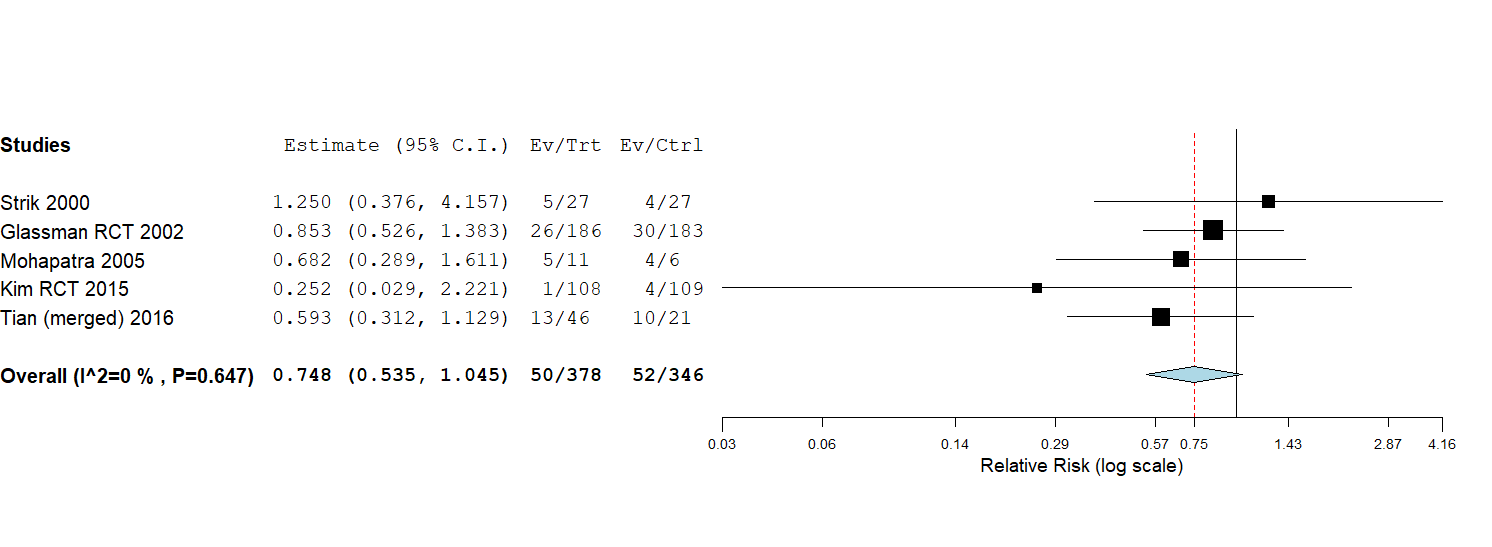
**Result 5.3** - Angina– Exploratory analysis: Only studies that only included post-ACS patients were included

Forest plot for the secondary metanalysis of angina prevalence where only studies that only included post-ACS patients were included: Binary Random-Effects Model; Relative Risk 0.748; 95% CI: 0.535-1.045; p-Value: 0.089. Heterogeneity: tau^2: 0.000; Q(df=4): 2.484; Het p-Value: 0.647; I^2: 0. Study weight: (Strik: 7.739%; Glassman RCT: 47.731%; Mohapatra: 15.120%; Kim RCT: 2.362%; Tian (merged): 27.048%).

**Result 5.4** - Angina– Exploratory analysis: Only studies that only included post-ACS patients were included; the high risk of bias study was excluded


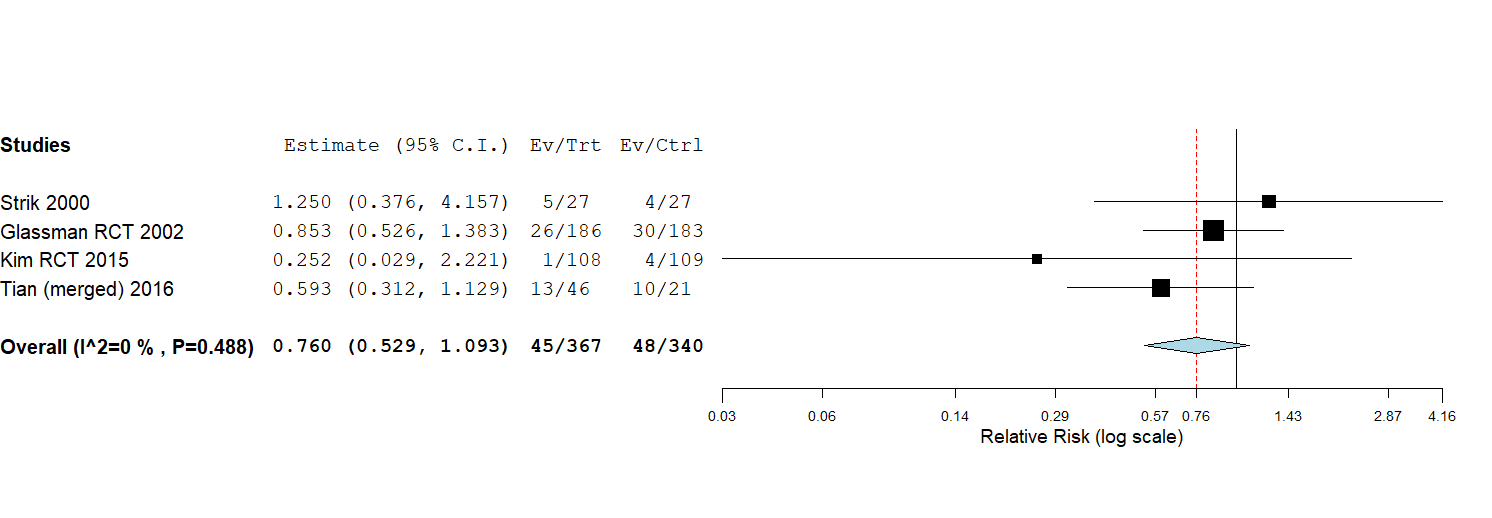


Forest plot for the secondary metanalysis of angina prevalence where only studies that only included post-ACS patients were included and the high bias risk study was excluded: Binary Random-Effects Model; Relative Risk 0.760; 95% CI: 0.529-1.093; p-Value: 0.139. Heterogeneity: tau^2: 0.000; Q(df=3): 2.432; Het p-Value: 0.488; I^2: 0. Study weight: (Strik: 9.1189%; Glassman RCT: 56.233%; Kim RCT: 2.783%; Tian (merged): 31.866%).

**Outcome 6 – Stroke**


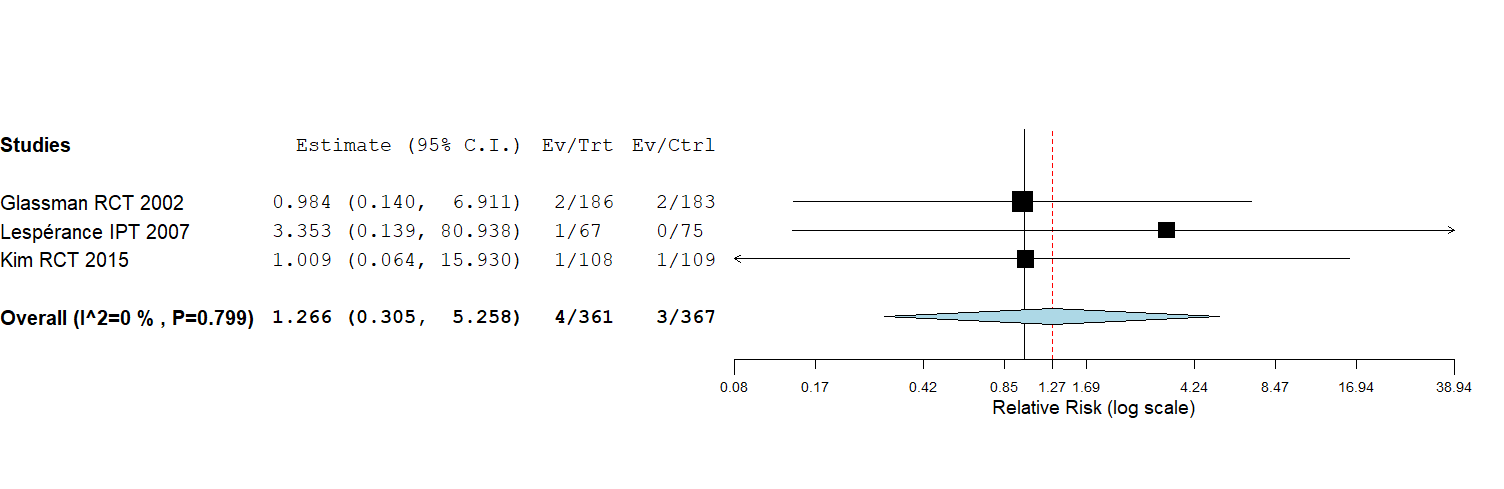
**Result 6.1** - Stroke– Primary analysis (only studies with at least one event were included)

Forest plot for the primary metanalysis of stroke incidence: Binary Random-Effects Model; Relative Risk 1.266; 95% CI: 0.305-5.258; p-Value: 0.746. Heterogeneity: tau^2: 0.000; Q(df=2): 0.450; Het p-Value: 0.799; I^2: 0.

**Result 6.2** – Stroke – Exploratory analysis: All studies that explicitly reported stroke incidence were included (even the ones where event rate was null).


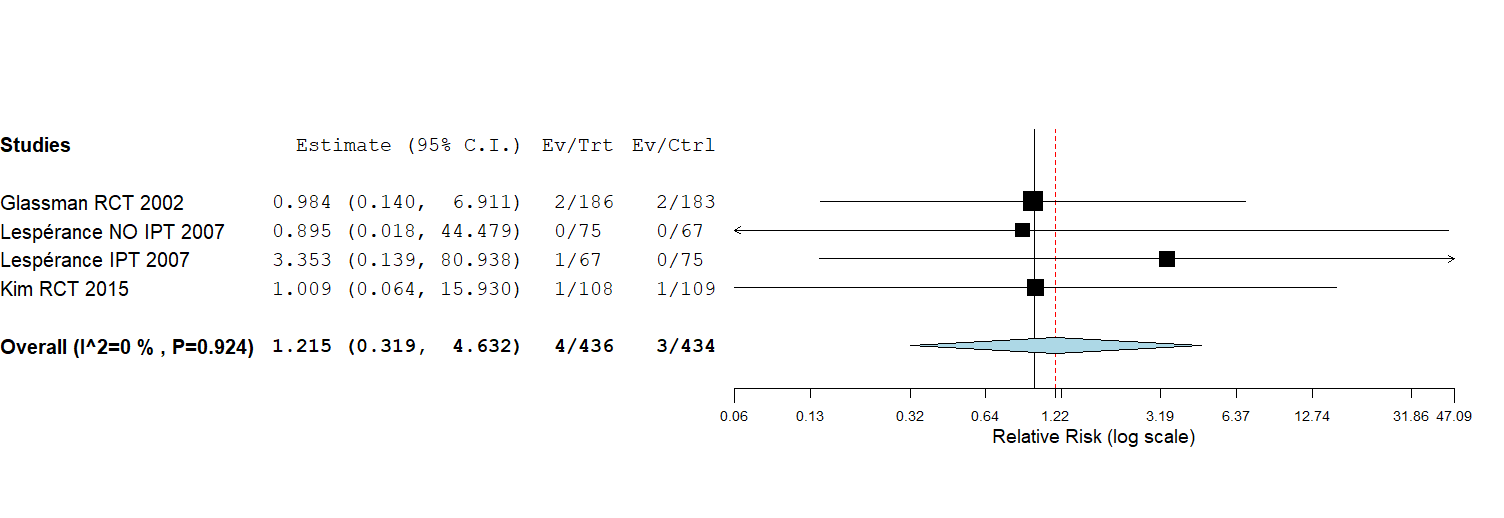


Forest plot for the secondary metanalysis of stroke incidence where all studies that explicitly reported stroke incidence were included (even the ones where event rate was null): Binary Random-Effects Model; Relative Risk 1.215; 95% CI: 0.319-4.632; p-Value: 0.775. Heterogeneity: tau^2: 0.000; Q(df=3): 0.476; Het p-Value: 0.924; I^2: 0.

**Result 6.3** – Stroke – Exploratory analysis: Only studies that only included post-ACS patients were included.


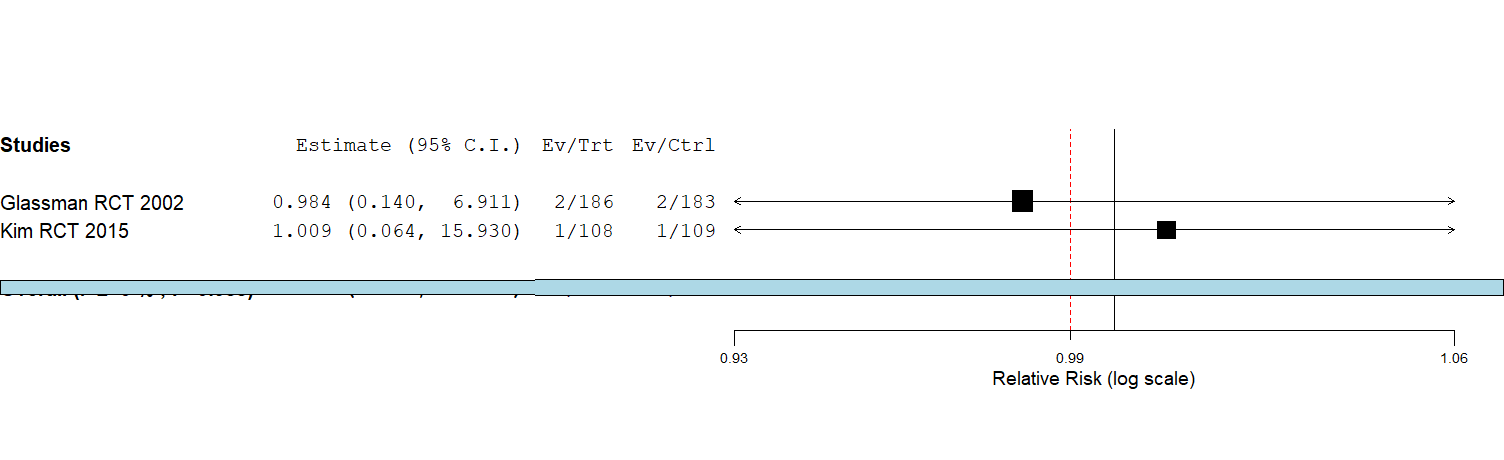


Forest plot for the secondary metanalysis of stroke incidence where all studies that explicitly reported stroke incidence were included (even the ones where event rate was null): Binary Random-Effects Model; Relative Risk 0.992; 95% CI: 0.202-4.876; p-Value: 0.992. Heterogeneity: tau^2: 0.000; Q(df=1): 0.000; Het p-Value: 0.988; I^2: 0. Study weight: (Glassman RCT: 66.703%; Kim RCT: 33.297%)

**Outcome 7 – CHF**

**Result 7.1** – CHF – Primary analysis


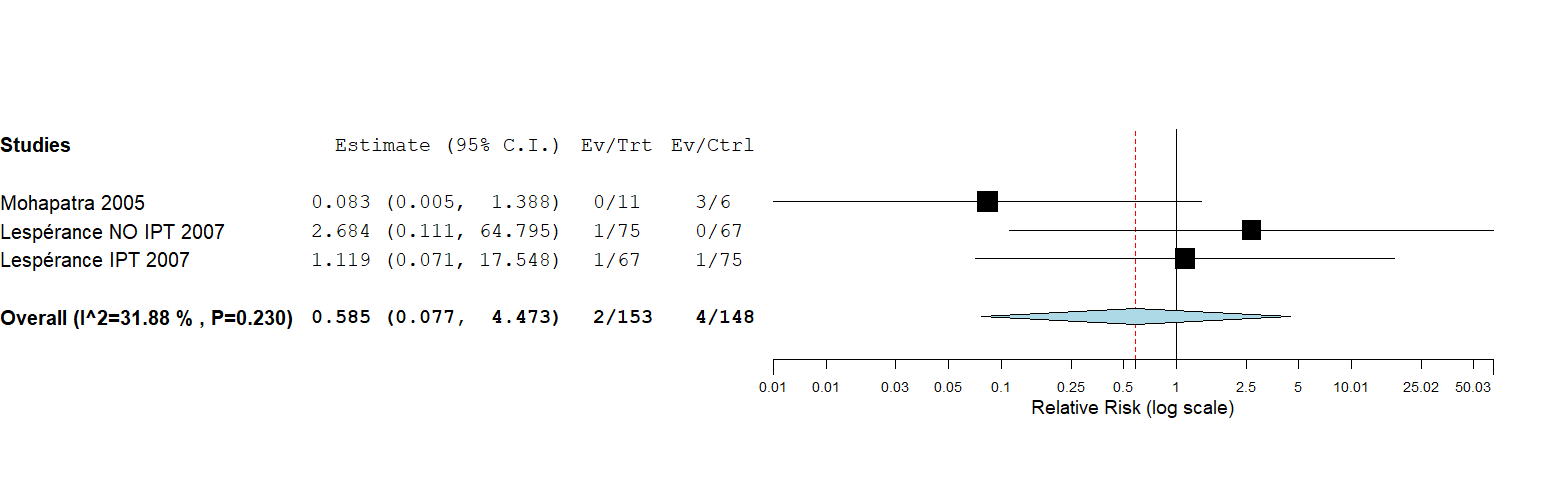


Forest plot for the primary metanalysis of CHF: Binary Random-Effects Model; Relative Risk 0.585; 95% CI: 0.077-4.473; p-Value: 0.606. Heterogeneity: tau^2: 1.031; Q(df=2): 2.936; Het p-Value: 0.230; I^2: 31.88.

**Outcome 8 – PCI**

**Result 8.1** – PCI – Primary analysis

Only one study included (Kim FU; n=300; Ev/Trt: 19/149; Ev/Ctrl: 30/151; RR: 0.642, 95% CI: 0.378-1.088).

Metanalysis not carried out.

# Supplemental Material 15 – GRADE Evidence Profile

| **Certainty assessment** | | | | | | | **№ of patients** | | **Effect** | | **Certainty** | **Importance** |
| --- | --- | --- | --- | --- | --- | --- | --- | --- | --- | --- | --- | --- |
| **№ of studies** | **Study design** | **Risk of bias** | **Inconsistency** | **Indirectness** | **Imprecision** | **Other considerations** | **SSRIs** | **No SSRIs** | **Relative (95% CI)** | **Absolute (95% CI)** |  |  |
| All-cause mortality (follow-up: range 6 months to 8.1 years; assessed with: RR) | | | | | | | | | | | | |
| 3 | RCT+FU | serious | not serious | not serious | serious | none | 69/343 (20.1%) | 75/333 (22.5%) | RR 0.906 (0.678 to 1.210) | 21 fewer per 1 000 (from 73 fewer to 47 more) | ⨁⨁◯◯ LOW | CRITICAL |
| Cardiovascular Mortality (follow-up: median 8.1 years; assessed with: RR) | | | | | | | | | | | | |
| 1 | FU | serious | not serious | not serious | serious | none | 16/149 (10.7%) | 20/151 (13.2%) | RR 0.811 (0.437 to 1.503) | 25 fewer per 1 000 (from 75 fewer to 67 more) | ⨁⨁◯◯ LOW | CRITICAL |
| Myocardial Infarction (follow-up: range 8 weeks to 8.1 years; assessed with: RR) | | | | | | | | | | | | |
| 6 | RCT+FU | serious | not serious | not serious | serious | none | 26/534 (4.9%) | 41/503 (8.2%) | RR 0.541 (0.341 to 0.859) | 37 fewer per 1 000 (from 54 fewer to 11 fewer) | ⨁⨁◯◯ LOW | CRITICAL |
| Angina (follow-up: range 8 weeks to 6 months; assessed with: RR) | | | | | | | | | | | | |
| 7 | RCT | serious | not serious | serious | serious | none | 52/520 (10.0%) | 54/488 (11.1%) | RR 0.756 (0.544 to 1.051) | 27 fewer per 1 000 (from 50 fewer to 6 more) | ⨁◯◯◯ VERY LOW | IMPORTANT |
| Hospitalizations (follow-up: mean 6 months; assessed with: RR) | | | | | | | | | | | | |
| 3 | RCT | serious | not serious | not serious | serious | none | 35/224 (15.6%) | 47/216 (21.8%) | RR 0.570 (0.291 to 1.118) | 94 fewer per 1 000 (from 154 fewer to 26 more) | ⨁⨁◯◯ LOW | IMPORTANT |
| Stroke (follow-up: range 12 weeks to 24 weeks; assessed with: RR) | | | | | | | | | | | | |
| 3 | RCT | serious | not serious | not serious | serious | none | 4/361 (1.1%) | 3/367 (0.8%) | RR 1.266 (0.305 to 5.258) | 2 more per 1 000 (from 6 fewer to 35 more) | ⨁⨁◯◯ LOW | CRITICAL |
| Congestive Heart Failure (follow-up: range 12 weeks to 6 months; assessed with: RR) | | | | | | | | | | | | |
| 3 | RCT | serious | not serious | not serious | serious | none | 2/153 (1.3%) | 4/148 (2.7%) | RR 0.585 (0.077 to 4.473) | 11 fewer per 1 000 (from 25 fewer to 94 more) | ⨁⨁◯◯ LOW | IMPORTANT |
| Percutaneous Coronary Intervention (follow-up: median 8.1 years; assessed with: RR) | | | | | | | | | | | | |
| 1 | FU | serious | not serious | not serious | serious | none | 19/149 (12.8%) | 30/151 (19.9%) | RR 0.642 (0.378 to 1.088) | 71 fewer per 1 000 (from 124 fewer to 17 more) | ⨁⨁◯◯ LOW | IMPORTANT |
|  | | | | | | | | | | | | |
| Relevant commentaries: | | | | | | | | | | | | |
| Risk of bias: | Most studies included were of uncertain risk of bias (some concerns in the RoB2 terminology). | | | | | | | | | | | |
| Inconsistency: | All but the metanalyses for hospitalizations and CHF had I^2^ of 0%. All primary metanalyses had an I^2^ below the pre-established cut-off value of 50%. There was no substantial heterogenicity nor non-overlapping CIs of the different studies included for each outcome. | | | | | | | | | | | |
| Indirectness: | The differences in population characteristics were considered not substantial enough to downgrade the certainty of the evidence. The differences between interventions were not considered to be substantial enough to downgrade the certainty of the evidence (as we assumed a class effect of SSRIs). The definition of the outcomes and the method used to assess the occurrence of events was not considered to be substantial enough to downgrade the certainty of the evidence (except for the outcome of angina); no surrogate outcomes nor indirect comparisons were used. | | | | | | | | | | | |
| Imprecision: | MI - Downgraded for optimal information size criterion is being met; All other outcomes – Downgraded for optimal information size criterion is being met and 95% CI overlapping no effect. | | | | | | | | | | | |
| Other: | No large magnitude effects (Grade definition: RR* >2 or <0.5) were found; No dose-response gradient was assessed. We cannot guaranty that small RCTs were not carried out but not published, however, our search was comprehensive (and conservative in the exclusion in the title and abstract phase) and even returned an unpublished study. As a result, publication bias risk was not considered high enough to warrant downgrading the quality of the evidence, although no funnel plot was created and assessed (due to the low number of studies included for each outcome). | | | | | | | | | | | |

# Supplemental Material 16 – GRADE Summary of Findings

| **Outcomes**  assessed with RR | **№ of participants  (studies)** | **Anticipated absolute effects* (95% CI)** | | | **Relative effect (95% CI)** | **Certainty of the evidence (GRADE)** | **Comments** |
| --- | --- | --- | --- | --- | --- | --- | --- |
|  |  | **Risk with No SSRIs** | **Risk with SSRIs** | **Difference** |  |  |  |
| All-cause mortality (Mortality) follow-up: range 6 months to 8.1 years | 676 (1 RCT +2 FUs) | 225 per 1 000 | **204 per 1 000** (153 to 273) | **2.1% fewer** (7.3 fewer to 4.7 more) | **RR 0.906** (0.678 to 1.210) | ⨁⨁◯◯ LOW | We are uncertain about the effect of SSRIs on mortality risk under the circumstances studied. There is a non-statistically significant tendency towards some benefit, but the 95% CI is unable to rule out either appreciable benefit or harm. |
| Cardiovascular Mortality (CV Mortality) follow-up: median 8.1 years | 300 (1 FU) | 132 per 1 000 | **107 per 1 000** (58 to 199) | **2.5% fewer** (7.5 fewer to 6.7 more) | **RR 0.811** (0.437 to 1.503) | ⨁⨁◯◯ LOW | Only one study included. We are uncertain about the effect of SSRIs on cardiovascular mortality risk under the circumstances studied. There is a non-statistically significant tendency towards some benefit, but the 95% CI is unable to rule out either appreciable benefit or harm. |
| Myocardial Infarction (MI) follow-up: range 8 weeks to 8.1 years | 1037 (5 RCTs + 1 FU) | 82 per 1 000 | **44 per 1 000** (28 to 70) | **3.7% fewer** (5.4 fewer to 1.1 fewer) | **RR 0.541** (0.341 to 0.859) | ⨁⨁◯◯ LOW | We found that the treatment with SSRIs significantly reduces the risk of MI under the circumstances studied. However, the certainty of the evidence is low.  The estimation of effect is around a 46% relative risk reduction, which corresponds to a 3.7% absolute risk reduction. |
| Angina follow-up: range 8 weeks to 6 months | 1008 (7 RCTs) | 111 per 1 000 | **84 per 1 000** (60 to 116) | **2.7% fewer** (5 fewer to 0.6 more) | **RR 0.756** (0.544 to 1.051) | ⨁◯◯◯ VERY LOW | We are uncertain about the effect of SSRIs on angina risk under the circumstances studied. Our confidence in the effect estimate is limited due to concerns regarding possible clinical heterogenicity of the definition of angina in the included studies. There is a non-statistically significant tendency towards benefit and the 95% CI seems to rule out appreciable harm (upper limit of 1.05, which would equate to a maximum plausible harm of a 5% relative increase of angina risk). |
| Hospitalizations follow-up: mean 6 months | 440 (3 RCTs) | 218 per 1 000 | **124 per 1 000** (63 to 243) | **9.4% fewer** (15.4 fewer to 2.6 more) | **RR 0.570** (0.291 to 1.118) | ⨁⨁◯◯ LOW | We are uncertain about the effect of SSRIs on hospitalization risk under the circumstances studied. There is a non-statistically significant tendency towards appreciable benefit, but the 95% CI is unable to rule out appreciable harm. |
| Stroke follow-up: range 12 weeks to 24 weeks | 728 (3 RCTs) | 8 per 1 000 | **10 per 1 000** (2 to 43) | **0.2% more** (0.6 fewer to 3.5 more) | **RR 1.266** (0.305 to 5.258) | ⨁⨁◯◯ LOW | We are uncertain about the effect of SSRIs on stroke risk under the circumstances studied. The 95% CI is unable to rule out either appreciable benefit or harm. |
| Congestive Heart Failure (CHF) follow-up: range 12 weeks to 6 months | 301 (3 RCTs) | 27 per 1 000 | **16 per 1 000** (2 to 121) | **1.1% fewer** (2.5 fewer to 9.4 more) | **RR 0.585** (0.077 to 4.473) | ⨁⨁◯◯ LOW | We are uncertain about the effect of SSRIs on CHF risk under the circumstances studied. The 95% CI is unable to rule out either appreciable benefit or harm. |
| Percutaneous Coronary Intervention (PCI) follow-up: median 8.1 years | 300 (1 FU) | 199 per 1 000 | **128 per 1 000** (75 to 216) | **7.1% fewer** (12.4 fewer to 1.7 more) | **RR 0.642** (0.378 to 1.088) | ⨁⨁◯◯ LOW | Only one study included. We are uncertain about the effect of SSRIs on PCI. There is a non-statistically significant tendency towards appreciable benefit and the 95% CI seems to rule out appreciable harm (upper limit of 1.09, which would equate to a maximum plausible harm of a 9% relative increase of PCI). |
| ***The risk in the intervention group** (and its 95% confidence interval) is based on the assumed risk in the comparison group and the **relative effect** of the intervention (and its 95% CI).  **CI:** Confidence interval; **RR:** Risk ratio  Given the clinical relevance of the outcomes assessed, we chose a 10% RR increase as a cut-off value for appreciable harm, which is more conservative than the 25% cut-off suggested by the GRADE handbook. We used a 25% RRR cut-off value for appreciable benefit. | | | | | | | |
| **GRADE Working Group grades of evidence** **High certainty:** We are very confident that the true effect lies close to that of the estimate of the effect **Moderate certainty:** We are moderately confident in the effect estimate: The true effect is likely to be close to the estimate of the effect, but there is a possibility that it is substantially different **Low certainty:** Our confidence in the effect estimate is limited: The true effect may be substantially different from the estimate of the effect **Very low certainty:** We have very little confidence in the effect estimate: The true effect is likely to be substantially different from the estimate of effect | | | | | | | |

# Supplemental Material 17 – Further Discussion of Limitations

In addition to the limitations already explored in the main text, the following are also limitations of our systematic review (several limitations would encompass more than one category, but for reasons of non-redundancy, we only included each limitation in one category; order no specific hierarchal order):

**Changes to the Protocol**

Originally, we had designed the protocol to evaluate the effect of SSRIs on the cardiovascular events in patients with depression or anxiety and coronary artery disease and had planned to do subgroup analyses based on the psychiatric diagnoses. However, given that our search did not return any studies regarding anxiety and coronary artery disease, we chose to drop “anxiety” from the target population. We did not consider “mental stress induced ischemia” to be equivalent to “anxiety”. The fact that the change we made consisted of a narrowing of the target population, it does not alter the appropriateness of the search strategy nor the rest of the systematic review.

We had initially also planned to carry out multiple subgroup analyses based on the risk of bias (low risk; some concerns; high risk; low risk + some concerns). Given the low number of RCTs included and that most of them were classified as some concerns, we decided to only carry out a “low risk + some concerns” subgroup analysis. The “high risk” and the “low risk” analyses would only consist of one RCT each, and the “some concerns” analyses would be the same as the “only post-ACS; high risk excluded” exploratory analyses.

We had also planned to, if possible, carry out analyses stratified by age, gender, ethnicity, cardiovascular risk factors, cardiac status, treatment of cardiac disorders, co-morbidities, treatment and severity of co-morbidities, severity of depression, remission/improvement or non-remission/improvement of depression. Due to the lack of raw patient data and data stratified by those characteristics, those exploratory analyses were not carried out.

In our original protocol, we had also included analyses based on SSRI choice, dosage, equivalent dosage, treatment duration, and follow-up time. Due to the small number of RCTs included that studied each SSRI, statistical power would be low and, as a result, we chose not to carry out those analyses. The same applies to the cardiac inclusion criteria (stable CAD, post-MI, or post-ACS).

We had originally also intended to analyse the composite endpoint of mortality, MI, and PCI (as used in Kim FU), however, given that no other study reported this outcome, we chose to drop it from the protocol.

In April 2020, the original search was updated and was expanded to include our revised definition of SSRIs. This decision did not influence our results, nor the conclusions drawn, given that the updated search did not result in any new studies being included. The previous search was also re-checked to ensure that, given our new definition of SSRIs, no relevant studies had been excluded in the abstract and full-text phase.

The decision to be more inclusive than the FDA definition of SSRI did not alter our results, given that only we only found relevant studies with SSRIs that are present in both the FDA and WHO lists.

No other changes to the original protocol were made.

**Clinical Heterogenicity among the studies included and differences to** **a real-world scenario**

**Population**

One study included patients with stable coronary disease (of which 64.8% of the patients had a history of myocardial infarction), five studies included only patients in the post-MI setting (and one of them only included patients after their first MI); two studies included both patients with MI and patients with unstable angina. This heterogenicity should be taken into account when interpreting our results. However, given the pre-planned exploratory analyses for the post-ACS population, we find our results relevant for those patients. In our opinion, the low number of patients and studies regarding stable CAD precludes extrapolations to the depressed stable CAD population.

Given the guideline changes regarding ACS treatment over the past decades, it is almost certain that the treatment of the ACS offered to the patients in some of the studies is not conformant to the current standards of care for ACS patients. The same applies to other chronic or acute co-morbidities patients had. Given that mortality is also determined by the accessibility to healthcare providers, quality of the medical care provided, and by levels of wealth, we cannot guaranty homogeneity between the studies in this regard. The location, and, as a result, the ethnicity of the patients included in each study also varied from study to study. The majority of patients were from Europe, North America, Australia, and Korea, which may lessen the validity of our findings for specific populations not represented or under-represented in the studies included. Also, one of the largest studies included (Kim - EsDEPACS) was a single centre study in Korea, which may caution the extrapolation of its data to other demographics. These covariates should be studied in future RCTs.

Furthermore, given that even minimal depressive symptoms have been linked to a poorer prognosis, we chose not to require a diagnosis of Major Depressive Disorder *per* the DSM, which led to heterogeneity in terms of the diagnostic criteria for depression used and the severity of the depression, which may affect the interpretation of our results (especially given that the severity of depression and non-remission of depression after ACS are associated with worse cardiovascular prognoses).

It is also of note that the patients that took part in the trials included may differ from real-world patients in that they had to agree to take part in the study (and as such may be more prone to following medical advice, or have better access to it, among other factors), didn’t fulfil any of the exclusion criteria and may have had a more thorough medical follow-up (more doctor appointments, more laboratory tests, and cardiovascular and psychiatric assessments) than patients in a real-world scenario.

**Intervention**

We assumed a class effect of SSRIs in our systematic review, due to the similarities of chemical structure and effects on depressive symptoms. However, we cannot guaranty that all SSRIs have the same effect of cardiovascular events given that not all have been properly studied to assess the effect on those outcomes. Furthermore, the fact that different SSRIs have been linked to different side effects and different rates of side effects, some of which cardiovascular (i.e. QT prolongation), may put into question the validity of class effect analyses of SSRIs for cardiovascular outcomes. As a result, the use of such analyses is a limitation.

The differences in equivalent dosages of antidepressants and antidepressant treatment duration also add to the clinical heterogenicity among the studies.

**Control**

Two of the studies included (Mohapatra and Tian) did not use a placebo control. A posthumous analysis (not reported) concluded that the inclusion of these studies did not significantly affect the primary metanalysis of Myocardial Infarction risk, not the conclusions drawn.

**Outcomes (measurement, definition, and analyses)**

We made no differences between the studies that reported the number of events and the ones that reported the number of patients with events and analysed the results jointly.

We cannot guaranty that the method for assessing the rate of cardiovascular events (self-reporting, medical records screening, …) was homogenous among studies, nor that the diagnostic criteria used for cardiovascular events were similar and the ones currently in use. In particular, the outcome “angina” may have been significantly influenced by these limitations.

The assumptions and data treatment made may be considered a limitation, however, those decisions were made before any statistical analyses were carried out and posthumous analyses (not reported) revealed that they did not impact our results and conclusions.

The long-term follow-up of EsDEPACS showed a benefit in myocardial infarction incidence when calculated in hazard ratio in the unadjusted analysis but failed to do so in the adjusted hazard ratio (only reported in the supplemental material) and when calculated in risk ratio (calculated by us). This difference in the way results may be calculated and reported did not interfere with our analyses given that for this outcome we only extracted the number of patients with events and calculated the RR ourselves.

The studies that used last observation carried forward methods for patients with incomplete data also constitute a limitation, however, the largest studies (Glassman FU, Kim FU, and Lespérance) had complete outcome data for almost every patient.

The outcome of stroke was the only one that trended towards harm, however, given that only 7 events occurred (out of 728 patients) and that the 95% CI is wide (0.31-5.26), no conclusions should be drawn from this trend.

**Study design and follow-up time**

The follow-up time of most of the RCTs included, although appropriate to analyse short term depressive symptom related outcomes, is noticeably insufficient to assess cardiovascular outcomes, which is a major limitation of our review. Given that we aimed to conduct analyses stratified by treatment duration and follow-up time, we chose not to require a minimum follow-up time or treatment duration. However, due to the paucity of studies included, those exploratory analyses were not carried out.

The inclusion of FUs increases the risk of bias of our analysis given that in the observational phase no *de facto* intervention nor control were used; and the patients were aware of their previous allocation in the RCT phase (Kim FU refers that the patients were unblinded at the end of the RCT phase, and, even though Glassman FU doesn’t, we assumed those patients were unblinded, according to what is customary in RCTs), which may have influenced patients' treatment choices during the observational phase (Glassman FU doesn’t report the rate of antidepressant use during the extended observational phase; Kim FU reports the rate of antidepressant use at the 1-year point, which was 5/149 for the SSRI group and 3/151 for the control group but doesn’t report the rate of SSRI use for the remainder of the extended follow-up period).

However, given that the FU study methodology is much more akin to a pragmatic open-label RCT than to observational studies (given that the allocation to intervention or control was randomized), we decided to, for purposes of our primary quantitative analyses, consider them similar to RCTs and analyse the studies jointly. We consider that, for that reason, this decision did not substantially affect the validity of our findings.

**Risk of bias and quality of evidence analyses**

**RoB2**

The RoB2 assessment of the studies included was carried out in a conservative fashion with few assumptions made, therefore increasing the burden of proof for “low risk” rating. In the cases of indecision between two ratings, we chose to downgrade.

Given the lack of a Cochrane tool to analyse the risk of bias of FUs, and the close similarities to a pragmatic open-label RCT study design, we decided to assess the risk of bias of the FUs using the RoB2 tool, instead of the Robins-I tool. We considered that the intervention/control for the FUs was “24 weeks of double-blind SSRI/placebo followed by tapering down of study medication, unblinding, and then treatment as usual provided by their physicians outside of the trial (according to what they considered necessary for each patient)”. Glassman refers that if further treatment was necessary, patients were referred to appropriate care. Under this definition, the FUs scored “low risk” in all domains, however, given that we cannot rule out confounding factors during the extended observational phase of the study (for example differences in the care delivered after unblinding the patients, such as a different rate of antidepressant use or other interventions that improve their prognosis, that might have arisen from the patients’ or their physicians’ knowledge of their previous allocation), we decided to downgrade the FUs to “some concerns”. Had we incorporated in our intervention/control definitions for the RoB2 analyses that the care as usual provided to both arms had to be the same in order to avoid confounding factors, the answer to the question 2.3 would have been NI, which would have led to a “some concerns” rating for the risk of bias due to deviations from the intended interventions domain, as well as for the overall rating. We decided to define the intervention/control the way we did for purposes of risk of bias assessment because it better represents the methodology of the FUs included and better reproduces a real-world scenario. Nevertheless, this decision didn’t affect the overall rating of the FUs’ risk of bias.

These decisions were subjective and as such could be considered by some to be limitations of our systematic review.

**GRADE assessment**

The use of different cut-off values for appreciable benefit and harm may be considered a limitation by some. However, we decided to have a sterner threshold for appreciable harm (10% RR increase, instead of the 25% cut-off suggested by the GRADE handbook), because of the seriousness and clinical relevance of the outcomes assessed. We used a 25% RRR threshold for appreciable benefit given that we wanted to maintain the stricter criteria for appreciable benefit suggested by the GRADE handbook. In our opinion, this decision strengthens the interpretation of the results of our metanalyses, as it demands a higher the burden of proof for appreciable benefit compared to appreciable harm of the use of SSRIs in the studied setting.

Most of the studies included had an uncertain risk of bias. The GRADE handbook leaves room for subjectivity in terms of whether an “uncertain” risk of bias rating should downgrade the quality of evidence or not. We took a conservative approach and downgraded the quality of the evidence.

The differences in population characteristics among the studies and when compared to a real-world scenario (see above) were considered not substantial enough to downgrade the certainty of the evidence. The differences in intervention were not considered to be substantial enough to downgrade the certainty of the evidence (as we assumed a class effect of SSRIs). The definition of the outcomes and the method used to assess the occurrence of events was not considered to be substantial enough to downgrade the certainty of the evidence (except for the outcome of angina); no surrogate outcomes nor indirect comparisons were used.

In terms of publication bias, we did not create nor assessed funnel plots due to the small number of studies included for each outcome. We cannot guaranty that small RCTs were carried out but not published, however, our search was comprehensive and even included registries of RCTs, our exclusion process in the title and abstract phase was conservative, and we even retrieved an unpublished study. The properness of our search and section process is further supported by having included all suitable articles present in other previous systematic reviews^20,21^ and by cross-referencing information from the included articles regarding other similar trials carried out. As a result, publication bias risk was not considered high enough to warrant downgrading the quality of the evidence.

These decisions were subjective and as such could be considered by some to be limitations of our systematic review.

**Analyses not reported**

The posthumous analyses that assessed the influence of the assumptions and data treatment made as well as the influence of the inclusion of no intervention as a control group are not reported in the article or the supplemental material because (1) they were not pre-planned, (2) didn’t change the results of our analyses or their interpretations, and (3) to avoid needlessly extending the number of results presented. The data used in the analyses is available in the Supplemental Material 6 (Relevant outcomes extracted from the studies included), and, as such, if the readers wish to do so, they are able to calculate the results of those unreported analyses themselves. Alternatively, the authors will also provide those posthumous analyses upon reasonable request.

Despite these limitations, we consider that our review was conducted in a proper fashion and that most if not all the limitations that may have influenced our results or their interpretation were somewhat inevitable and will also influence future systematic reviews on this issue, given the characteristics of the RCTs and FUs carried out on the cardiovascular effects of SSRIs in depressed CAD patients.

# Supplemental Material 18 – PRISMA Checklist

| **Section/topic** | **#** | **Checklist item** | **Reported on page #** |
| --- | --- | --- | --- |
| **TITLE** | | |  |
| Title | 1 | Identify the report as a systematic review, meta-analysis, or both. | 1 |
| **ABSTRACT** | | |  |
| Structured summary | 2 | Provide a structured summary including, as applicable: background; objectives; data sources; study eligibility criteria, participants, and interventions; study appraisal and synthesis methods; results; limitations; conclusions and implications of key findings; systematic review registration number. | 2-3 |
| **INTRODUCTION** | | |  |
| Rationale | 3 | Describe the rationale for the review in the context of what is already known. | 4 |
| Objectives | 4 | Provide an explicit statement of questions being addressed with reference to participants, interventions, comparisons, outcomes, and study design (PICOS). | 4 |
| **METHODS** | | |  |
| Protocol and registration | 5 | Indicate if a review protocol exists, if and where it can be accessed (e.g., Web address), and, if available, provide registration information including registration number. | Not Available |
| Eligibility criteria | 6 | Specify study characteristics (e.g., PICOS, length of follow-up) and report characteristics (e.g., years considered, language, publication status) used as criteria for eligibility, giving rationale. | 5 + SM |
| Information sources | 7 | Describe all information sources (e.g., databases with dates of coverage, contact with study authors to identify additional studies) in the search and date last searched. | 5 + SM |
| Search | 8 | Present full electronic search strategy for at least one database, including any limits used, such that it could be repeated. | SM |
| Study selection | 9 | State the process for selecting studies (i.e., screening, eligibility, included in systematic review, and, if applicable, included in the meta-analysis). | 5-7 + SM |
| Data collection process | 10 | Describe method of data extraction from reports (e.g., piloted forms, independently, in duplicate) and any processes for obtaining and confirming data from investigators. | 5-6 |
| Data items | 11 | List and define all variables for which data were sought (e.g., PICOS, funding sources) and any assumptions and simplifications made. | 5-6 + SM |
| Risk of bias in individual studies | 12 | Describe methods used for assessing risk of bias of individual studies (including specification of whether this was done at the study or outcome level), and how this information is to be used in any data synthesis. | 6 + SM |
| Summary measures | 13 | State the principal summary measures (e.g., risk ratio, difference in means). | 6 |
| Synthesis of results | 14 | Describe the methods of handling data and combining results of studies, if done, including measures of consistency (e.g., I^2^) for each meta-analysis. | 6 |

| **Section/topic** | **#** | **Checklist item** | **Reported on page #** |
| --- | --- | --- | --- |
| Risk of bias across studies | 15 | Specify any assessment of risk of bias that may affect the cumulative evidence (e.g., publication bias, selective reporting within studies). | Only GRADE carried out: 7 |
| Additional analyses | 16 | Describe methods of additional analyses (e.g., sensitivity or subgroup analyses, meta-regression), if done, indicating which were pre-specified. | 6 |
| **RESULTS** | | |  |
| Study selection | 17 | Give numbers of studies screened, assessed for eligibility, and included in the review, with reasons for exclusions at each stage, ideally with a flow diagram. | 8 + Fig. 1 + SM |
| Study characteristics | 18 | For each study, present characteristics for which data were extracted (e.g., study size, PICOS, follow-up period) and provide the citations. | 8 + Table 1 + SM |
| Risk of bias within studies | 19 | Present data on risk of bias of each study and, if available, any outcome level assessment (see item 12). | 8-9 + Table 1 + SM |
| Results of individual studies | 20 | For all outcomes considered (benefits or harms), present, for each study: (a) simple summary data for each intervention group (b) effect estimates and confidence intervals, ideally with a forest plot. | 9-10 + Fig. 2 + SM |
| Synthesis of results | 21 | Present results of each meta-analysis done, including confidence intervals and measures of consistency. | 9-10 + Fig. 2 + SM |
| Risk of bias across studies | 22 | Present results of any assessment of risk of bias across studies (see Item 15). | Only GRADE carried out: 10 + Table 2 +SM |
| Additional analysis | 23 | Give results of additional analyses, if done (e.g., sensitivity or subgroup analyses, meta-regression [see Item 16]). | 9-10 + SM |
| **DISCUSSION** | | |  |
| Summary of evidence | 24 | Summarize the main findings including the strength of evidence for each main outcome; consider their relevance to key groups (e.g., healthcare providers, users, and policy makers). | 11-12 |
| Limitations | 25 | Discuss limitations at study and outcome level (e.g., risk of bias), and at review-level (e.g., incomplete retrieval of identified research, reporting bias). | 12-14 + SM |
| Conclusions | 26 | Provide a general interpretation of the results in the context of other evidence, and implications for future research. | 15 |
| **FUNDING** | | |  |
| Funding | 27 | Describe sources of funding for the systematic review and other support (e.g., supply of data); role of funders for the systematic review. | 16 |

SM – Supplemental Material; Page number refer to the submitted manuscript, not the published article.

*Based on:*  Moher D, Liberati A, Tetzlaff J, Altman DG, The PRISMA Group (2009). Preferred Reporting Items for Systematic Reviews and Meta-Analyses: The PRISMA Statement. PLoS Med 6(7): e1000097. doi:10.1371/journal.pmed1000097
